# Multi-scale phylodynamic modelling of rapid punctuated pathogen evolution

**DOI:** 10.1371/journal.pcbi.1013295

**Published:** 2025-07-14

**Authors:** Quang Dang Nguyen, Sheryl L. Chang, Carl J. E. Suster, Rebecca J. Rockett, Vitali Sintchenko, Tania C. Sorrell, Mikhail Prokopenko

**Affiliations:** 1 Centre for Complex Systems, The University of Sydney, Sydney, New South Wales, Australia; 2 Sydney Infectious Diseases Institute, The University of Sydney, Sydney, New South Wales, Australia; 3 Centre for Infectious Diseases and Microbiology–Public Health, Westmead Hospital, Westmead, New South Wales, Australia; University Hospital Zurich, SWITZERLAND

## Abstract

Computational multi-scale pandemic modelling remains a major and timely challenge. Here we identify specific requirements for a new class of models simulating pandemics across three scales: (1) pathogen evolution, often punctuated by the rapid emergence of new variants, (2) human interactions within a heterogeneous population, and (3) public health responses which constrain individual actions to control the disease transmission. We then present a pandemic modelling framework satisfying these requirements and capable of simulating feedback loops between dynamics unfolding at these different scales. The developed framework comprises a stochastic agent-based model of pandemic spread, coupled with a phylodynamic model that incorporates within-host pathogen evolution. It is validated with a case study, modelling the punctuated evolution of SARS-CoV-2, based on global and contemporary genomic surveillance data, which captures a large heterogeneous population. We demonstrate that the model replicates the essential features of the COVID-19 pandemic and virus evolution, while retaining computational tractability and scalability.

## Author summary

Accurate modelling of pandemic spread is highly challenging due to the unpredictable nature of emerging and evolving pathogens, the diversity of populations with unevenly distributed risks, and the dynamic public health interventions that adapt to rapidly changing situations. In this work, we developed a novel computational framework designed to capture the complexities of infectious disease transmission across heterogeneous populations, accounting for the impact of public health measures. Additionally, our model incorporates a phylodynamic approach to simulate the evolution of pathogens within individual hosts. By integrating contemporary genomic, demographic, and mobility data, we validated our model through a case study that replicated key characteristics of the recent COVID-19 pandemic. Notably, our simulation, in line with real-world observations, demonstrated the punctuated evolution of SARS-CoV-2 and the emergence and spread of various viral variants. Specifically, the model linked pathogen evolution to the dynamics of social interactions and the effects of public health interventions, showcasing the power of multi-scale modelling in exploring the complexities of pandemic scenarios.

## Introduction

Digital epidemiology is an emerging field, rapidly capitalising on the increasing availability of high-resolution genomic, immuno-epidemiological, demographic and human mobility data, social media analytics, high-performance computing power, as well as innovations in simulation methods and data science. In principle, these diverse sources of data should allow modellers to develop informative multi-scale pandemic models with a better capacity (i) to simulate realistic epidemiological, immunological and evolutionary dynamics, and (ii) to anticipate longer-term epidemiological and evolutionary dynamics as the pandemic unfolds. Yet, pandemic modelling continues to face significant challenges in concisely capturing relevant characteristics of pandemic pathogens, such as their pathogenesis, transmissibility, antigenicity, etc., as well as predicting long-term phylodynamic trajectories and future public health risks. These challenges arise due to (i) the inherent complexity of rapidly evolving pathogens, (ii) population heterogeneity (demographic, immunological and behavioural), and (iii) multi-objective public health interventions carried out under severe pressure and non-trivial social dynamics.

The modelling complexity is exacerbated by (iv) different time scales needed to model pandemics on multiple levels, ranging from the range of evolutionary drivers and rates of the implicated pathogens to natural infections in individuals and their social behaviour and interactions, (v) fragmentation of data across heterogeneous sources, and (vi) computational complexity of multiple simulations over a sufficiently long horizon, required to examine the distribution of outcomes in many stochastic realisations of the model and across ranges of uncertainties in parameters such as substitution rate, fitness, accumulated mutations, and genomic diversity. Multi-scale models often suffer from the “curse of dimensionality”, when computational costs increase exponentially with the number of degrees of freedom [1].

A principal modelling problem is the presence of feedback loops: for example, pandemic mitigation measures may indirectly affect the pathogen evolution, leading to the emergence of more transmissible lineages. Higher transmissibility may increase the need for more vigorous interventions, which in turn may cause changes in how populations respond and behave, and constrain the pathogen evolution in a specific way. This feedback loop contributes to the formation of recurrent waves of infection, fluctuating genomic diversity, non-linear increases in fitness levels, and a potentially delayed transition to endemicity. Consequently, multi-scale modelling of a major pandemic crisis, such as COVID-19, quickly becomes intractable.

Over the last decades, stochastic agent-based modelling (ABM) has been established as a robust tool for tracing fine-grained effects of complex intervention policies in diverse epidemic and pandemic settings [2–5]. Most recently, these studies produced policy recommendations developed for COVID-19 control, which were adopted in Australia [6–8], the USA [9], the UK [10], and broadly by the WHO [11]. In these ABMs, each agent represents an individual human host with a set of demographic, epidemiological, and immunological attributes. A largely unexplored avenue to leverage the precision and fidelity of ABMs is to extend them with comprehensive phylodynamic modelling of evolving pathogens, going beyond existing phylogenetic models which define simplified evolutionary landscapes [12,13]. This necessitates a new class of multi-scale phylodynamic ABMs.

An effective framework for multi-scale phylodynamic agent-based modelling should include the following distinct capabilities that produce quantifiable outcomes:

Capability 1: Model and examine epidemic or pandemic patterns over a mid- to long-term timeframe, with respect to complex transmission and immunological profiles, affected by varying pharmaceutical and non-pharmaceutical interventions:
  (a) Reproduce and predict salient peaks and recurrent waves of incidence, prevalence, and other epidemic dynamics.
  (b) Explore possible transitions and pathways to endemicity or elimination.
Capability 2: Examine the pathogen fitness with respect to its phylodynamics:
  (a) Trace changes in transmissibility with respect to pathogen mutations. This includes analysis of the average reproductive number within the population, and the accumulated mutations measured by the average genomic distance between a circulating genome and the ancestral genome, e.g., in terms of the nucleotide substitution rate.
  (b) Examine the functional dynamics (i.e., the relationship between the genome sequence and the pathogen’s epidemic behaviour). This analysis quantifies the individual contributions of amino acids to changes in the pathogen’s fitness, and traces the dynamics of these contributions to transmissibility and antigenicity over time.
Capability 3: Detect and evaluate the emergence and dominance of variants of concern:
  (a) Explore concordance between phylodynamics and disease dynamics, e.g., relating accumulated mutations, genomic diversity, and saltations in pathogen fitness to incidence peaks.
  (b) Detect abrupt changes in genomic diversity, and evaluate emergence of variants of concern, by using appropriate quantitative techniques (such as Augmented Dickey-Fuller stationarity test, CUSUM, etc.), supported by suitable data visualisation methods (such as phylogenetic trees).

**Fig 1:**
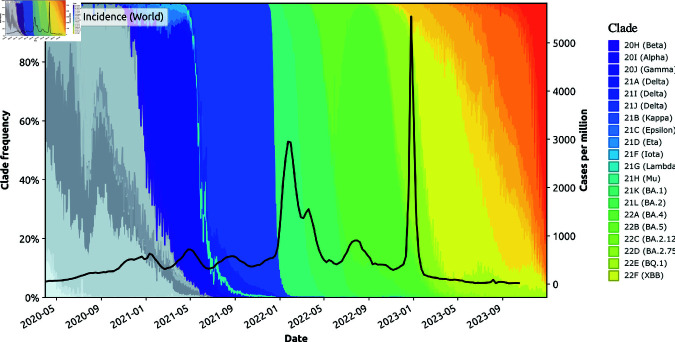
Capability 1. Pandemic patterns in terms of the worldwide incidence (solid, black line), measured as new weekly cases per million [14], overlaid with the frequency of circulating variants between 2020 and 2024, plotted using open SARS-CoV-2 sequence data (7,075,645 samples) from GenBank and the Robert Koch Institute, processed by Nextstrain [15].

**Fig 2:**
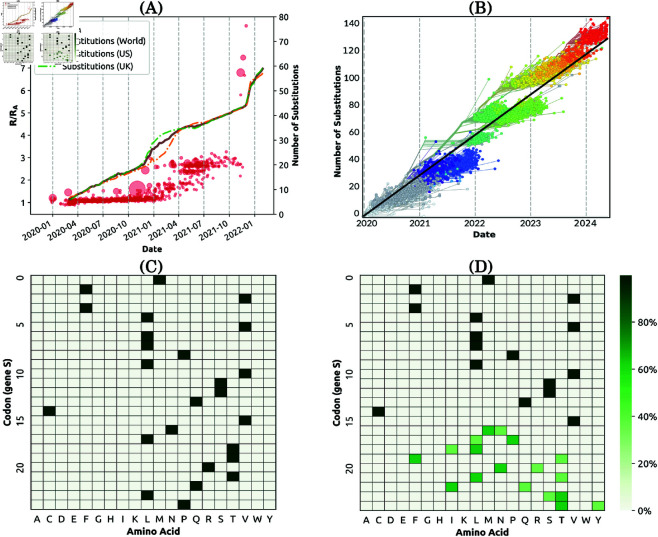
Capability 2. Pathogen fitness with respect to its phylodynamics. (A) Punctuated growth of fitness, measured relative to the basic reproductive number of the ancestral strain *R*_*A*_ [17] (represented as the *R*/*R*_*A*_, red circles), overlaid with the average accumulated nucleotide substitutions across the population (represented by the Hamming distance $\hat{D}$), also measured relative to the ancestral strain. (B) A time-scaled phylogeny of representative SARS-CoV-2 sequences mapped onto the number of accumulated substitutions relative to the ancestral genome. We refer to Fig 1 for clade colours. For visualisation purposes, we show around 4,000 genomes. Data retrieved from Nextstrain [18]. (C, D) Heatmaps of amino acids over the first 25 codons in gene S of the COVID-19 ancestral strain (C), and 631 randomly selected genomes from GenBank and the Robert Koch Institute between 17 and 31 December 2023 (D) [15].

**Fig 3:**
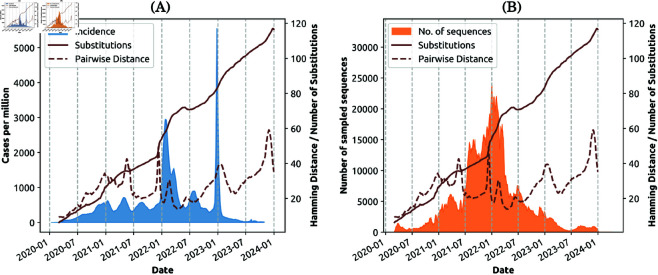
Capability 3 (i). Temporal alignments between phylodynamics and disease dynamics. (A) The global COVID-19 incidence (new weekly cases per million, shaded blue area) overlaid with accumulated mutations $\hat{D}$ (represented by the average distance between circulating genomes and the ancestral strain, solid brown line) and genomic diversity $\overset{―}{D}$ (represented by the average pairwise distance between two randomly selected genome sequences, dashed brown line). (B) The number of sequences, measured using a 7-day moving average, captured by the open SARS-CoV-2 sequence database processed by Nextstrain [15] (shaded orange area) overlaid with accumulated mutations $\hat{D}$ across the genome (solid brown line), and genomic diversity $\overset{―}{D}$ (dashed brown line).

**Fig 4:**
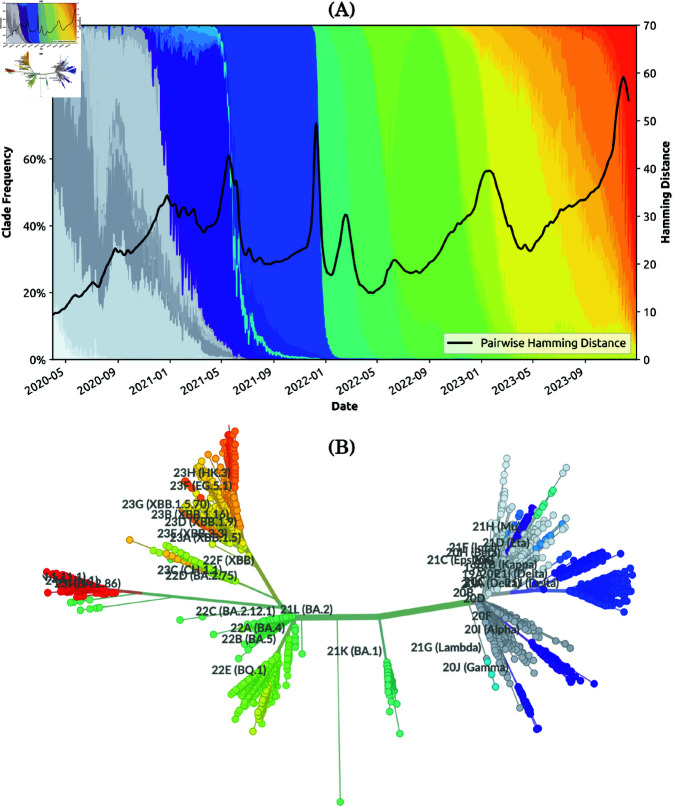
Capability 3 (ii). Emergence and dominance of variants of concern. (A) Genomic diversity $\overset{―}{D}$ (i.e., the average pairwise distance between two randomly selected genome sequences on a given day, solid black line) overlaid with the clade frequency between 2020 and 2024. (B) Phylogenetic tree of SARS-CoV-2, built with Nextstrain from worldwide sequences collected between 2020 and 2024 [18]. The phylogenetic tree is constructed from approximately 4,000 genomes.

A robust phylodynamic ABM needs to be validated by comparing its target outcomes against the ground truth dynamics. Once validated, it can be used to explore diverse counterfactual scenarios with respect to phylodynamic, demographic and immuno-epidemiological characteristics. We illustrate the ground truth dynamics, matching the three capabilities, in Figs 1 to 4, by using available genomic and disease surveillance data on SARS-CoV-2 and COVID-19 respectively from 2020 to 2024 (we note that the detected incidence is affected by the testing capacity).

To demonstrate **Capability 1**, a modelling framework is expected to generate pandemic or epidemic patterns aligned with ground truth (i.e., observed data). For example, Fig 1 shows patterns observed in global data reported during the COVID-19 pandemic, highlighting prominent peaks and recurrent incidence waves. We point out that each incidence peak is temporally aligned with the emergence of a new variant of concern. Notably, the two most prominent incidence peaks occurred in early 2022 and early 2023, corresponding to the dominance of Omicron BA.1 and Omicron XBB, respectively. These observations suggest a complex interplay between pandemic patterns and evolutionary phylodynamic features of the viral variants in circulation.

**Capability 2** demands a dynamic analysis of the pathogen fitness in terms of observable phylodynamic characteristics. For example, Fig 2 relates the growing transmissibility of SARS-CoV-2 to (i) the mutations accumulated relative to its ancestral strain, and (ii) the associated genomic diversity of the evolved pathogen. It also places the fitness dynamics in the context of the genomics.

Punctuated evolution of the novel coronavirus was observed even during the first year of the pandemic [16]. Fig 2A shows a rapid punctuated increase in fitness for the first two years of the pandemic, with two significant surges in the relative transmissibility (measured relative to the basic reproductive number of the ancestral strain *R*_*A*_), and the accumulated mutations, observed during early 2021 and early 2022. The accumulated mutations continued to grow after 2022, reaching 130 substitutions by mid-2024 at the rate of roughly 30 substitutions per year, according to linear regression (Fig 2B).

Furthermore, notable changes in the amino acid presence were observed across the genome, particularly the spike region, between the start of the pandemic and late 2023, as illustrated for the 5^′^ end of the S gene in Fig 2C–2D. These shifts occurred preferentially at specific positions, deviating significantly from the ancestral genome, and thus, potentially contributing to the increase in fitness.

**Capability 3** is focused on the emergence and dominance of variants of concern, in the context of phylodynamic and epidemiological dynamics. Two quantities can be observed on any given day: the average mutations accumulated by the evolved genomes relative to the ancestral strain, denoted $\hat{D}$; and the average pairwise distance among the genomes of co-circulating pathogens on that day, denoted $\overset{―}{D}$ (see Materials and methods). For example, Fig 3A traces a continuous increase in the accumulated mutations which is contrasted with the fluctuating genomic diversity. Unlike the mutations $\hat{D}$ which accumulated relative to the ancestral strain, the genomic diversity, measured as the daily pairwise distance $\overset{―}{D}$, shows only a marginal increase during the observed period. We extracted phylodynamic features using the data recorded until early 2024, noting a significant drop in the number of sequenced samples worldwide from 2024 onwards (Fig 3B).

In general, abrupt changes in genomic diversity are related to the frequency of different variants reported during the pandemic period (Fig 4A), and considered in context of the corresponding phylogenetic tree (Fig 4B). For example, we observed that during the rapid evolution of SARS-CoV-2, sudden decreases in circulating diversity correspond to specific lineages becoming dominant, whereas new variants are more likely to emerge during periods of increasing circulating diversity (i.e., increasing pairwise genomic distance). Importantly, the observed changes in the pairwise distance are also reflected in the phylogenetic tree produced from representative sub-sampling of global sequences (Fig 4B). Branches corresponding to new variants of concern tend to descend from more basal lineages than the main lineages circulating immediately prior to their emergence. These evolutionary saltations may explain step changes in transmissibility and virulence.

## Results

We present an agent-based modelling (ABM) framework for computational modelling of pathogen phylodynamics, focusing on communicable diseases within heterogeneous populations. The key component is PhASE TraCE, **Ph**ylodynamic **A**gent-based **S**imulator of **E**pidemic **Tra**nsmission, **C**ontrol, and **E**volution, a versatile multi-scale simulator for modelling rapid pathogen evolution. The framework also includes several phylodynamic measures aimed to identify the emergence of novel pathogen variants.

PhASE TraCE was developed upon several existing large-scale pandemic simulators, including the Australian Census-based Epidemic Model (AceMod) of pandemic influenza [19–21], and the Agent-based Model of Transmission and Control of the COVID-19 pandemic in Australia (AMTraC-19) [6,22]. These ABMs have been successfully validated and used in simulating multiple waves of influenza [19] and COVID-19 [5,6,23–25], mitigated by various interventions, including mass-vaccination roll-outs and non-pharmaceutical interventions.

Similar to these models, PhASE TraCE simulates the disease transmission in discrete time in an artificially generated population with census-based demographic characteristics and commuting patterns [26]. Going beyond the existing models, PhASE TraCE is capable of simulating the inter-host transmission of multiple pathogen variants within a heterogeneous population, the within-host evolution of pathogens, and immuno-epidemiological feedback.

In this section, we overview the model’s multi-scale approach (subsection Model overview). We then apply PhASE TraCE to a case study of SARS-CoV-2, and evaluate the simulated phylodynamics against the modelling capabilities and objectives (subsection Case study: Rapid punctuated evolution of SARS-CoV-2).). Finally, we explore counterfactual scenarios by varying specific assumptions of the case study, e.g., the role of chronic infections and population sizes (S1 Text: Counterfactual modelling).

### Model overview

Distinct from many computational models that focus solely on epidemiological dynamics within host populations or evolutionary dynamics within pathogen populations, PhASE TraCE simulates dynamic feedback across three scales: (i) micro-scale: within-host evolution and the evolutionary landscape of circulating variants; (ii) meso-scale: agent-to-agent interactions and inter-host transmission; and (iii) macro-scale: public health interventions (i.e, non-pharmaceutical interventions and vaccination) at the population level.

**Fig 5:**
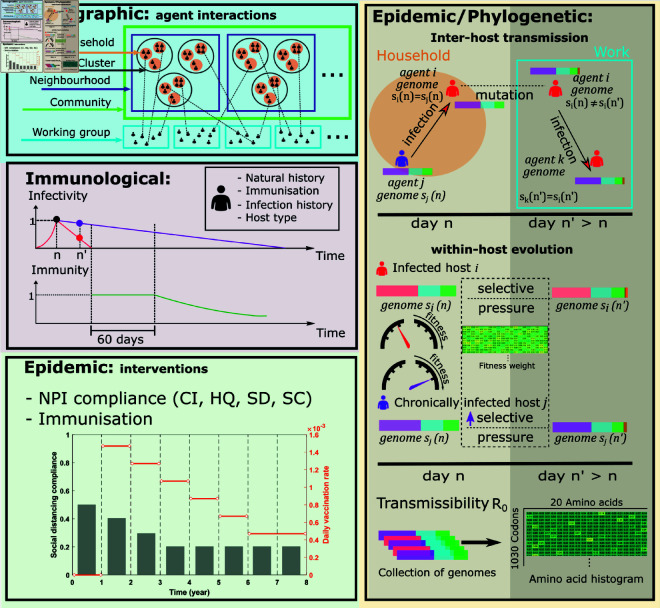
Model overview. The demographic layer assigns agents to social contexts, including household, household cluster, neighbourhood, community, and working group/school. The immunological layer keeps track of the agents’ immunisation records, infection histories, and the host types. An example of the infectivity profiles is illustrated for a typical infected host (red line) and a chronically infected host (purple line). On day *n*, the infectivity peaks (i.e., reaches 1.0) for both host types (black dot). On day $n^{\prime }>n$, the infectivity of the chronically infected host (purple dot) reduces more slowly than that of the typical infected host (red dot). Upon recovery, the typical infected host attains perfect immunity of 1.0 for 60 days, after which the immunity starts to decline (green line). The epidemic layer describes an intervention scenario, in terms of NPIs and their adoption levels: case isolation (CI), home quarantine (HQ), social distancing (SD), and school closure (SC), along with a dynamic immunisation schedule. Both epidemic and phylogenetic layers affect inter-host transmission (e.g., on day *n*, a chronically infected host *j* infects their family member *i* and passes genome *s*_*j*_(*n*) to agent *i*). The phylogenetic layer determines the genomic mutations under various strengths of selective pressure, and traces changes in transmissibility (reflected in reproductive number). The fitness of each genome is determined by a weight table, quantifying the contributions of each amino acid in a given codon position.

This multi-scale feedback is realised by incorporating four concurrent dynamics implemented in distinct processing layers: demographic, epidemic, immunological, and phylogenetic, as illustrated in Fig 5. The demographic layer defines the population structure (i.e., host type) and social groups constraining agent interactions. The transmission is simulated by the epidemic layer, tracing individual interactions occurring within and across different social contexts, including residential (e.g., household) and professional settings (e.g., working group). In addition, the epidemic layer sets out an intervention scenario, with varying NPI adoption levels and vaccination strategies, thus reflecting changes in health policy and public opinion [27,28]. The infection transmission depends on the agents’ immunity levels derived from their individual dynamic history of exposures and immunisation, determined by the immunological layer. There are two infected host categories: typical infected hosts and chronically infected hosts, with the latter representing hosts with *persistent* SARS-CoV-2 infections arising due to some underlying factors and resulting in longer recovery periods [29,30] (see S1 Text: Infected host categories). The phylogenetic layer determines the within-host evolution of pathogens in terms of mutation and selective pressure. Section Materials and methods provides a detailed description and implementation of these four layers.

### Case study: Rapid punctuated evolution of SARS-CoV-2

PhASE TraCE was calibrated to the COVID-19 pandemic and the evolutionary trajectory of SARS-CoV-2 over the period of four years, from 2020 to 2023. The calibration explored ranges of over 90 parameters in four categories corresponding to modelling layers. Demographic parameters were census-calibrated [19,20,25,26]. Most of the epidemiological and immunological parameters were calibrated in our prior studies [6,24,25,27]. For example, parameter ranges related to non-pharmaceutical interventions were established based on prior studies [6,24,25,27] and public health policies during the COVID-19 pandemic. In calibrating the natural history of the disease for this work, we used contemporary evidence, including studies of chronic infections [30,31]). In contrast, all phylogenetic parameters were specifically calibrated for this study. The majority of phylogenetic parameters were determined using reported genomic evidence [18] and recent phylogenetic analyses [17,32–36]. Some phylogenetic inputs that are specific to our model, such as the weight table quantifying the fitness contribution of amino acids, were calibrated by comparing the simulation outcomes corresponding to different parameterisations and selecting the most fitting parameterisations. The phylogenetic parameters are summarised in S4 Table.

The results comprise simulations over a six-year period (2020–2025), across three artificial agent populations: 1.7 million agents (small), 8 million agents (medium), and 25.4 million agents (large), as described in S1 Text (Artificial agent-based population).

In calibrating PhASE TraCE, we examined COVID-19 pandemic and the size of susceptible, recovered and vaccinated populations (Capability 1); phylodynamic characteristics including transmissibility, fitness, the number of accumulated mutations relative to the ancestral strain, and genomic diversity (Capability 2); and the emergence of variants, analysed using the phylogenetic tree and statistical methods (Capability 3). The robustness of the model is established through sensitivity analysis, by varying key parameters and assessing their impact on simulation outcomes. See S4 Table for the list of phylogenetic parameters used in the SARS-CoV-2 case study and S1 Text (Sensitivity analysis) for more information on sensitivity analysis.

**Fig 6:**
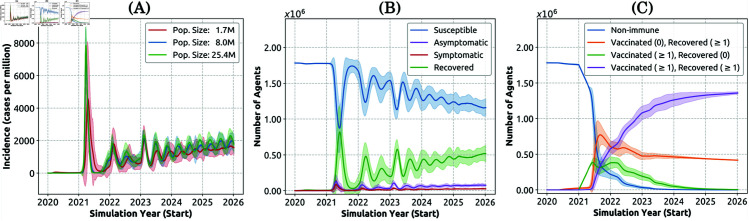
Simulated pandemic patterns (Capability 1) shown as mean (solid line) and standard deviation (shaded area). (A) Detected incidence simulated in population sets of 1.7 million (red), 8 million (blue), and 25.4 million (green). (B) Population in different health states, including susceptible (blue), asymptomatically infected (purple), symptomatically infected (red), and recovered (green). (C) Population with different immunisation and infection history. Numbers in brackets denote the number of vaccination or infection records. Individuals with multiple vaccinations or infections (more than 2) are grouped together for simplicity. (B) and (C) are generated using the population set of 1.7 million. Mean and standard deviation were obtained from approximately 30-50 realisations.

**Capability 1.** Our results show recurrent incidence waves as illustrated in Fig 6A and S7 Fig, in concordance with empirical observations (Fig 1 and S7 Fig). Although the first simulated incidence peak (detected during 2021) is noticeably higher in larger populations, the incidence converged to around 2,000 cases per million after 2023, indicating endemicity in all simulated populations.

The main contributing factor to the recurrent incidence is the fluctuating number of recovered individuals (Fig 6B) that replenish the susceptible population after their immunity wanes. This subsequently leads to an increasing number of re-infection cases. Additionally, due to the diminishing immunity, declining vaccine uptake, and declining adoption of social distancing in the population (simulated to be decreasing from 50% of hosts in 2020 to 20% from 2024 onwards), the overall incidence slightly increases over the simulated timeframe after the first peak (Fig 6A). Notably, simulation results suggest that chronic infections strongly affect the magnitude of incidence oscillations (S16 Fig). This finding may also explain the dampened oscillations observed in smaller populations. Emerging strains tend to dominate more strongly across a smaller population, resulting in: (i) reduced genomic diversity of the pathogen, (ii) a more homogeneous and consistent immune response across the population, and consequently (iii) a smoother incidence profile (see discussion of Capability 3 below).

We also observe an increasing number of individuals with multiple vaccination and infection records (Fig 6C). These dynamics slow down the transmission but are unable to completely eliminate the spread, due to the waning immunity and reduced vaccine efficacy against mutated variants.

Figs 6B and 6C only show dynamics for the 1.7 million population. We note that the pandemic patterns are consistent across different population sizes, as shown in S8 Fig.

**Capability 2.** Here we investigate how the pathogen fitness changes during the simulation, given the selective pressure on circulating strains which “compete” in terms of their transmissibility. We begin by exploring how the phylodynamic characteristics, such as accumulated mutations, develop along the observed increase in simulated fitness (Capability 2 (i)).

**Fig 7:**
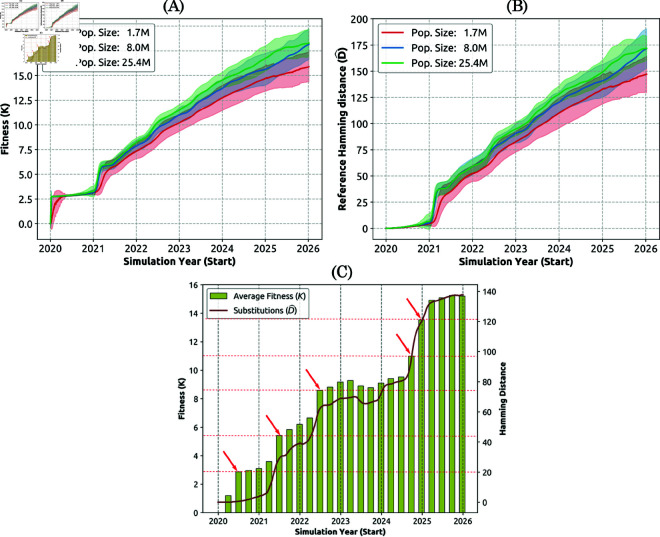
Simulated phylodynamic measures (Capability 2(i)), shown as mean (solid line) and standard deviation (shaded area) for different populations: 1.7 million (red), 8 million (blue), and 25.4 million (green). (A) Growing pathogen transmissibility, interpreted as fitness *K*. (B) Accumulated mutations $\hat{D}$, measured at each time point by the average reference Hamming distance between the circulating variants and the reference (ancestral) variant. The mean and average were obtained from approximately 30-50 realisations. (C) Single realisation, showing alignment of the average transmissibility, i.e., fitness (*K*, olive bars), and the accumulated mutations ($\hat{D}$, solid brown line). The most prominent saltations are marked by red arrows, and the corresponding fitness levels are marked by red horizontal dashed lines.

Importantly, we observe a concurrent punctuated increase (i.e., ‘jump’) in both transmissibility, i.e., fitness *K* (Fig 7A), and accumulated mutations $\hat{D}$ (Fig 7B) at the start of 2021. This is a pattern which is also observed in empirical data illustrated in Fig 2A. The timing of the simulated ‘jump’ aligns well with the first incidence peak in 2021, illustrated in Fig 6A. This coincidence can be explained by the accumulation of fitness-increasing mutations in chronically infected hosts, due to a higher selective pressure on pathogens evolving in these hosts; consequently generating a highly transmissible strain (i.e., a variant of concern) (see S1 Text: Infected host categories). This is confirmed by a comparison with a counterfactual scenario without chronically infected hosts in which the jump is not observed, as shown in S16 Fig and analysed in S1 Text (Counterfactual modelling). We also traced the fitness contributions of different genomic regions, by computing the fitness per codon (S1 Fig). Evidently, the spike and epitope regions, which contribute to both transmissibility and antigenicity, have the highest fitness *K* per codon (S12 Fig) and the highest number of accumulated mutations $\hat{D}$ per codon (S12 Fig). Notably, this high contribution is observed despite the short genome length of this region (45 codons), indicating that the fitness gain is a result of combining the increased transmissibility and the higher immune escape. In comparison, the spike and non-epitope region (55 codons) which contributes only to transmissibility has lower fitness *K* and accumulated mutations $\hat{D}$ per codon.

Similar jumps are observed at later pandemic stages in a majority of individual simulation runs (see Figs 7C, and S9 Fig to S11 Fig), but their timing differs across realisations, and so the average profiles shown in Fig 7A and 7B smooth the saltations. The very first saltation is mostly aligned across the individual realisations, and so it is quite prominent in both *K* and $\hat{D}$. Fig 7C shows that the fitness *K* and accumulated mutations $\hat{D}$ are strongly temporally aligned (with Pearson correlation 0.99 over 2,191 data points), suggesting that saltations in transmissibility are produced by accumulated mutations.

We also observe that the fitness and accumulated mutations are slightly higher with increasing the population size, although these changes are much smaller than the population differences. All three population sets produced saltations in fitness and accumulated mutations. However, in smaller populations (i.e., 1.7 million), profiles of these two measures are not only slightly lower but also somewhat less abrupt, relative to the profiles produced in larger populations (8 million and 25.4 million).

**Fig 8:**
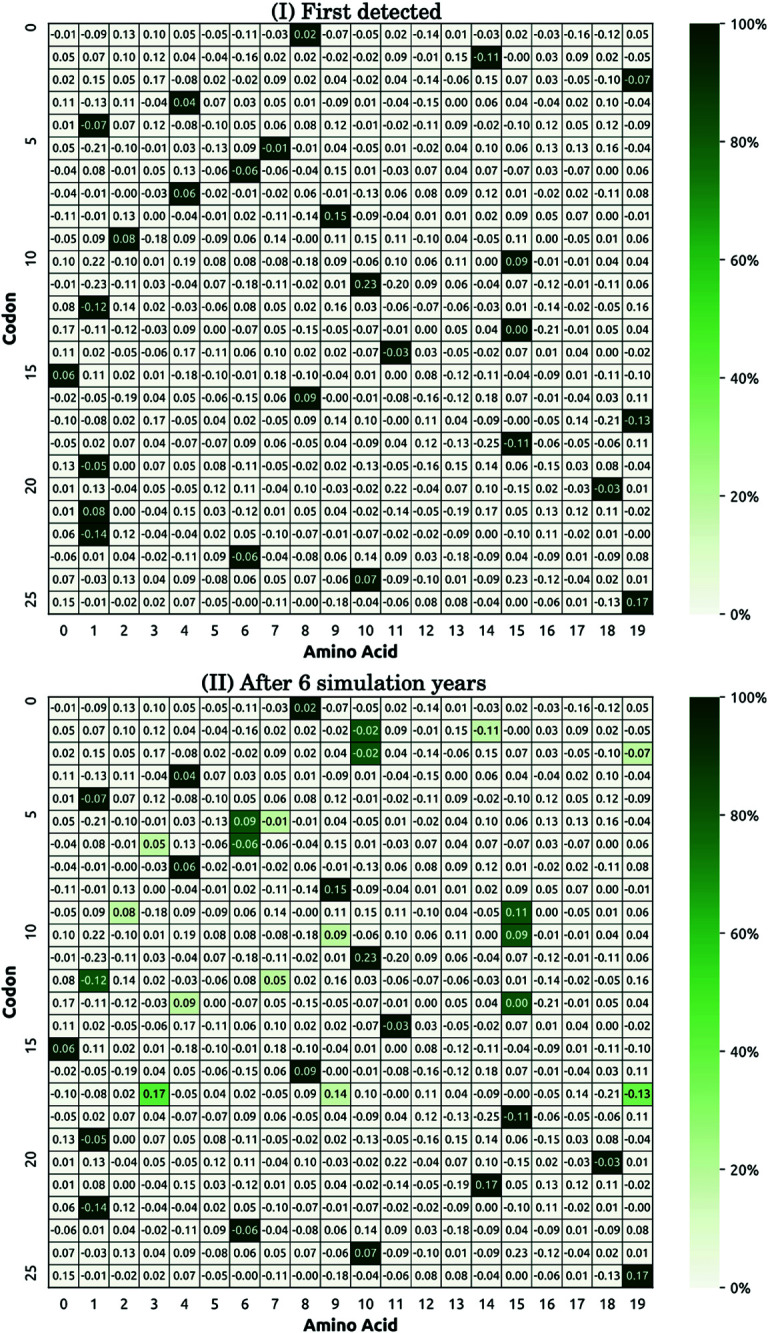
Snapshots of amino acids – codons histograms at different simulation time steps, obtained from a single realisation using the population of 1.7 million agents (Capability 2(ii)). Amino acids in the first 26 codons (i.e., the first 78 nucleotides) are shown here for simplicity. Note that the numerical fitness contributions are generated following distributions specified in S1 Text (Contribution to fitness), prior to each simulation run. (I) The histogram produced for the first day when cases were detected, and (II) the evolved histogram computed from 500 randomly selected genomes after 6 simulation years. The colour intensity represents the frequency of a given amino acid–codon combination in the sampled genome(s). The value shown in each histogram cell represents the pre-defined amino acid contribution to fitness.

To explain the punctuated increase in fitness, we traced changes in the distribution of fitness contributions across the simulated genome (Capability 2(ii)) by comparing the relative frequencies of amino acids in simulated ancestral genome (Fig 8 (I)) and the evolved distributions in circulating genomes after six simulation years (Fig 8 (II)). In simulated dynamics, we observed a clear shift towards amino acids with positive fitness contributions, in accordance with empirical observations (Fig 2C and 2D). This observation indicates that the viral mutations are subject to selective pressure transitioning to a higher point in the viral fitness landscape. Section Materials and methods and S1 Text (Contribution to fitness) provide a detailed description of the fitness contribution method.

**Fig 9:**
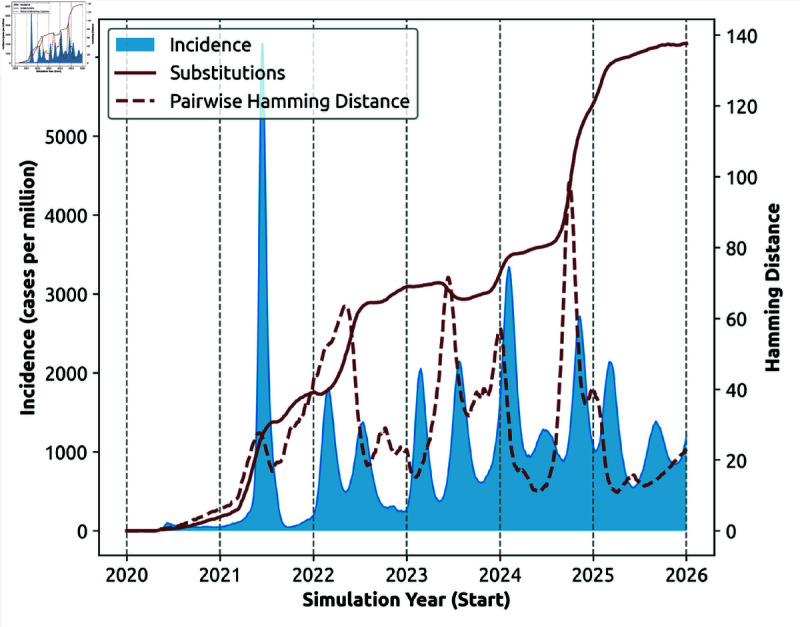
Temporal alignment of phylodynamics and epidemiological dynamics (Capability 3i), simulated for a 1.7 million population over a 6-year period. Alignment of epidemic incidence (shaded blue area), accumulated mutations ($\hat{D}$, solid brown line), and genomic diversity ($\overset{―}{D}$, dashed brown line). Profiles are shown for a single realisation.

**Capability 3.** In pursuing our final objective, we explore the emergence and dominance of variants of concern in the simulated dynamics. We begin by examining whether the simulated phylodynamic characteristics are temporally aligned with the disease incidence. We note that saltations in the accumulated mutations $\hat{D}$ can be matched by peaks in the genomic diversity $\overset{―}{D}$, as shown by Fig 9. These diversity peaks indicate a rise of a new variant (i.e., increase in diversity), followed by its dominance (i.e., decrease in diversity) until yet another variant emerges. In turn, these abrupt changes correspond to incidence peaks, that is, there is a notable synchrony between dynamics of the incidence and the genomic diversity $\overset{―}{D}$. At the same time, not all incidence peaks can be explained by the changes in genomic diversity or the saltations in fitness.

These observations are well aligned with the empirical data for the first four COVID-19 pandemic years. In particular, Fig 3A suggests that, while the accumulated mutations monotonically increase, the genomic diversity fluctuates along a volatile pattern with periods of drift (i.e., steady increase) followed by rapid increases and sudden collapses in the pairwise Hamming distance. Our simulation produced a similar alignment between the growing accumulated mutations $\hat{D}$ and the fluctuating genomic diversity $\overset{―}{D}$, as illustrated by Fig 9. We note that Fig 9 illustrates the genomic diversity dynamics based on one simulation realisation (for 1.7 million agents). S13 Fig to S15 Fig show simulation results across multiple realisations and different population sizes.

**Fig 10:**
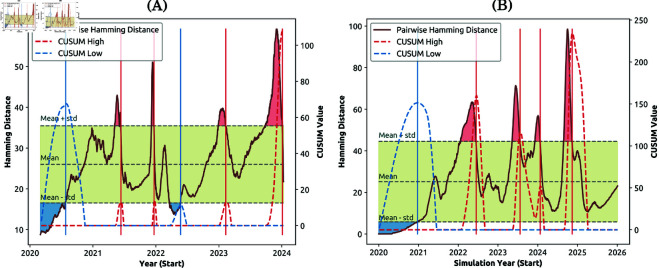
Detection of emerging variants (Capability 3 ii) by applying CUSUM to the genomic diversity quantified by pairwise Hamming distance ($\overset{―}{D}$). (A) The empirical genomic diversity based on Nextstrain datasets [15]. (B) Simulated genomic diversity in a population of 1.7 million. Vertical lines represent variants detected using CUSUM high (red) and CUSUM low (blue). The yellow shaded area shows the control range around the mean $\overset{―}{D}$ within one standard deviation. Simulated profile in (B) is shown for a single realisation.

Aiming to demonstrate Capability 3 (ii), we quantified the emergence and dominance of variants by analysing the genomic diversity dynamics with a statistical technique based on deviations from Cumulative Sum (CUSUM), and visualising the phylogenetic tree (see Section Materials and methods). The analysis was applied to both empirical and simulated data. For empirical data, we detected six notable deviations (Fig 10A), identifying six emergent and dominant variants between 2020 and 2024. For the simulated dynamics, five deviations were detected between 2020 and 2026 (Fig 10B).

**Fig 11:**
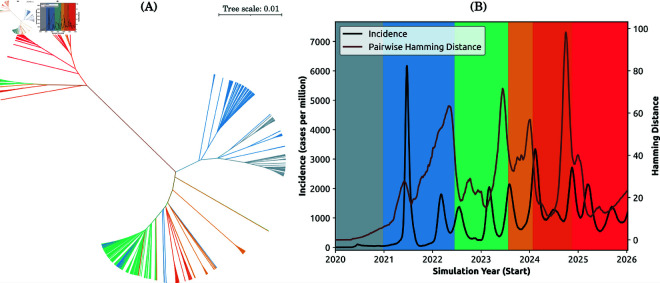
Evaluation of the emergence and dominance of variants (Capability 3 ii). (A) Phylogenetic tree constructed using BEAST [37,38] and iTol [39], depicting the genomes of the most transmissible strains sampled every half-day. A total number of 4,394 genomes are plotted. (B) Alignment of simulated incidence (solid black line), the genomic diversity ($\overset{―}{D}$, solid brown line), and the detected variants identified in Fig 10 (B). Both (A) and (B) are colour-coded according to the day ranges determined by the CUSUM peak detection in Fig 10.

By aligning the genomic diversity with the incidence dynamics (Fig 11B), we observe that distinct transitions between the variants which emerged during simulation coincide with sharp changes in the corresponding genomic diversity. We also note stationarity of the genomic diversity in smaller populations, although the time series become less stationary in larger populations (see S1 Text: Stationarity of genomic diversity). It is well known that the spatiotemporal synchrony of disease spread between communities is correlated to the population size of the communities [19]. In our study, the smaller population (1.7 million) represents the state of South Australia, with its capital (Adelaide) comprising a significant fraction of this population (see S1 Text: Artificial agent-based population). Consequently, the population size of local government areas is relatively homogeneous and the population is more well-mixed. Our conjecture is, therefore, that a newly emerged and more transmissible strain tends to dominate more easily, reducing the average genomic diversity. This yields a more homogeneous immune response within a smaller population, dampening the incidence waves (see discussion of Capability 1 above).

Finally, we constructed a phylogenetic tree using simulation results. The tree is shown in Fig 11A, with the branches colour-coded by the variants identified using CUSUM, corresponding to Fig 10B. Notably, the phylogenetic tree reveals that (1) the new variants emerge at a distance from the ancestral strain, while branching away from more basal lineages, and (2) the variants detected in later years are closer to each other. This branching structure could be explained by the time it takes a new variant to accumulate novel antigenic features and increase its fitness due to these antigenic advantages. These observations are consistent with the branching of the phylogenetic tree constructed using empirical data (Fig 4B).

Overall, these simulation results, aligned with the empirical observations, imply that a rapid punctuated evolution with prominent saltations in transmissibility is driven by accumulation of fitness-increasing mutations relative to the ancestral strain, aided by persistent, chronic infections. The simulated phylodynamics produces a fluctuating genomic diversity, associated with the emergence and dominance of variants of concern, at least during a mid-term pathogen evolution.

## Discussion

In this study, we identified key requirements for a new class of multi-scale pandemic models. The three considered dynamics included: (1) pathogen evolution, punctuated by the emergence of new pathogen variants, (2) human interactions within heterogeneous populations, and (3) public health interventions aimed at controlling disease transmission. We described specific modelling capabilities, and developed a computational framework, implemented in a comprehensive simulator — PhASE TraCE — which meets these objectives. PhASE TraCE is capable of modelling the spread of infectious diseases while accounting for the evolutionary trajectory of pathogens across diverse demographics. We validated the framework with a COVID-19 case study, by calibrating PhASE TraCE to the phylodynamic and epidemiological characteristics of SARS-CoV-2, and simulating the corresponding immuno-epidemiological and phylodynamic patterns across heterogeneous population settings, scaling the demographics to different sizes. We then applied different phylodynamic measures to analyse the simulated dynamics to detect emerging and dominating pathogen variants, aligning the outcomes with empirical observations. In general, while the epidemiological layer can be calibrated and validated relatively quickly, the phylogenetic and immunological layers require a greater effort to calibrate using information collected over a longer time period. However, once this process is complete, this model can be used not only for retrospective phylodynamic analysis, but also for an investigation of future longer-term evolutionary and epidemiological trends and risks. PhASE TraCE can also be calibrated to pandemic scenarios related to other respiratory diseases with known phylodynamic and epidemiological characteristics. In cases where phylodynamic and epidemiological evidence is lacking, PhASE TraCE can be used to generate a suitable model and investigate various “what-if” scenarios under different phylodynamic hypotheses. However, scenarios related to non-respiratory diseases (e.g., foodborne epidemics, vector-borne diseases) warrant further research.

In particular, PhASE TraCE reproduced recurrent incidence waves with salient initial peaks and a transition to endemicity (Capability 1). These observations showed that, given the waning immunity and reduced vaccine efficacy against emerging variants, the adopted NPIs and vaccination roll-outs would not eliminate the spread completely. Importantly, the simulated phylodynamics produced a rapid punctuated evolution, and this was explained in terms of the accumulation of fitness-increasing mutations within chronically infected hosts. This increase was further confirmed by a clear shift in the simulated distribution of amino acids towards fitness-increasing mutations, appearing due to selective pressure (Capability 2). Finally, we related the emergence and dominance of variants of concern to prominent changes in genomic diversity. This analysis was supported by the visualisation of the phylogenetic tree and relevant stationarity tests (Capability 3).

PhASE TraCE is designed to support the testing of different hypotheses by simulating counterfactual modelling scenarios. For example, chronic infections have been hypothesised to contribute to the rapid punctuated evolution of SARS-CoV-2, being often associated with accelerated substitution rates, and a higher genetic diversity and selective pressure [32]. We applied PhASE TraCE to investigate the potential role of chronic infections on the pathogen evolution by comparing resultant phylodynamics with and without chronically infected hosts (see S1 Text: Chronic infections).

Computationally, PhASE TraCE involves a nested stochastic simulation, where the (micro-scale) within-host pathogen evolution is simulated in every artificial agent, while the disease transmission is simulated based on the (meso-scale) agent interactions within a heterogeneous population. Finally, the (macro-scale) public health interventions, such as NPIs and vaccination, are simulated at the population level, constraining the actions of individual agents. The addition of immunological and phylogenetic layers significantly extended the capabilities of PhASE TraCE relative to state-of-the-art pandemic ABMs, although at the cost of higher computational complexity and the need to integrate fragmented data inputs. Nevertheless, the approach retains computational tractability and scalability (see S1 Text: Computational complexity and implementation).

We acknowledge several limitations of the current study. We assumed that at any given time, each infected host can only carry a single strain, without exploring the possibility of co-infection (i.e., a simultaneous infection by multiple strains). In our case study, we simplified the genome representation, partitioning it into spike and non-spike regions, with an overlapping region representing epitopes containing genetic information relevant to triggering immune responses (i.e., transmissibility and antigenicity). We assigned fitness based on a linear combination of contributions from individual amino acids, without modelling transcription. This precludes nonlinear contributions, which may be more relevant as a large number of mutations accumulate. Our design also does not accommodate biologically important fitness effects due to RNA secondary structure, codon usage bias and GC content[40], gene regulation, and ribosomal frameshifts. A potential extension of the model could investigate a more comprehensive definition of fitness.

Our mutation model assumes that only point mutations occur, with no structural changes such as insertions, deletions and recombination. We also adopt a simple Jukes-Cantor substitution model with equal rates between nucleotides. An extended model that incorporated more nuanced fitness and transmissibility effects would need to revisit these simplifying assumptions.

We implemented within-host selective pressure by generating numerous mutant strains and selecting a strain among the most transmissible of these candidates. This allowed us to investigate whether a bias towards higher transmissibility in a subset of hosts was sufficient to generate the observed lineage dynamics. In reality, the host environment selects for factors other than transmissibility, particularly in chronically infected hosts (who might be immunocompromised and receiving antiviral or antibody therapy). In our model, each infected host only has a single viral genome associated with them at any given simulation time step, and the only “functions” derivable from our synthetic genome representation are transmissibility and antigenic distance. A possible extension might model virulence and directly simulate competition between a population of viral lineages in each host. This would require considerable work, calibration, and computational resources.

We made a simplifying assumption that the imported infections carry a strain with the highest transmissibility among the strains circulating during the preceding simulation month. This component could be made stochastic by considering multiple imported strains, chosen in proportion to their incidence.

For computational efficiency, we modelled phylodynamics of a pandemic pathogen in a population of up to 25.4 million, whereas the relevant population size is much larger, approaching the size of the world population. However, our results demonstrated that the key simulation outcomes (such as substitution rate, fitness level, accumulated mutations, and genomic diversity) scaled sublinearly, indicating convergence as the population size grows (Fig 7). Secondly, we reduced the genome length to a tenth of the SARS-CoV-2 genome length. This may partially explain why the genomic diversity becomes less stationary in larger populations, compared to stationarity of the genomic diversity computed using empirical data (see S1 Text: Stationarity of genomic diversity).

Furthermore, we did not exhaustively explore the impact of different weight tables, defining the fitness contribution of amino acids, on the genomic diversity over time and its resultant (non-)stationarity. In addition, the employed stationarity test (ADF) is known to be sensitive to the number of included lags in the time series. These limitations may be overcome in future studies, further enhancing the modelling scope and range of applicability of PhASE TraCE.

## Materials and methods

### Multi-layer architecture of PhASE TraCE

**Fig 12:**
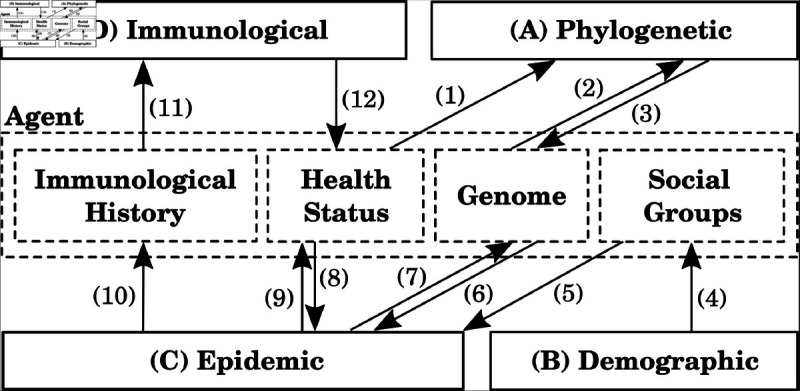
Architecture of PhASE TraCE. Four processing layers (A–D) and twelve data flows update four core attributes of the *Agent* objects.

PhASE TraCE is a large-scale stochastic simulator developed to model the mid- to long-term phylodynamics of pathogens within a heterogeneous population of agents. Computationally, each agent in PhASE TraCE is represented as an object with multiple attributes which can be modified by processing layers: (A) Phylogenetic, (B) Demographic, (C) Epidemic, and (D) Immunological, as illustrated in Fig 12.

Each agent has four core attributes: (i) immunological history which records past infections and vaccinations, as well as the associated time stamps of these events; (ii) health status, which tracks the agent’s current health state (Susceptible, Asymptomatically Infectious, Symptomatically Infectious, or Recovered), infected host category (typical infected or chronically infected), and the current immunity against circulating variants; (iii) genome profile of the variant carried by the agent (if infected); and (iv) the social groups, which indicate the social contexts where interactions occur.

The flows between processing layers and agent attributes are directional, as illustrated in Fig 12: the flows from processing layers to agent attributes modify the attributes, while the flows from agent attributes to processing layers influence the processing layer, as described below.

**(A) Phylogenetic**: this layer simulates mutations and selective pressure on the genome carried by an infected agent, as detailed in S1 Text (Phylogenetic model). Once the agent becomes infectious, the Phylogenetic layer receives input on the agent’s health status, including the host type category via (1), and the genome profile derived from the infection source via (2). The mutated genome is then saved in the agent’s genome attribute via (3).

**(B) Demographic**: prior to the simulation, this layer generates a heterogeneous artificial population of a specified size, based on 2021 Australian Census data [41]. The generated demographics are used in constructing the social groups via (4), determining each agent’s social mixing contexts which constrain agent interactions. These contexts include (a) residential contexts, such as households, household clusters, and statistical areas (SAs) at various resolution levels; and (b) studying/workplace environments, such as schools or working groups, depending on the agent’s age group. Details on the Demographic layer and artificial population generation are provided in S1 Text (Artificial agent-based population).

**(C) Epidemic**: this layer models disease transmission and control, detailed in S1 Text (Multi-strain transmission model). The infection transmission is modelled stochastically, being affected by three agent attributes: social groups which constrain interactions between susceptible and infectious agents, via (5); health status which comprises the agent’s health state and immunity level, given previous immunological events, via (8); and genome profile which provides a representation of the pathogen infecting the agent, via (6). Having simulated a transmission of infection between two agents, the epidemic layer updates three agent attributes: health status, modifying the agent’s health state, via (9); genome profile, by using the representation of the pathogen variant carried by the source of infection, via (7); and immunological history once the infected agent recovers, via (10).

**(D) Immunological**: this layer reads from an agent’s immunological history of past infections and vaccinations, via (11); and updates the immunity level as part of the agent’s health status, via (12). S1 Text (Vaccination) provides a detailed description of the vaccination component. The immunity levels are also affected by a non-linear accumulation over multiple immunity-boosting events (i.e., compound immunity) and the associated waning effects, detailed in S1 Text (Immunological layer) and S1 Text (Compound immunity and waning effects).

An efficient implementation of this multi-layer architecture requires a resolution of several computational challenges, given the demanding simulation timeframe (over 6 years) and memory-intensive tasks associated with storing evolving agent attributes, particularly in large populations. To address these challenges, we employed multi-threading processing that computes multiple attributes in parallel in each *Agent* object, with the attributes configured independently from each other to achieve concurrency. In doing so, we significantly increased the computational efficiency by reducing both simulation time and associated computational costs. S1 Text (Computational complexity and implementation) details an analysis of the performance, scalability, and computational resources of PhASE TraCE.

### Multi-scale phylodynamic simulation

This section describes processing layers (A), (C) and (D), highlighting their key dynamic relationships. Since the demographic characteristics of agents, including their social groups, are not updated during simulation, there is no dynamic modelling involved in layer (B), and this layer is described in S1 Text (Artificial agent-based population).

#### Phylogenetic layer (A).

The phylogenetic layer models the within-host evolution (mutation and selection), using pathogen genomes associated with each infected agent, and computes the fitness of circulating variants. An artificial genome contains 3,090 nucleotides (or 1,030 codons) which are associated with 20 known types of amino acids. The genome structure is partitioned into the spike region (100 codons) and the non-spike region (930 codons), with an overlapping region accounting for epitopes (75 codons, with 45 of these located in the spike region and 30 in the non-spike region). S1 Text (Genome structure) provides a detailed description of the genome structure, followed by S1 Text (Mutations) describing point mutations.

Pathogen fitness is defined in terms of its transmissibility, proportional to the corresponding basic or effective reproductive number (*R*_0_ or $R_{\text{eff}}$). To quantify the individual fitness contributions of amino acids at each codon position, we employ a weight table, specifying weights *a*_*i*,*j*_ of *N*_*A*_ = 20 types of amino acids across N=1,030 codon positions, as detailed in S1 Text (Contribution to fitness). The overall fitness *K* of a strain with genome *s* is determined as the sum of these individual contributions:

$$K(s)=\sum_{i=0}^{N-1}a_{i,s(i)}$$ (1)

where *a*_*i*,*s*(*i*)_ is the fitness contribution of amino acid j=s(i) located at codon position *i*, with $j\in [0,N_{A}-1]$.

To re-iterate, the phylogenetic model distinguishes between two infected host categories: typical infected hosts and chronically infected hosts (S1 Text: Infected host categories). Consequently, there are different selective pressures driving the within-host evolution of pathogens in these categories (S1 Text: Intra-host (within-host) selective pressure). At each mutation step, we generate multiple candidate sequences, rank them by transmissibility, and focus on the group of top candidates. The size of this group indicates the strength of selective pressure. For example, selecting from a smaller group of top candidates implies a stronger selective pressure, while selecting from a larger group of top candidates implies a weaker selective pressure. When simulating selective pressure for chronically infected hosts, the size of the top group is assumed to be smaller, indicating a stronger selective pressure, compared to the selective pressure for typically infected hosts. We elaborate on this distinction and the limitation of these assumptions in Section Discussion.

We distinguish between mutation and substitution rates. While the mutation rate refers to the frequency of new mutations arising in a genome per unit of time, the substitution rate is the rate at which these new mutations are retained over time within the population [32,42,43]. In our case study, the mutation rate is a key input parameter, whereas the average substitution rate emerges as a simulation outcome. We use the attained substitution rate to calibrate the model, by matching the regression coefficient observed in empirical observations, illustrated in Fig 2B.

#### Epidemic layer (C).

At the start of simulation, pathogens with the ancestral genome are “seeded” by infecting agents residing around international airports. Every month, the simulation updates the genomes seeded around airports, selecting the variant with the highest transmissibility detected during the preceding month.

As described in S1 Text (Multi-strain transmission model), disease is transmitted among agents that interact across different social contexts. The transmission process is simulated in discrete half-day time steps: “daytime” cycles during which agents interact in workplaces or educational settings (e.g., class, grade, school), and “nighttime” cycles during which agents interact in residential settings (e.g., household, household cluster, neighbourhood, and community). Each agent progresses through several health states: Susceptible, Infectious (asymptomatic or symptomatic), and Recovered, following the natural history of the disease.

At simulation cycle *n*, the infection probability *p*_*i*_(*n*) for a susceptible agent *i* is determined across all its social contexts $g\in G_{i}$ (see S1 Text: Susceptible-infectious transition):

$$p_{i}(n)=1-\prod_{g\in G_{i}(n)}\;\prod_{j\in A_{g}\backslash \{i\}}\left(1-p_{j\to i}(n,g)\right)$$ (2)

where *G*_*i*_(*n*) denotes the set of all social contexts *g* that agent *i* interacts with during the time cycle *n*, $A_{g}\backslash \{i\}$ denotes the set of agents in *g* (excluding agent *i*), and $p_{j\to i}(n,g)$ denotes the probability of infection transmission from infectious agent *j* to susceptible agent *i* within their social context $g\in G_{i}(n)$. The probability $p_{j\to i}(n,g)$ is defined as follows:

$$p_{j\to i}(n,g)=K(s_{j})\;f_{j}(n-n_{j})\;q_{j\to i}(g)$$ (3)

where $q_{j\to i}(g)$ is the age-dependent interaction probability within *g* (see S1 Text: Susceptible-infectious transition); *n*_*j*_ denotes the infection onset time for agent *j*; the agent-specific function *f*_*j*_(*n* − *n*_*j*_) is the natural history of the disease, reflecting the infectivity of agent *j* as its infection progresses (see S1 Text: Infectious-recovered and recovered-susceptible transitions); and *K*(*s*_*j*_) represents the transmissibility of pathogen variant *s*_*j*_, proportional to the corresponding basic reproductive number *R*_0_ or effective reproductive number $R_{\text{eff}}$, i.e., *K*(*s*_*j*_) is the fitness of genome *s*_*j*_ carried by agent *j*, as defined by Eq 1.

Once susceptible agent *i* becomes infected, it is possible to assign (i.e., identify) a specific infectious agent as the source of infection. This is simulated by weighted random sampling of an infection source from all potential infectious agents *j* across all social contexts $g\in G_{i}(n)$ in which agent *i* interacted during this cycle. Then the pathogen genome profile *s*_*j*_ (carried by the identified infection source agent *j*) is copied to agent *i* (see S1 Text: Susceptible-infectious transition).

Various interventions may change the infection probabilities across social contexts. Modelling non-pharmaceutical interventions (NPIs) is described in Section Non-pharmaceutical interventions.

#### Immunological layer (D).

The immunological layer simulates a vaccination rollout, including vaccination coverage, schedule and rates, given vaccine efficacy. Furthermore, the immuno-epidemiological model quantifies the agent immunity resulting from multiple immunity-boosting events (i.e., vaccinations and infections), as described in S1 Text (Vaccination). In this work, we broaden the concept of “hybrid immunity” to “compound immunity”, in order to capture non-linear immunity accumulation over various combinations of prior vaccinations and infections. Compound immunity may result from one or multiple vaccinations, one or multiple past infections, or a combination of both vaccination(s) and past infection(s) [28].

We decompose the compound immunity via three separate sub-components, quantifying reductions of different risks: susceptibility, symptomatic infection, and forward transmission. For example, the compound immunity against symptomatic infection, denoted $M_{i}^{\text{c}}$, for susceptible agent *i* interacting with infectious agent *x*, is defined as follows:

$$M_{i}^{\text{c}}(n,H_{i},s_{x})=\text{min}\left(\sqrt{\sum_{r\in H_{i}}{\left[m_{i}^{\text{c}}(n,r,s_{x})\right]}^{2}},1\right)$$ (4)

where $m_{i}^{\text{c}}(n,r,s_{x})$ is the immunity against symptomatic infection induced by past immunological event *r* (vaccination or infection); *H*_*i*_ is the immunological history formed by past records *r* up to cycle *n*; and *s*_*x*_ is the genome carried by infectious agent *x*. In addition, the compound immunity wanes over time and depends on the genetic distance (see Eq 12 in S1 Text: Compound immunity against symptomatic infection). S1 Text (Compound immunity and waning effects) provides more details accounting for different vaccine efficacy components contributing to the compound immunity, and its effects on infection probabilities.

### Phylodynamic measures

#### Hamming distance.

In this study, we used Hamming distance as the primary measure to count nucleotide differences between two genomes [44]. The average reference Hamming distance between evolved genomes and the reference genome (i.e., ancestral genome), denoted $\hat{D}$, is used to account for the mutations accumulated during the simulation timeframe, while the average pairwise Hamming distance among evolved genomes, denoted $\overset{―}{D}$, is used to quantify the genomic diversity. The computation of these two measures is described below.

To quantify the accumulated mutations $\hat{D}$, for each simulated day *n*:

- Select all genome profiles obtained within a one-week forward window starting on day *n*.
- For each of the profiles, record the number of differences against the reference genome (i.e., ancestral genome, NCBI GenBank accession number MN908947 [18,45]), producing the reference Hamming distance.
- Compute the average reference Hamming distance across all circulating variants.

To quantify the genomic diversity $\overset{―}{D}$, for each simulated day *n*:

- Randomly select 10,000 pairs of profiles obtained within a one-week forward window starting on day *n*.
- For each pair of genomes, record the number of differences between them as the pairwise Hamming distance.
- Compute the average pairwise Hamming distance of all pairs.

Figs 2 and 3 trace the reference and pairwise Hamming distances for actual SARS-CoV-2 sequence data [15].

In order to trace Hamming distances for the simulated dynamics we followed slightly altered workflows, without applying the weekly windows for genome selection. When dealing with actual sequence data, these windows were needed to filter out sampling inconsistencies. Simulated data include pathogen genomes from all detected hosts, sampled on each simulation day, thus reducing sampling inconsistencies. The reference Hamming distance $\hat{D}$ was computed against the simulated ancestral strain, constructed for each realisation (as described in Section Materials and methods). Figs 7, 9 – 11 trace the reference and pairwise Hamming distances for the simulated phylodynamics.

#### Statistical stationarity tests.

To examine stationarity of the pairwise Hamming distance, we performed statistical stationarity tests, specifically Augmented Dickey-Fuller (ADF) test and one-sided Cumulative Sum (CUSUM) analysis. This allowed us to identify saltations as punctuated changes in the pairwise Hamming distance, which have been found to be closely related to the emergence and dominance of pathogen variants [13].

**ADF test.** An ADF test detects non-stationarity in a time series [46]. We use the following null and alternative hypotheses to determine stationarity:

*H*_0_: Pairwise Hamming distance is non-stationary.
*H*_1_: Pairwise Hamming distance is stationary.

We computed p-value from the ADF test and compared it against a chosen significance level (i.e., 0.05), with p-value smaller than the significance level rejecting *H*_0_ and confirming stationarity. Results of ADF are shown in S1 Text (Stationarity of genomic diversity) and S17 Fig.

**CUSUM.** A one-sided Cumulative Sum (CUSUM) control chart [47,48] can identify *anomalies* in the observed time series. In this study, we applied CUSUM on pairwise Hamming distance as shown in Fig 10.

Let *W*(*n*) be the pairwise Hamming distance on day *n*, with the mean μ and standard deviation σ. We converted *W*(*n*) into *high* CUSUM $\left({\text{S}}^{\text{X}}\right)$ and *low* CUSUM $\left({\text{S}}^{\text{Y}}\right)$, as follows:

$$S^{X}(n+1)=\text{max}\left(0,S^{X}(n)+W(n+1)-\mu -\sigma \right)$$ (5)

$$S^{Y}(n+1)=\text{max}\left(0,S^{Y}(n)-W(n+1)+\mu -\sigma \right)$$ (6)

where $S^{X}(0)=S^{Y}(0)=0$.

The high and low CUSUM values, ${\text{S}}^{\text{X}}$ and ${\text{S}}^{\text{Y}}$, are traced in Fig 10. We then applied a positive peak detection in ${\text{S}}^{\text{X}}$ and ${\text{S}}^{\text{Y}}$ (i.e., considering CUSUM value greater than 0), to detect the anomalies. Specifically, the peaks identified in ${\text{S}}^{\text{X}}$ indicate the emergence of a more transmissible variant, whereas the peaks identified in ${\text{S}}^{\text{Y}}$ indicate the dominance of a variant within the population. The number of dominant variants is equivalent to the total number of peaks detected in both ${\text{S}}^{\text{X}}$ and ${\text{S}}^{\text{Y}}$.

We note that CUSUM is a simplified approach and can only identify one variant of concern during a defined period. In other words, it cannot trace the frequency of multiple co-circulating variants and the associated transitions.

## Supporting information

S1 Table The micro-distancing parameters (interaction strengths) for the considered NPIs. The micro-duration of CI is limited by the disease progression in the affected agent *i*, *D*(*i*). Interaction strengths for CI are set to be significantly lower for chronically infected hosts. CI: Case Isolation; HQ: Home Quarantine; SC: School Closure; and SD: Social Distancing.
(TIF)

S2 Table The macro-distancing parametrisation (population fractions) for the considered NPIs over the 6-year simulation period for SARS-CoV-2. The CI-compliant population fraction is lower for typically infected hosts than for chronically infected hosts. Students/teachers are assumed to fully comply with SC, while parents of school-aged children have a reduced compliance level. CI: Case Isolation; HQ: Home Quarantine; SC: School Closure; and SD: Social Distancing.
(TIF)

S3 Table Simulation parameters for compound immunity. (TIF)

S4 Table Phylogenetic parameters used in the SARS-CoV-2 case study. (TIF)

S1 Fig Simulated genome structure consisting of 3,090 nucleotides (nts), equivalent to 1,030 codons. The genome is partitioned into spike (orange) and non-spike (blue) regions. Additionally, the nucleotides can be grouped according to functions: (i) regions consisting of 1,000 codons (100 from spike region and 900 from non-spike region), contributing to the pathogen fitness and the resultant transmissibility; and (ii) regions consisting of 75 codons (45 from spike region and 30 from non-spike region) contributing to antigenicity (i.e., epitopes). The inset displays examples of grouping nucleotides into codons followed by translation to amino acids. For example, nucleotides 0 1 0 form a codon, which is translated as amino acid 16.
(TIF)

S2 Fig A section of the weight table that quantifies the fitness contributions of 20 amino acids across the genome. Only the first 26 codon positions (vertical axis) are shown for illustrative purposes. Each cell value indicates the potential contribution from each amino acid if present (horizontal axis) at the corresponding codon position (vertical axis). The cell values are sampled from a normal distribution with a mean of 0 (i.e., most random mutations are neutral). Cell colour indicates the magnitude of fitness, with darker colour representing fitness increase and lighter colour representing fitness decrease.
(TIF)

S3 Fig Histogram of infection period of the COVID-19 cases reported by various studies. Datasets were obtained from [31,49], updated as of December 25, 2022.
(TIF)

S4 FigA schematic representation of the natural history describing the SARS-CoV-2 infection progression in an agent.The infectivity initially increases exponentially from the onset of infection, reaching the peak, and subsequently declining linearly to zero until recovery. For each infected host, the duration from the infection onset to the infectivity peak is sampled from a lognormal distribution (A) with parameters μ=1.013 and σ=0.413. The recovery period is sampled from a uniform distribution, ranging from 7 to 11 days (B) for typical infections, or from 60 to 370 days (C) for chronic infections.(TIF)

S5 Fig A simulated mass vaccination roll-out with varying daily vaccination rates (y-axis) and multiple boosting events targeted for COVID-19 (green arrows). The percentage (in blue) within each bar represents the approximate percentage of the vaccinated population at the end of the year.
(TIF)

S6 Fig Vaccine effectiveness against infection reduction over time. Two widely used mRNA vaccines are studied: (A) Pfizer (BNT162b2), and (B) Moderna (mRNA-1273), plotted and fitted using data from [50]. The equation shown in each plot represents the fitted linear regression for each vaccine, estimating a linear reduction in effectiveness from 2.1% to 2.4% per month.
(TIF)

S7 Fig Detected incidence: log scale. (A) Simulated incidence on a log scale in population sets of 1.7 million (red), 8 million (blue), and 25.4 million (green). This figure corresponds to Fig 6A on a linear scale. (B) Worldwide detected incidence [14], measured as new weekly cases per million (solid black line) and new daily cases per million (dashed black line).
(TIF)

S8 Fig Simulated epidemic patterns (Capability 1) shown as mean (solid line) and standard deviation (shaded area). (A) and (C) Population across different health states, including susceptible (blue), asymptomatically infectious (purple), symptomatically infectious (red), and recovered (green) for the populations of 8 million and 25.4 million, respectively. (B) and (D) Population with different immunisation and infection history for the populations of 8 million and 25.4 million, respectively. Numbers in brackets denote the number of immunological (vaccination or infection) records. Individuals with multiple vaccinations or infections (more than 2) are grouped together for simplicity. The mean and average were obtained from approximately 30 realisations.
(TIF)

S9 FigSimulated dynamics of the average transmissibility for a 1.7 million population.Fitness (*K*, olive bars, y-axis on the left), and the accumulated mutations ($\hat{D}$, solid brown line, y-axis on the right) are plotted from 2020 to 2026. Panels (A)–(D) are profiles plotted using four different realisations.(TIF)

S10 FigSimulated dynamics of the average transmissibility for an 8.0 million population.Fitness (*K*, olive bars, y-axis on the left), and the accumulated mutations ($\hat{D}$, solid brown line, y-axis on the right) are plotted from 2020 to 2026. Panels (A)–(D) are profiles plotted using four different realisations.(TIF)

S11 FigSimulated dynamics of the average transmissibility for a 25.4 million population.Fitness (*K*, olive bars, y-axis on the left), and the accumulated mutations ($\hat{D}$, solid brown line, y-axis on the right) are plotted from 2020 to 2026. Panels (A)–(D) are profiles plotted using four different realisations.(TIF)

S12 FigSimulated dynamics of the average transmissibility across different genome regions for a 1.7 million population.(A) Fitness *K* per codon, and (B) accumulated mutations $\hat{D}$ per codon. Five genome regions are plotted: overall genome (red), spike and non-epitope region (blue), spike and epitope (green), non-spike and epitope (purple), and non-spike and non-epitope (pink).(TIF)

S13 FigSimulated dynamics of the average pairwise Hamming distance ($\overset{―}{D}$) between two randomly selected genomes from infected hosts in a population of 1.7 million.Approximately 10,000 pairs of genomes are randomly sampled at each simulation time point from 2020 to 2026 (Capability 2(i)). Opaque red lines represent the ensemble of all realisations and the solid red line shows the dynamics of one realisation only.(TIF)

S14 Fig Simulated dynamics of the average pairwise Hamming distance between two randomly selected genomes from infected hosts in a population of 8.0 million. Approximately 10,000 pairs of genomes are randomly sampled at each simulation time point from 2020 to 2026 (Capability 2(i)). Opaque blue lines represent the ensemble of all realisations and the solid blue line shows the dynamics of one realisation only.
(TIF)

S15 Fig Simulated dynamics of the average pairwise Hamming distance between two randomly selected genomes from infected hosts in a population of 25.4 million. Approximately 10,000 pairs of genomes are randomly sampled at each simulation time point from 2020 to 2026 (Capability 2(i)). Opaque blue lines represent the ensemble of all realisations and the solid blue line shows the dynamics of one realisation only.
(TIF)

S16 FigCounterfactual scenario: chronic infections.Simulated dynamics in scenarios with the fraction of agents susceptible to chronic infections being 0.1% of the entire population (blue curves), and without chronic infections (red curves). The profiles trace detected (A) incidence, (B) average pathogen transmissibility, interpreted as fitness *K*, (C) accumulated mutations ($\hat{D}$), and (D) genomic diversity ($\overset{―}{D}$) in the population of 1.7 million, over six simulation years. The shaded area represents the range of one standard deviation from the mean values. In (A) to (C), the solid lines denote the mean value across 30–50 realisations. In (D), the dashed line denotes the pairwise Hamming distance from one realisation only, given the high variability between realisations.(TIF)

S17 Fig Statistical estimation of stationarity. The stationarity of the time evolution of the average pairwise Hamming distance is computed between two randomly selected genomes. Approximately 10,000 pairs of genome sequences are randomly sampled at each simulation time point from 2020 to 2026 for three simulation scenarios with different population sizes of 1.7 million (A), 8 million (B), and 25.4 million (C) (Capability 3(ii)).
(TIF)

S18 FigStatistical estimation of the stationarity of the average pairwise Hamming distance between two randomly selected genomes simulated with different population sizes.(A) populations of 1.7 million and (B) 8.0 million. Approximately 10,000 pairs of genome sequences are randomly sampled at each simulation time point from 2020 to 2026 for four simulation scenarios by varying amino acid weight table and/or within-host selective pressure (see bar legends). We tested two different fitness weight tables following different normal distributions: (i) *BT*: the baseline setting, where each amino acid contribution in spike and non-spike codon positions is sampled from the normal distributions *N(0, 0.085)* and *N(0, 0.07)*, respectively, and (ii) *AT*: the alternative setting, where each amino acid contribution in spike and non-spike codon positions is sampled from the normal distributions *N(0, 0.0489)* and *N(0, 0.0454)*, respectively. Within-host selective pressure is varied for both typical infected hosts and chronically infected hosts, using notation separated by a vertical bar (e.g., Top99|Top30 represents a setting where selective pressure at top 99 for typically infected hosts (*X=99, M = 100*) and at top 30 for chronically infected hosts (*X=30, M = 100*).(TIF)

S19 FigSensitivity analysis: the fractions of chronic infections.Simulated dynamics of the detected incidence (I-A), average pathogen fitness (I-B), average accumulated mutations $\hat{D}$ (II-A), and average genomic diversity $\overset{―}{D}$ (II-B) (one realisation only), in a population of 1.7 million. These measures are traced while the fraction of individuals susceptible to chronic infection, with strong positive within-host selective pressure, is varied from 0.0 to 0.05 of the total population.(TIF)

S20 FigSensitivity analysis: within-host selective pressure.Simulated dynamics of (A) detected incidence, (B) average pathogen fitness, and (C) average accumulated mutations $\hat{D}$ in a population of 1.7 million. The within-host selective pressure for chronically infected hosts, starting from day 60 of infection, is varied by selecting, in each simulation cycle, the mutated genomes from the top 10% (high selectivity, *X* = 10, *M* = 100) to the top 90% (low selectivity, *X* = 90, *M* = 100) of the ranked list of mutated genome candidates.(TIF)

S21 Fig Reduction of the average processing time (seconds) by utilising more CPUs (between 1 and 8). The simulation covers 365 simulation days for three different population sizes: (A) 230K agents, (B) 1.7M agents, and (C) 8M agents. *Left*: the processing time required to simulate a single simulation day. *Right*: the cumulative processing time required to simulate 365 days. Confidence intervals are shown as shaded areas.
(TIF)

S22 Fig Average cumulative processing time (seconds) for a simulation over 365 days, performed on multiple CPUs in the range between 1 and 8. Population sizes: 230K agents (red), 1.7M agents (green), and 8M agents (blue).
(TIF)

S23 Fig Dependency of the total number of stored immunological records (tens of millions) and amount of memory (RAM, Gigabytes) required for a simulation period of 1,200 days. The immunological records contain vaccination and infection histories of 8 million agents. Each profile represents an individual realisation. All realisations are simulated under identical inputs and settings.
(TIF)

S1 Text Overview of supplementary text. (PDF)
