# Supplementary figures and images for: Multi-scale phylodynamic modelling of rapid punctuated pathogen evolution
Source: PLoS Comput Biol. 2025 Jul 14;21(7):e1013295. doi: 10.1371/journal.pcbi.1013295 (PMC12270310; doi:10.1371/journal.pcbi.1013295)

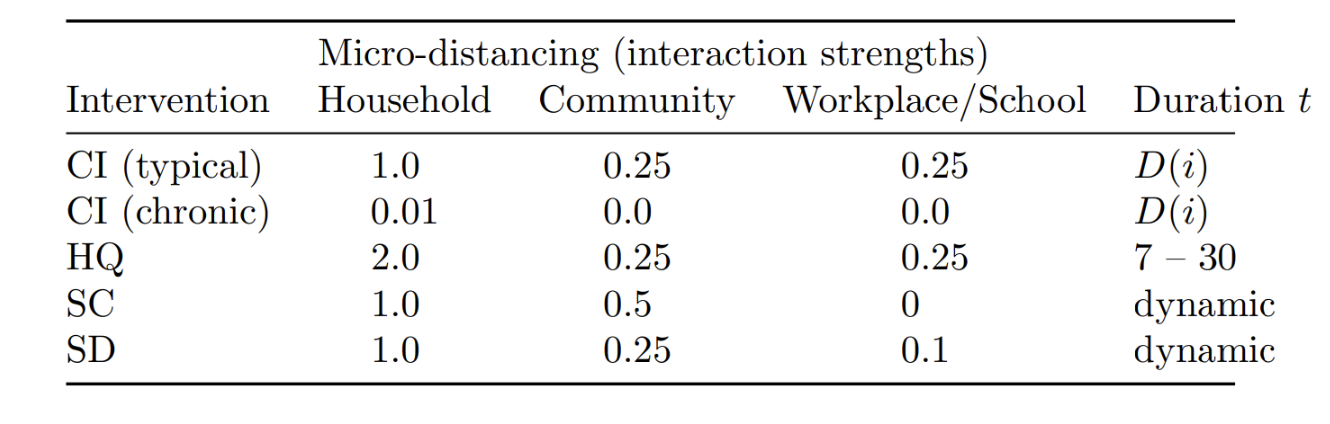

Supplement: S1 Table — The micro-duration of CI is limited by the disease progression in the affected agent i, D(i). Interaction strengths for CI are set to be significantly lower for chronically infected hosts. CI: Case Isolation; HQ: Home Quarantine; SC: School Closure; and SD: Social Distancing. (TIF) [file pcbi.1013295.s001.tif]

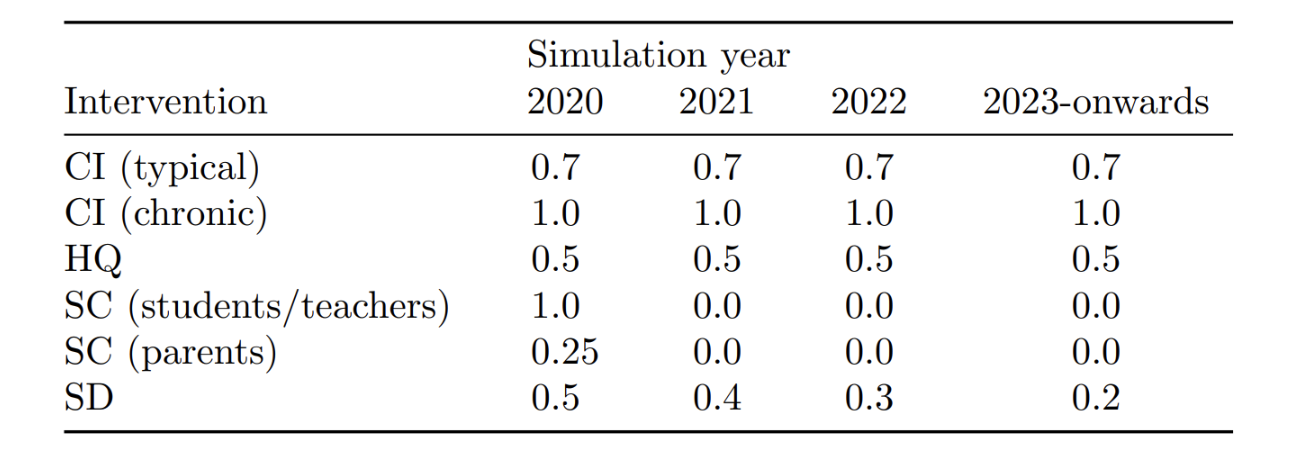

Supplement: S2 Table — The CI-compliant population fraction is lower for typically infected hosts than for chronically infected hosts. Students/teachers are assumed to fully comply with SC, while parents of school-aged children have a reduced compliance level. CI: Case Isolation; HQ: Home Quarantine; SC: School Closure; and SD: Social Distancing. (TIF) [file pcbi.1013295.s002.tif]

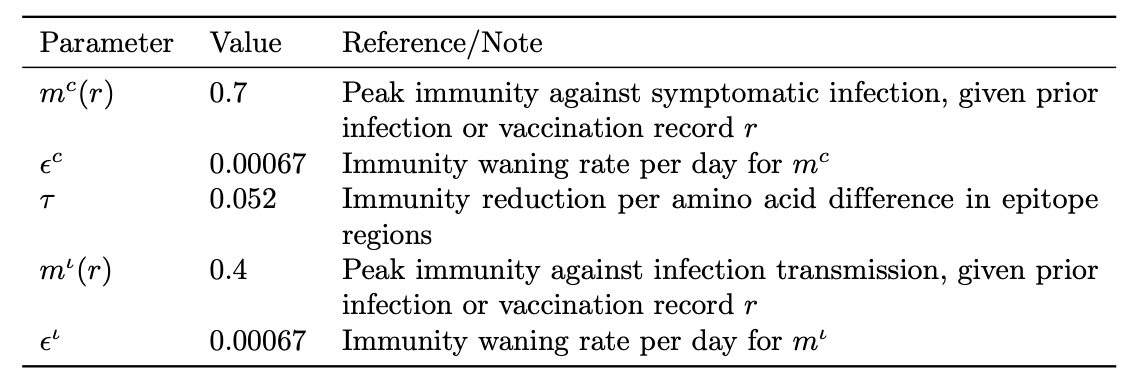

Supplement: S3 Table — (TIF) [file pcbi.1013295.s003.tif]

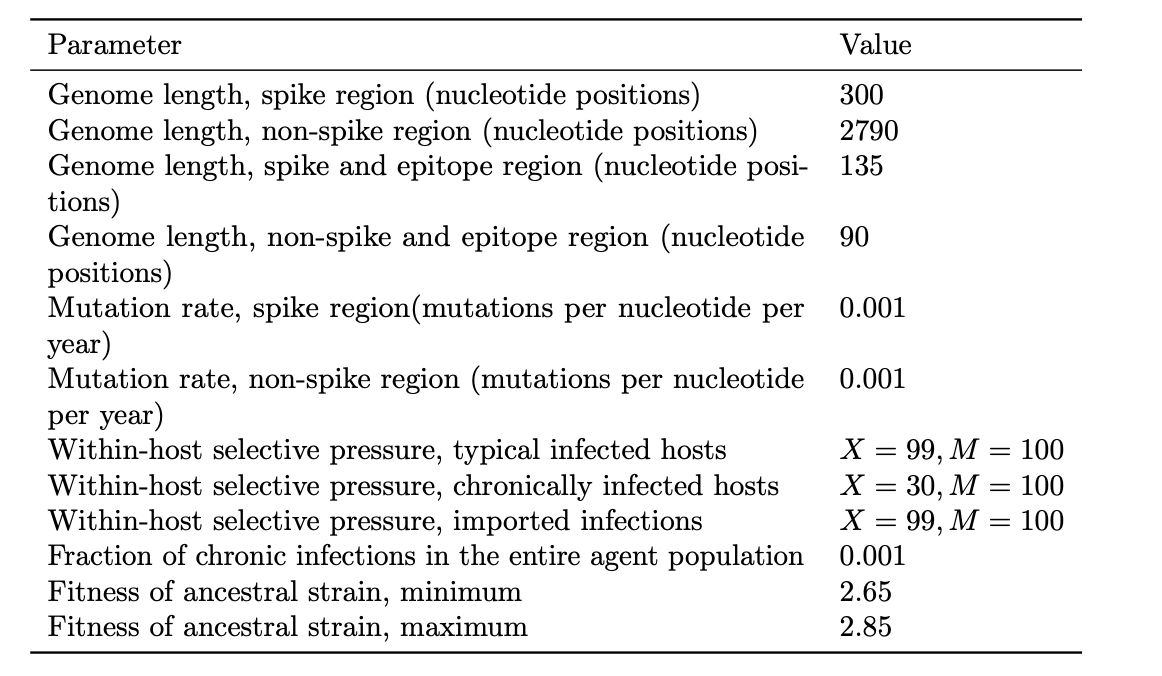

Supplement: S4 Table — (TIF) [file pcbi.1013295.s004.tif]

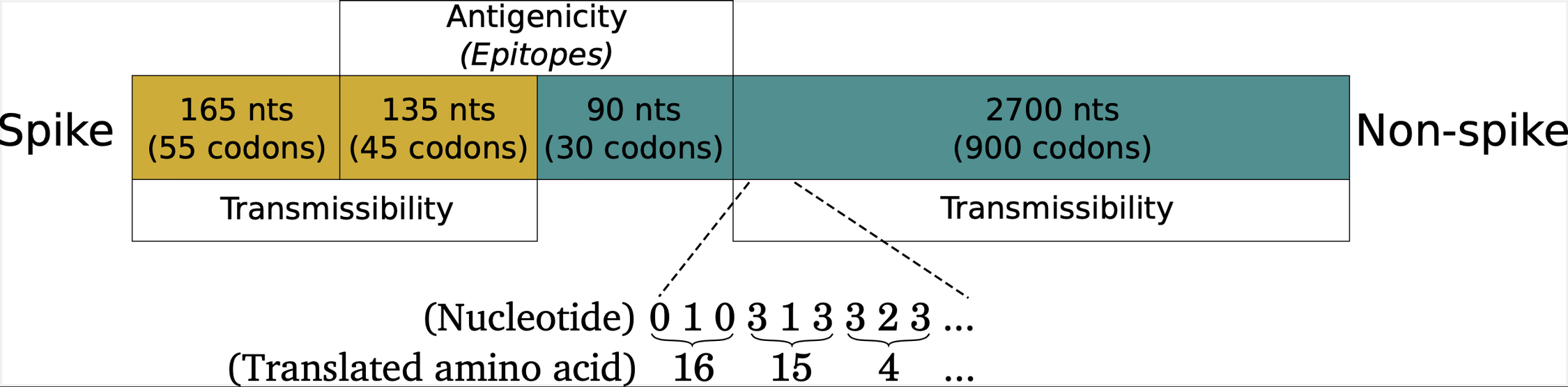

Supplement: S1 Fig — The genome is partitioned into spike (orange) and non-spike (blue) regions. Additionally, the nucleotides can be grouped according to functions: (i) regions consisting of 1,000 codons (100 from spike region and 900 from non-spike region), contributing to the pathogen fitness and the resultant transmissibility; and (ii) regions consisting of 75 codons (45 from spike region and 30 from non-spike region) contributing to antigenicity (i.e., epitopes). The inset displays examples of grouping nucleotides into codons followed by translation to amino acids. For example, nucleotides 0 1 0 form a codon, which is translated as amino acid 16. (TIF) [file pcbi.1013295.s005.tif]

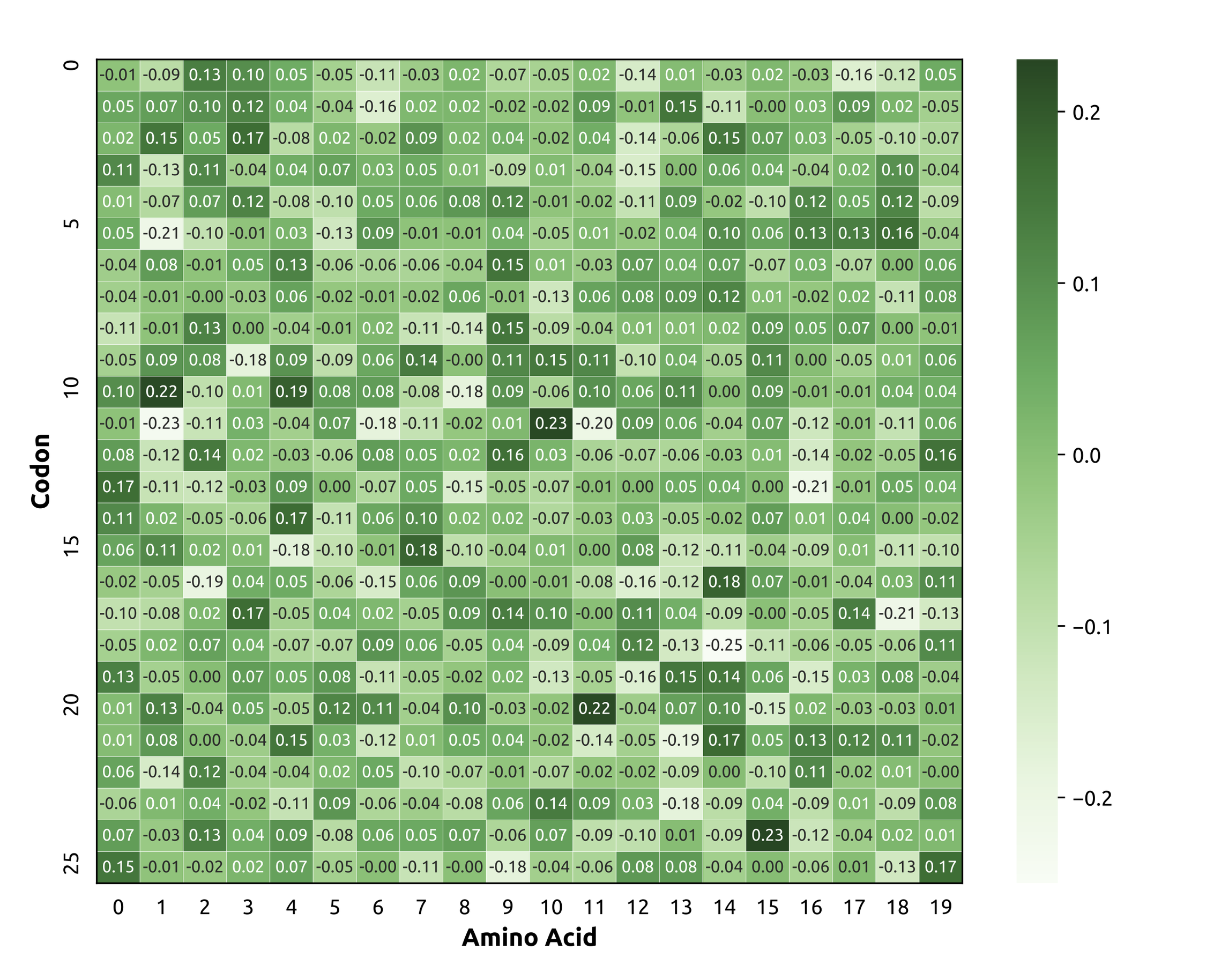

Supplement: S2 Fig — Only the first 26 codon positions (vertical axis) are shown for illustrative purposes. Each cell value indicates the potential contribution from each amino acid if present (horizontal axis) at the corresponding codon position (vertical axis). The cell values are sampled from a normal distribution with a mean of 0 (i.e., most random mutations are neutral). Cell colour indicates the magnitude of fitness, with darker colour representing fitness increase and lighter colour representing fitness decrease. (TIF) [file pcbi.1013295.s006.tif]

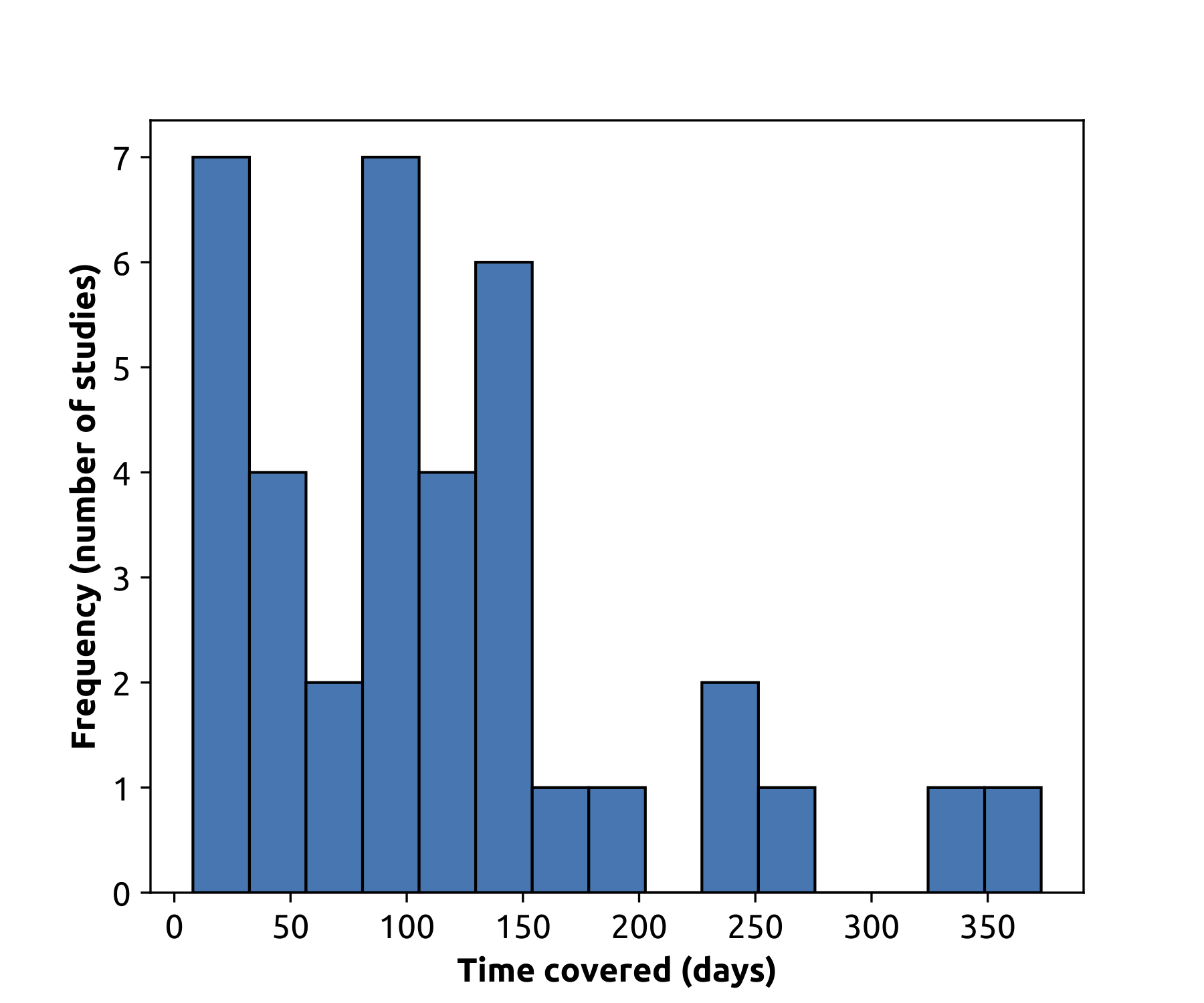

Supplement: S3 Fig — Datasets were obtained from [31,49], updated as of December 25, 2022. (TIF) [file pcbi.1013295.s007.tif]

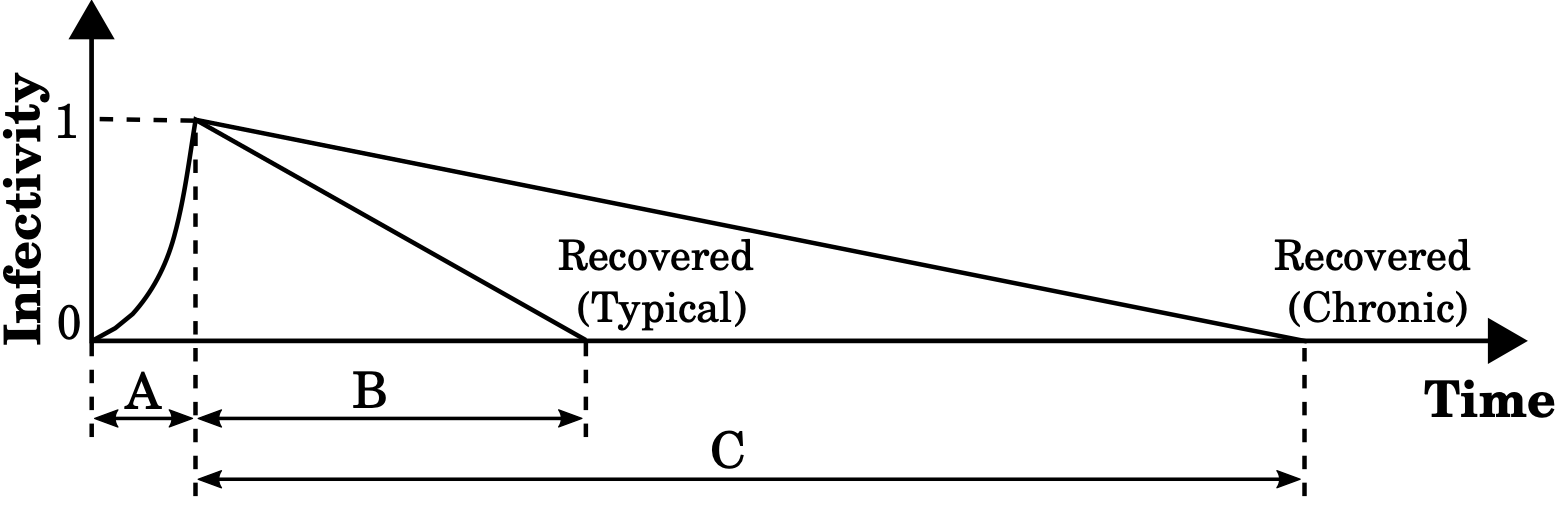

Supplement: S4 Fig — The infectivity initially increases exponentially from the onset of infection, reaching the peak, and subsequently declining linearly to zero until recovery. For each infected host, the duration from the infection onset to the infectivity peak is sampled from a lognormal distribution (A) with parameters μ=1.013 and σ=0.413. The recovery period is sampled from a uniform distribution, ranging from 7 to 11 days (B) for typical infections, or from 60 to 370 days (C) for chronic infections. (TIF) [file pcbi.1013295.s008.tif]

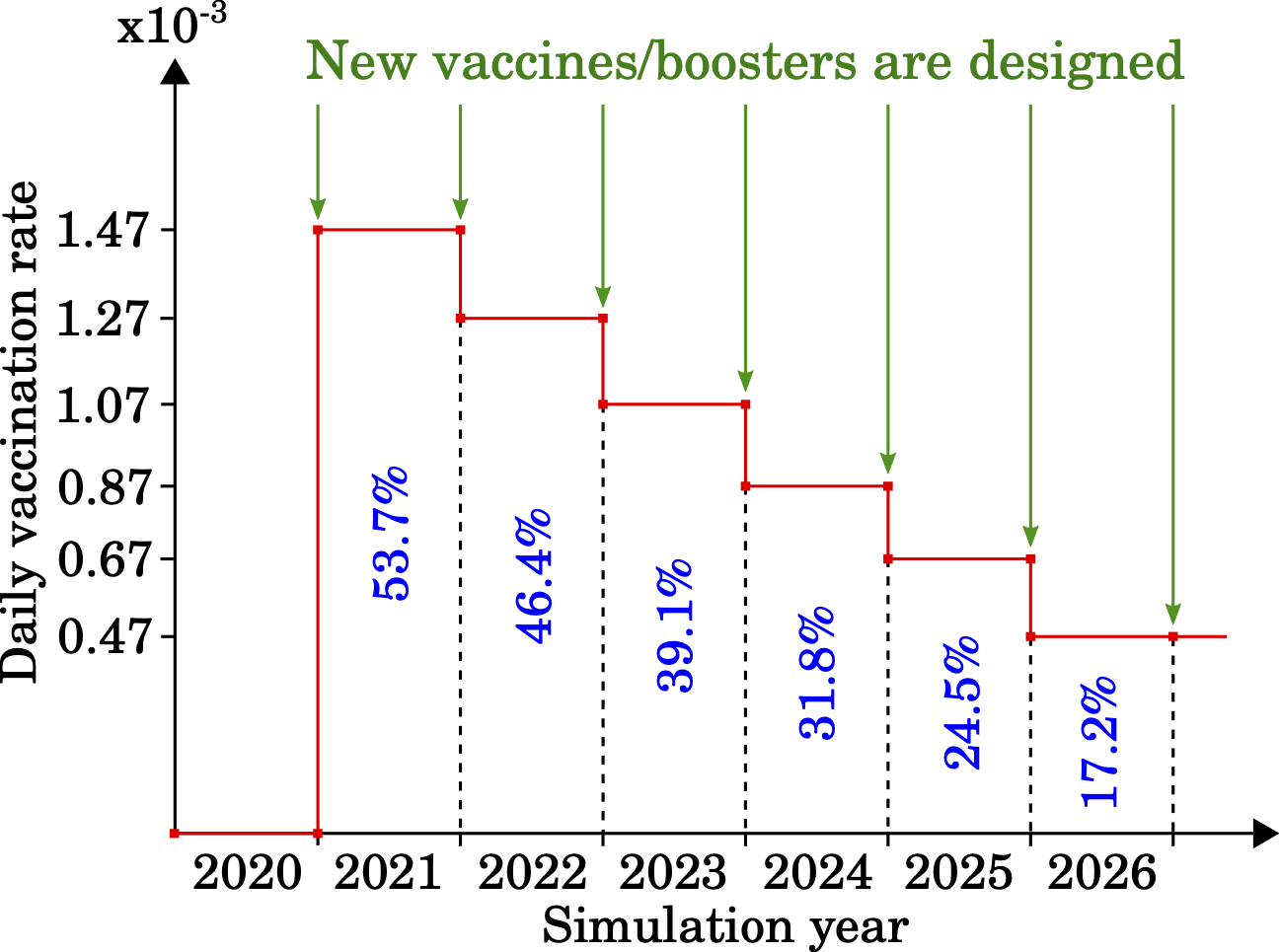

Supplement: S5 Fig — The percentage (in blue) within each bar represents the approximate percentage of the vaccinated population at the end of the year. (TIF) [file pcbi.1013295.s009.tif]

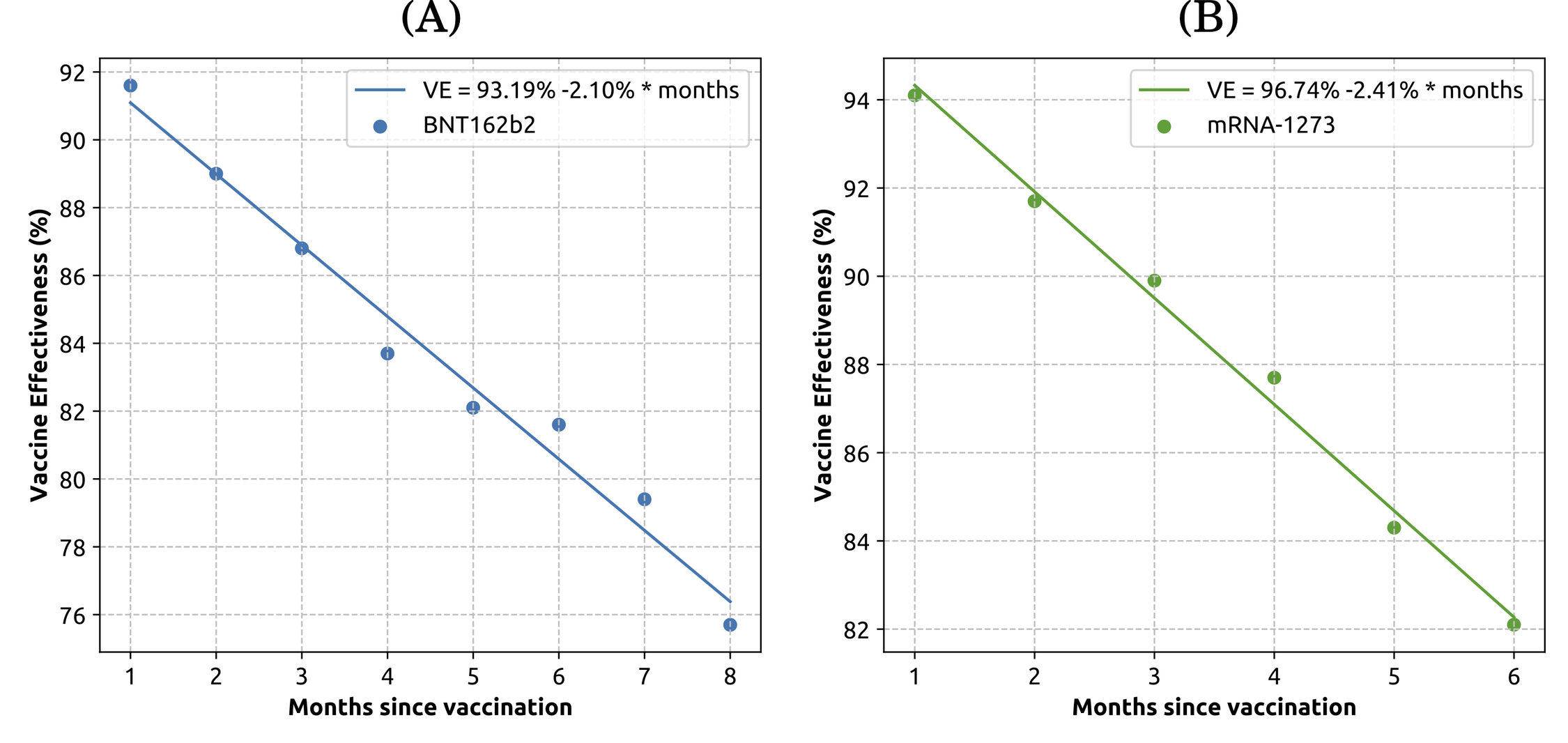

Supplement: S6 Fig — Two widely used mRNA vaccines are studied: (A) Pfizer (BNT162b2), and (B) Moderna (mRNA-1273), plotted and fitted using data from [50]. The equation shown in each plot represents the fitted linear regression for each vaccine, estimating a linear reduction in effectiveness from 2.1% to 2.4% per month. (TIF) [file pcbi.1013295.s010.tif]

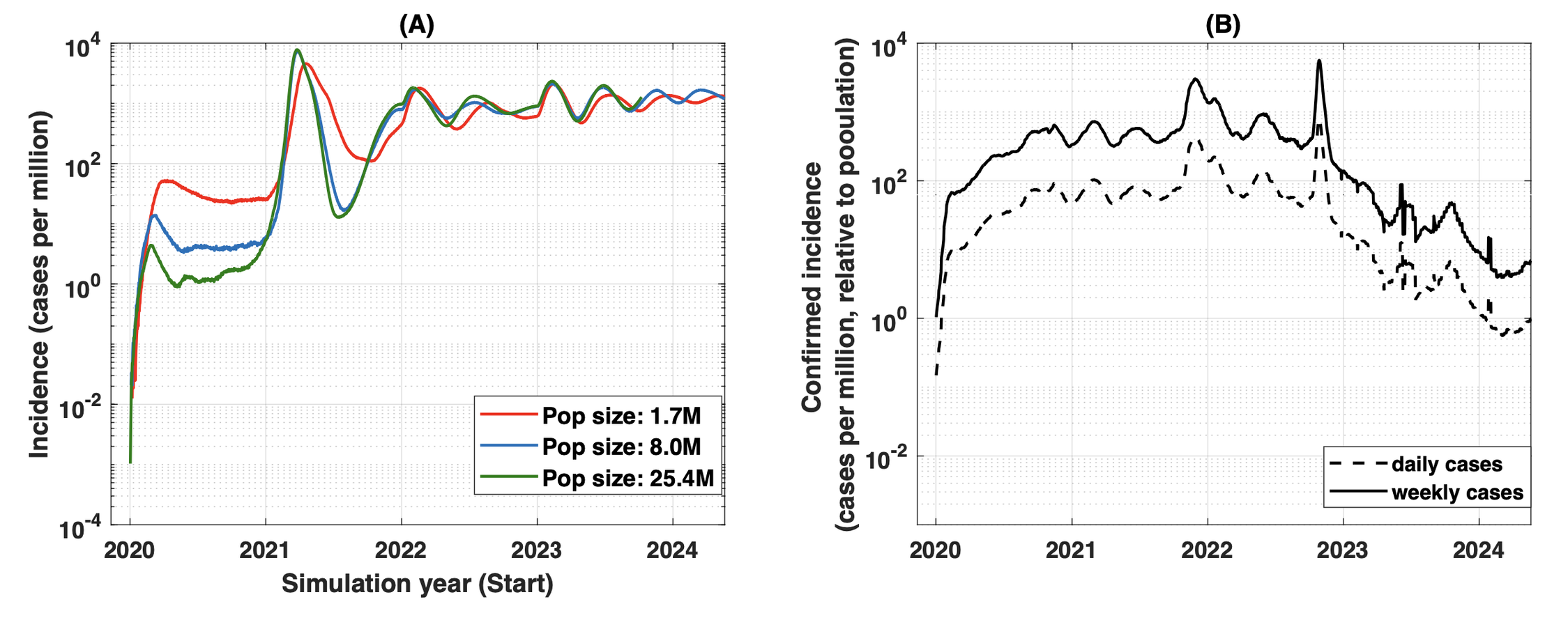

Supplement: S7 Fig — (A) Simulated incidence on a log scale in population sets of 1.7 million (red), 8 million (blue), and 25.4 million (green). This figure corresponds to Fig 6A on a linear scale. (B) Worldwide detected incidence [14], measured as new weekly cases per million (solid black line) and new daily cases per million (dashed black line). (TIF) [file pcbi.1013295.s011.tif]

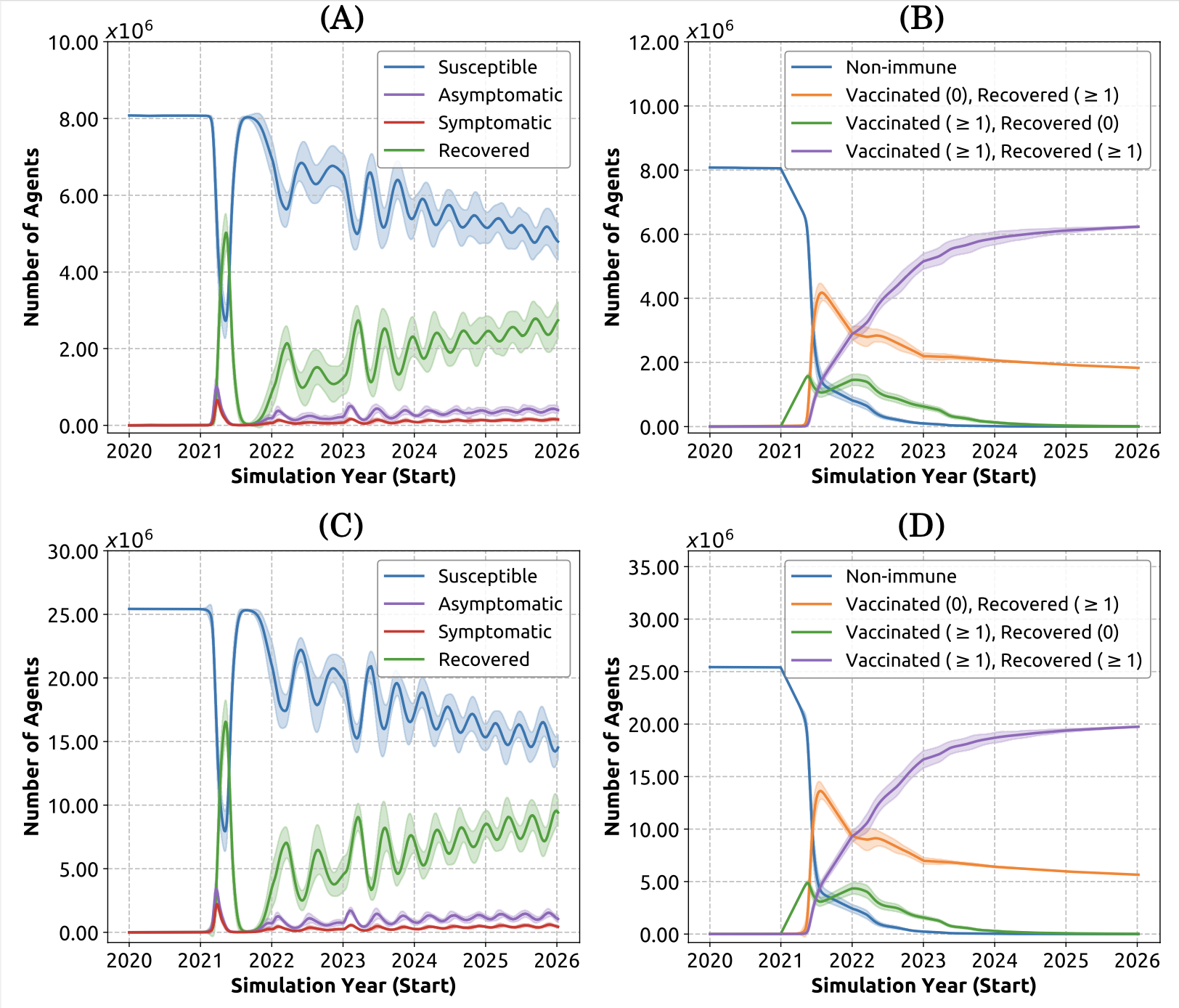

Supplement: S8 Fig — (A) and (C) Population across different health states, including susceptible (blue), asymptomatically infectious (purple), symptomatically infectious (red), and recovered (green) for the populations of 8 million and 25.4 million, respectively. (B) and (D) Population with different immunisation and infection history for the populations of 8 million and 25.4 million, respectively. Numbers in brackets denote the number of immunological (vaccination or infection) records. Individuals with multiple vaccinations or infections (more than 2) are grouped together for simplicity. The mean and average were obtained from approximately 30 realisations. (TIF) [file pcbi.1013295.s012.tif]

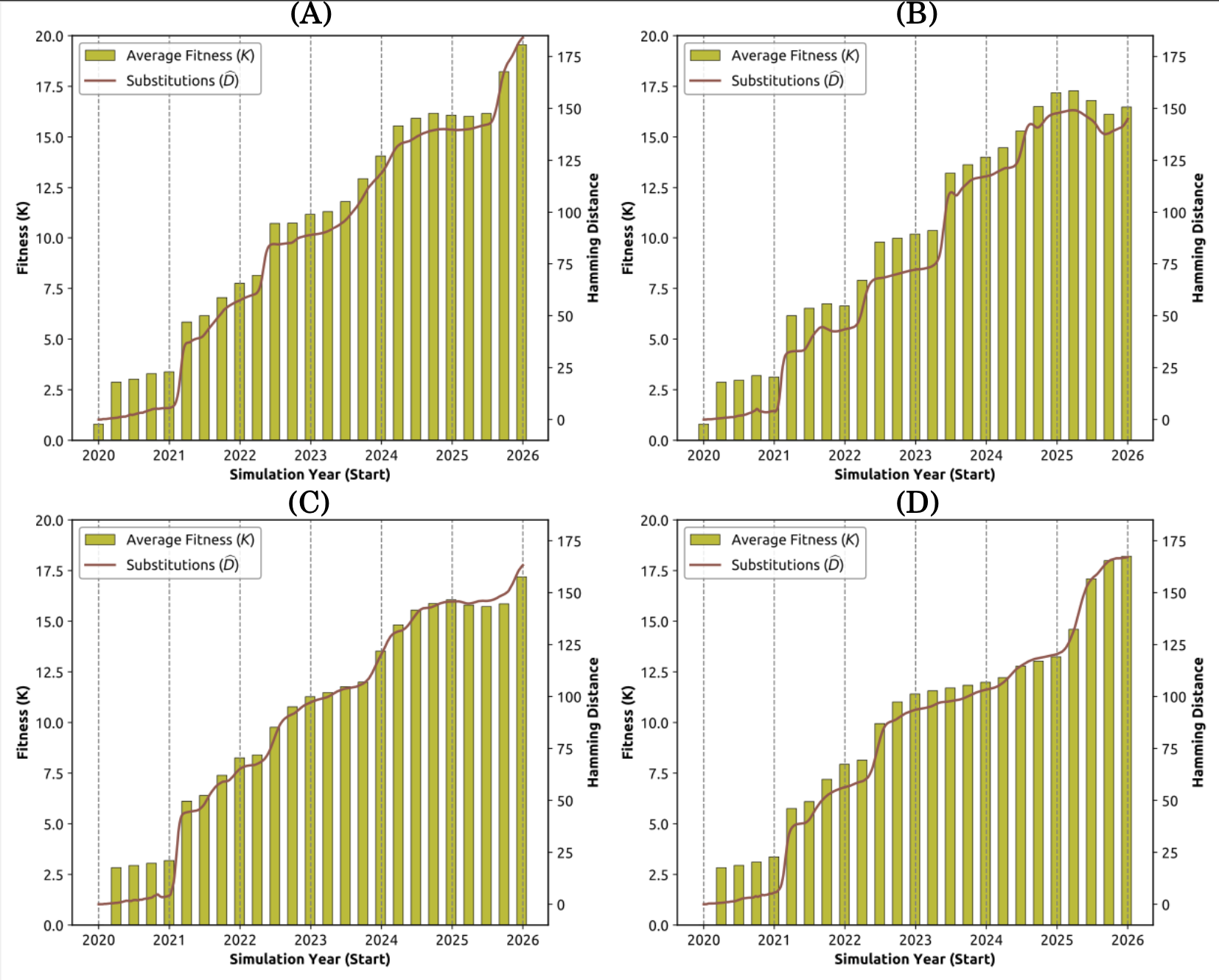

Supplement: S9 Fig — Fitness (K, olive bars, y-axis on the left), and the accumulated mutations (D^, solid brown line, y-axis on the right) are plotted from 2020 to 2026. Panels (A)–(D) are profiles plotted using four different realisations. (TIF) [file pcbi.1013295.s013.tif]

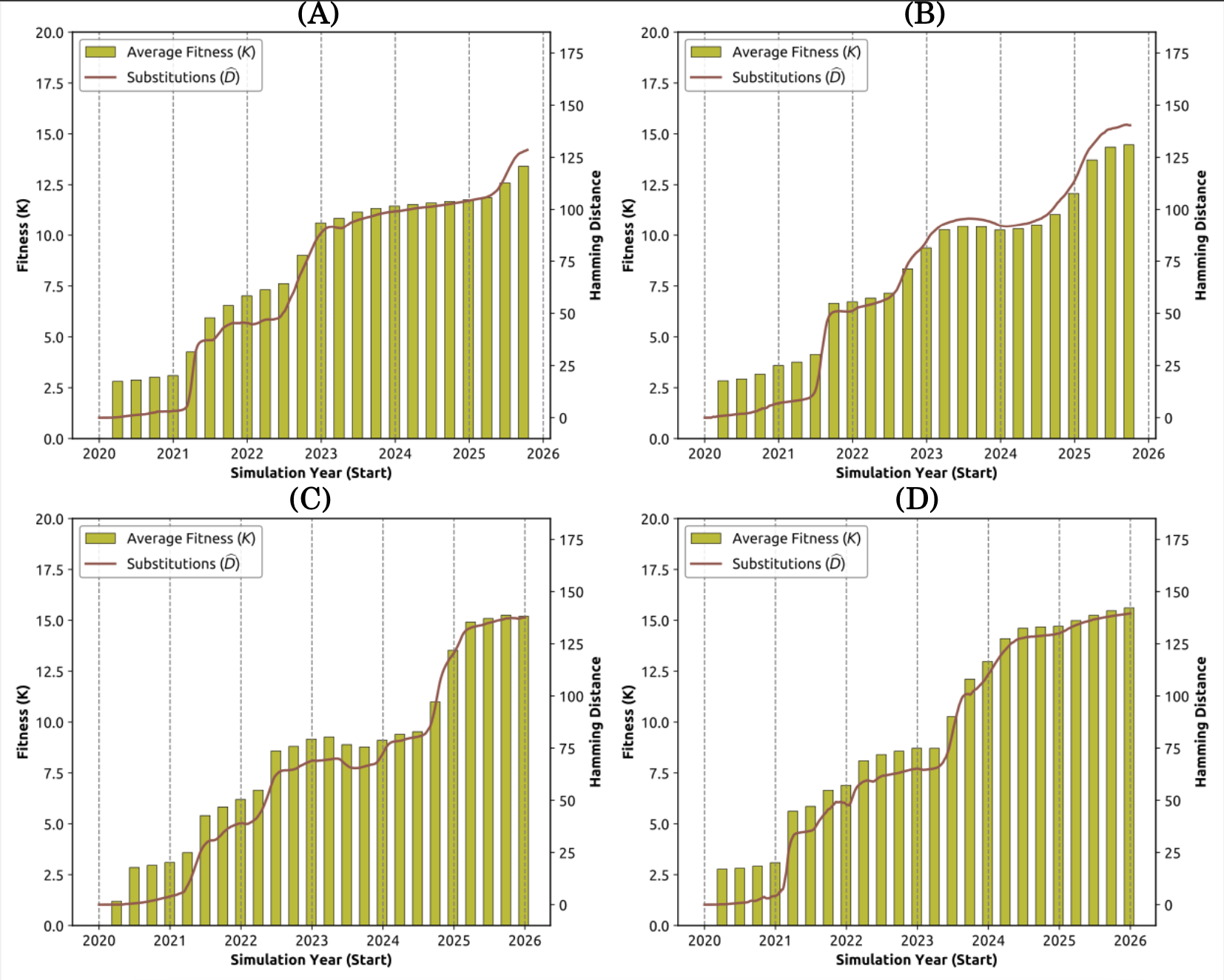

Supplement: S10 Fig — Fitness (K, olive bars, y-axis on the left), and the accumulated mutations (D^, solid brown line, y-axis on the right) are plotted from 2020 to 2026. Panels (A)–(D) are profiles plotted using four different realisations. (TIF) [file pcbi.1013295.s014.tif]

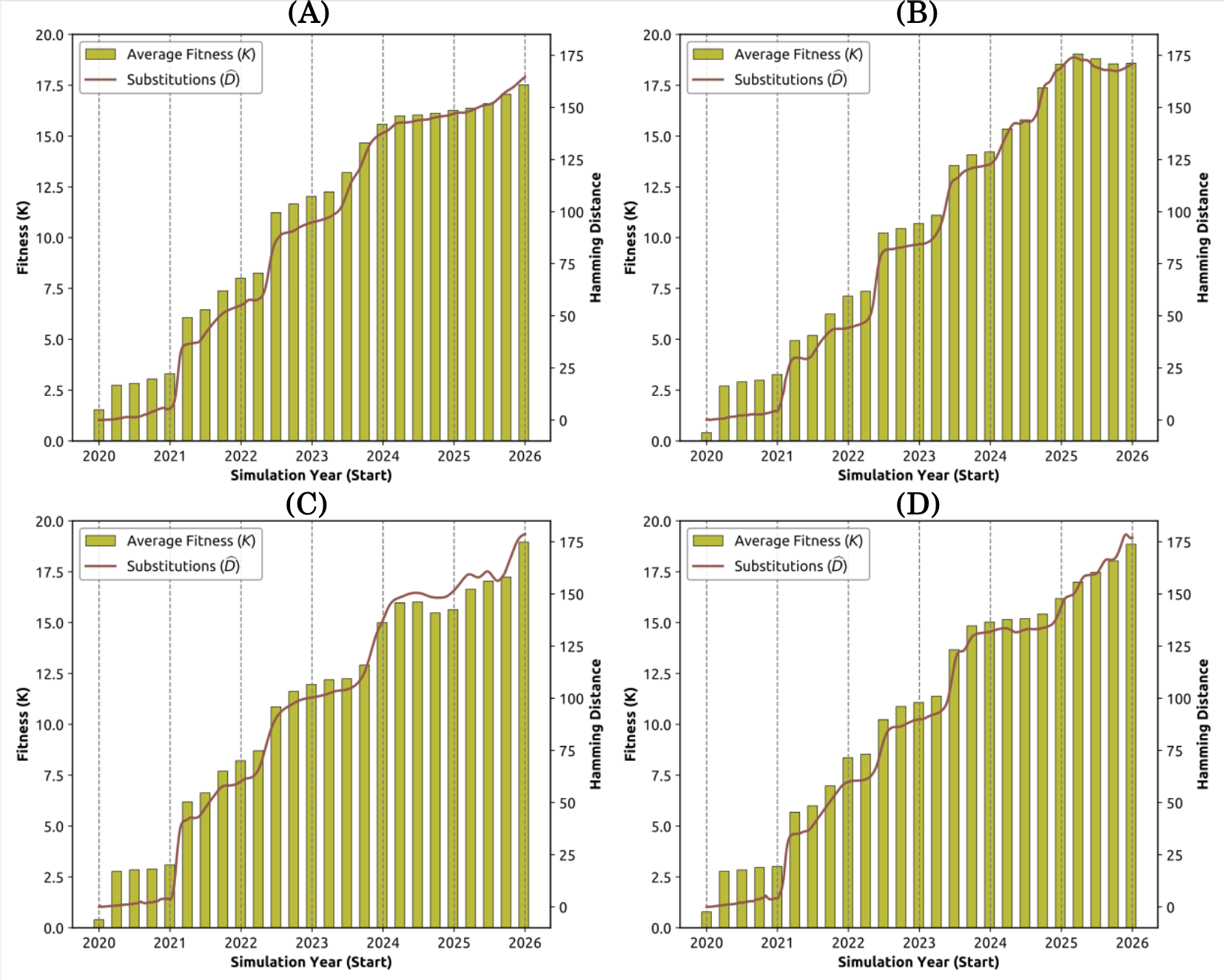

Supplement: S11 Fig — Fitness (K, olive bars, y-axis on the left), and the accumulated mutations (D^, solid brown line, y-axis on the right) are plotted from 2020 to 2026. Panels (A)–(D) are profiles plotted using four different realisations. (TIF) [file pcbi.1013295.s015.tif]

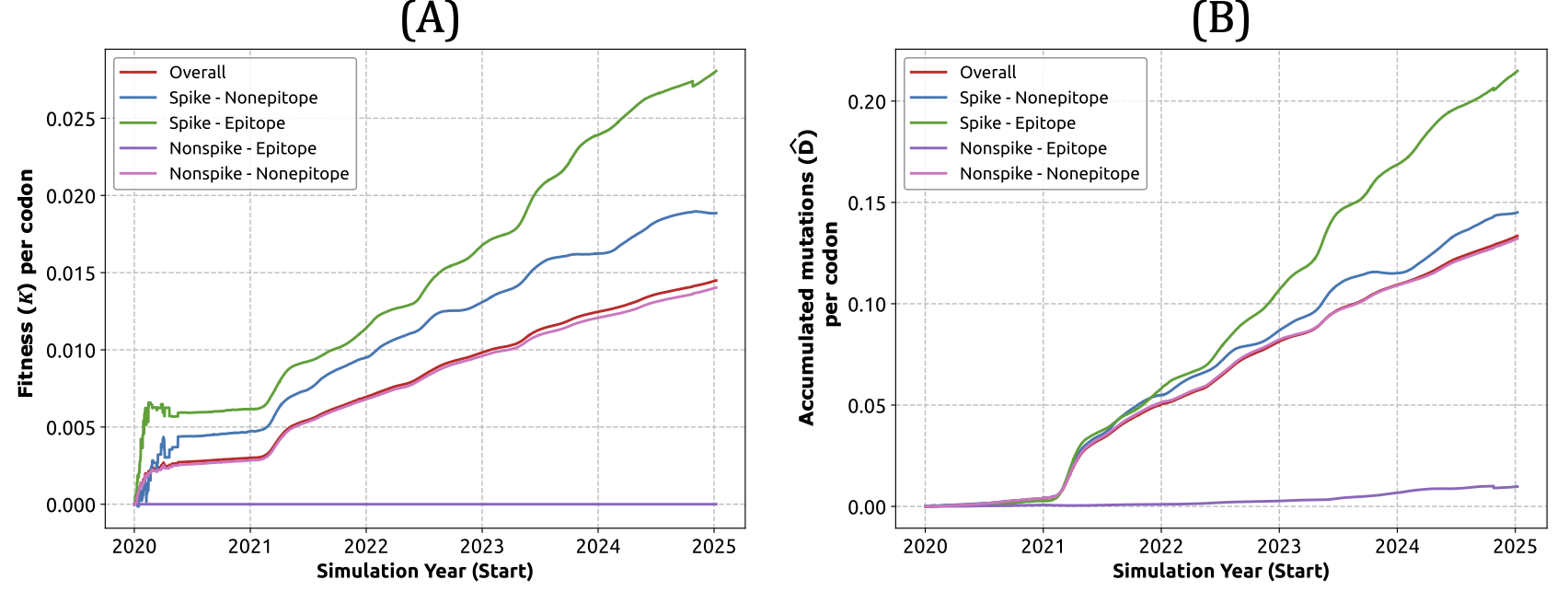

Supplement: S12 Fig — (A) Fitness K per codon, and (B) accumulated mutations D^ per codon. Five genome regions are plotted: overall genome (red), spike and non-epitope region (blue), spike and epitope (green), non-spike and epitope (purple), and non-spike and non-epitope (pink). (TIF) [file pcbi.1013295.s016.tif]

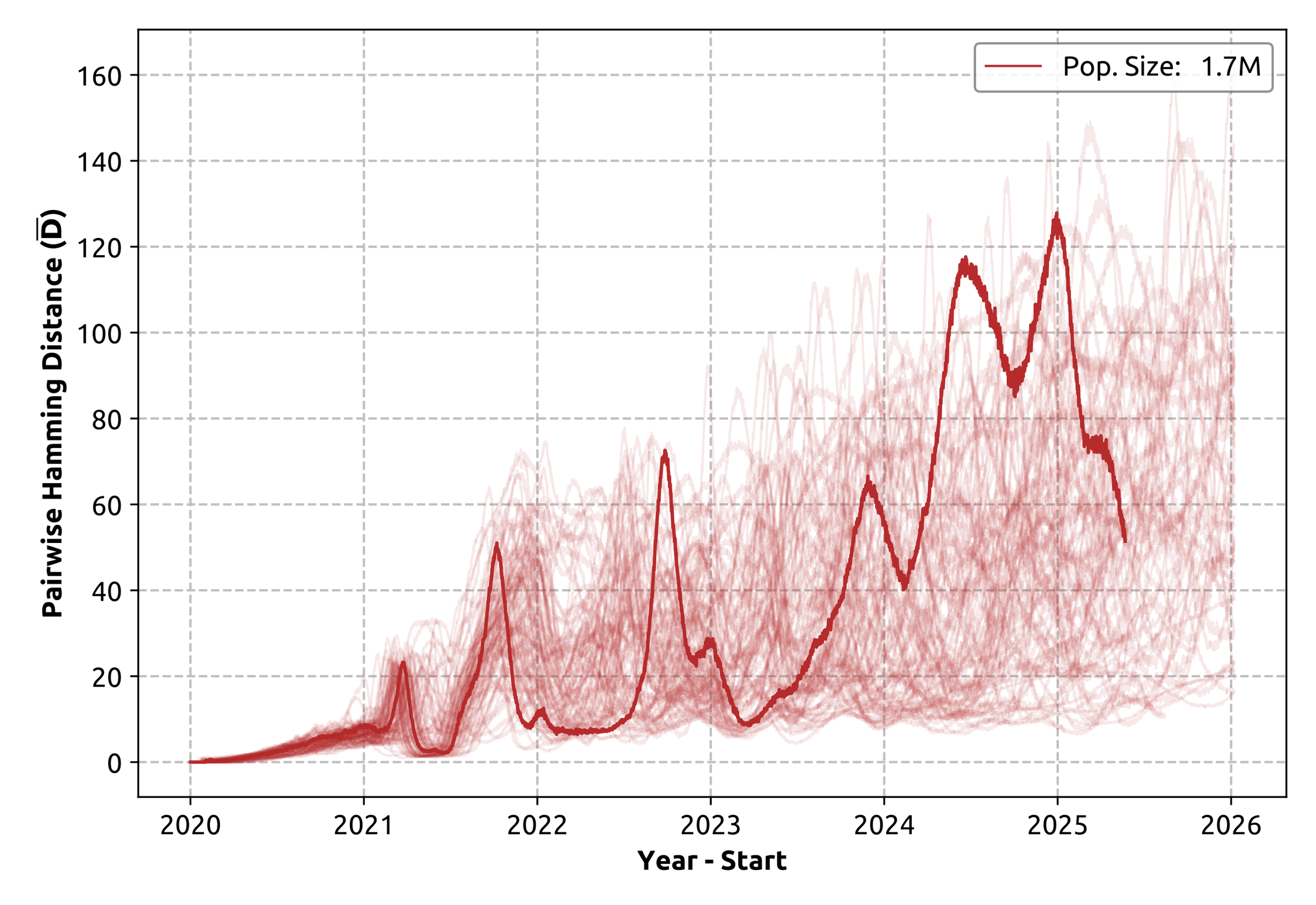

Supplement: S13 Fig — Approximately 10,000 pairs of genomes are randomly sampled at each simulation time point from 2020 to 2026 (Capability 2(i)). Opaque red lines represent the ensemble of all realisations and the solid red line shows the dynamics of one realisation only. (TIF) [file pcbi.1013295.s017.tif]

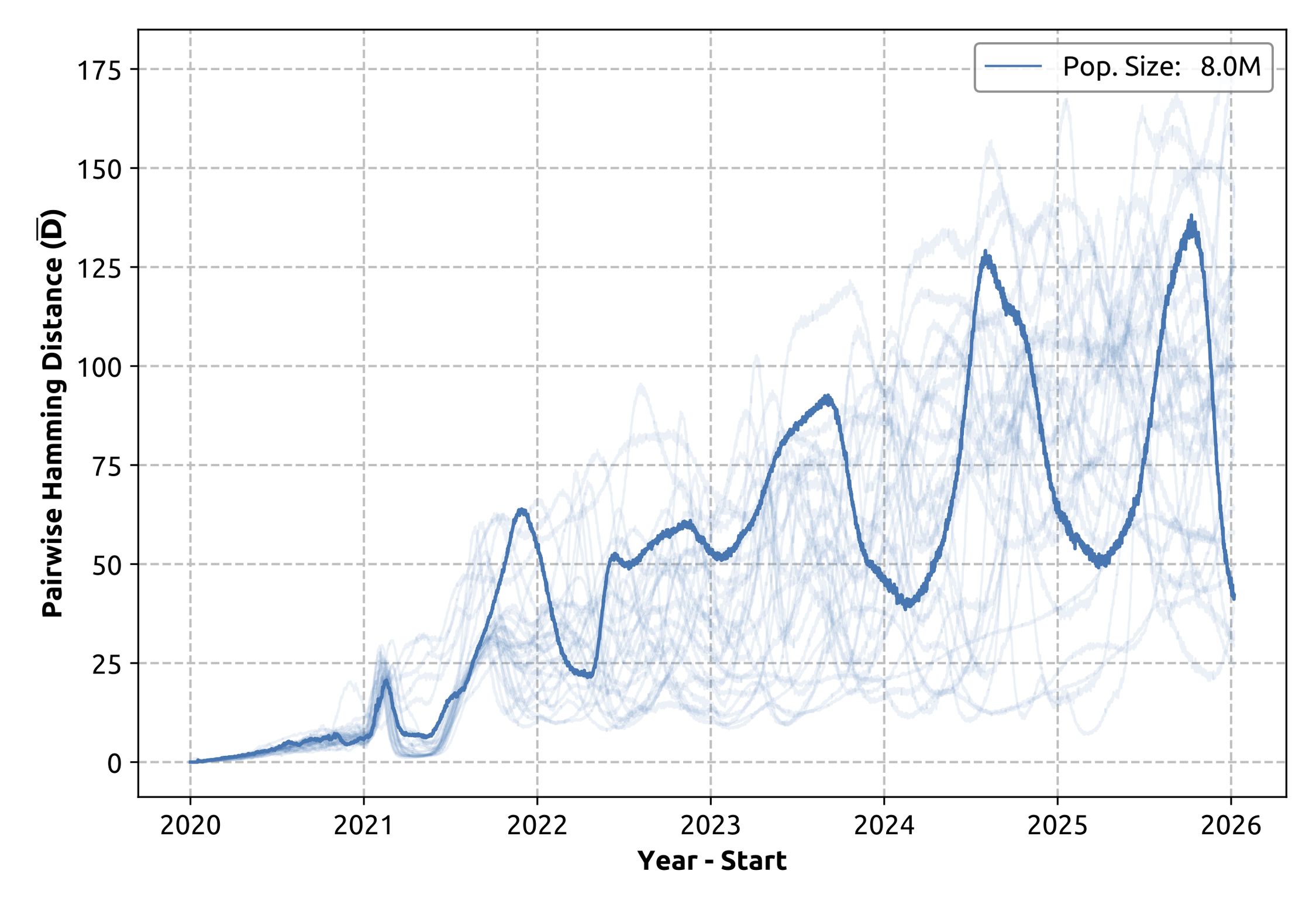

Supplement: S14 Fig — Approximately 10,000 pairs of genomes are randomly sampled at each simulation time point from 2020 to 2026 (Capability 2(i)). Opaque blue lines represent the ensemble of all realisations and the solid blue line shows the dynamics of one realisation only. (TIF) [file pcbi.1013295.s018.tif]

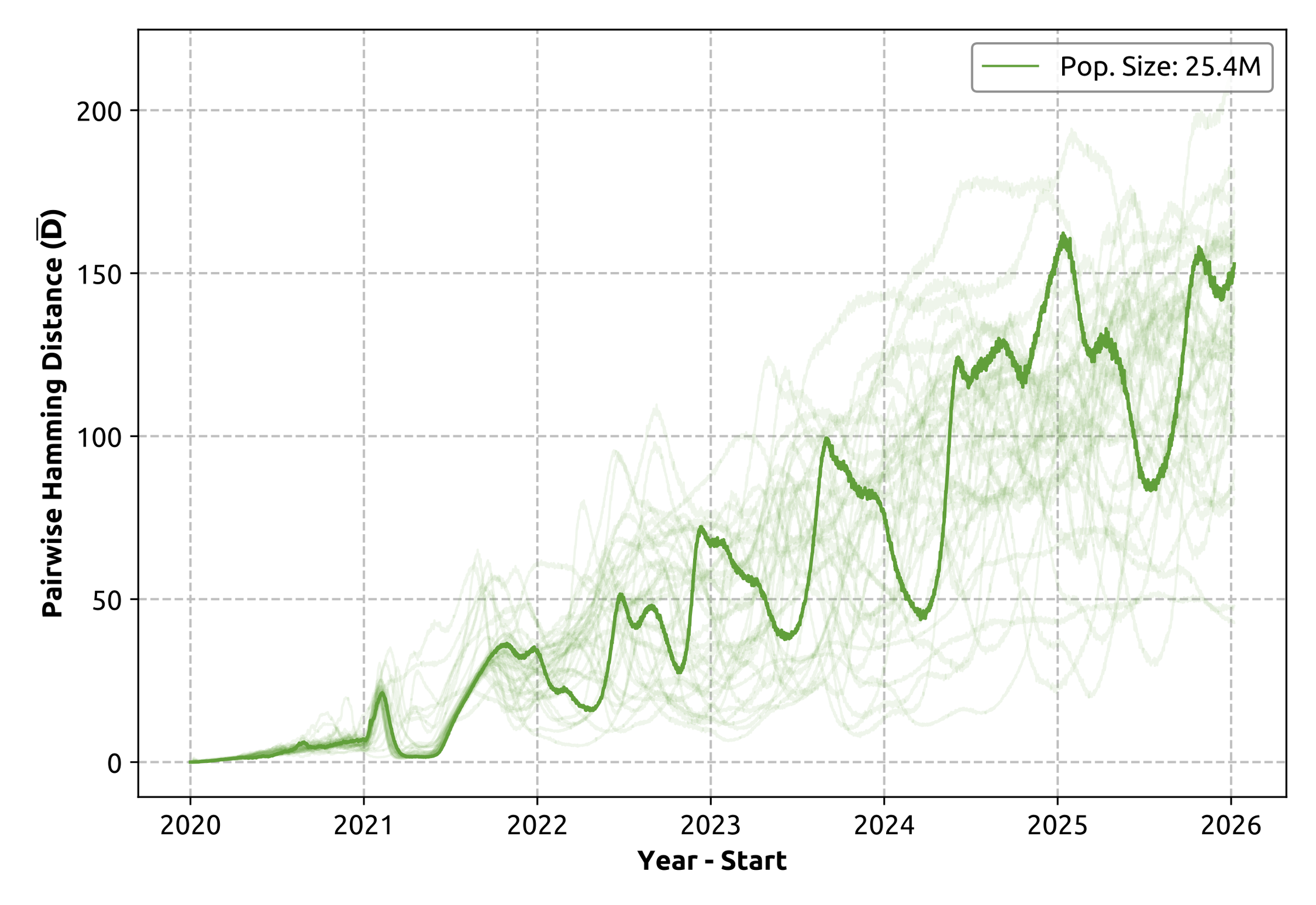

Supplement: S15 Fig — Approximately 10,000 pairs of genomes are randomly sampled at each simulation time point from 2020 to 2026 (Capability 2(i)). Opaque blue lines represent the ensemble of all realisations and the solid blue line shows the dynamics of one realisation only. (TIF) [file pcbi.1013295.s019.tif]

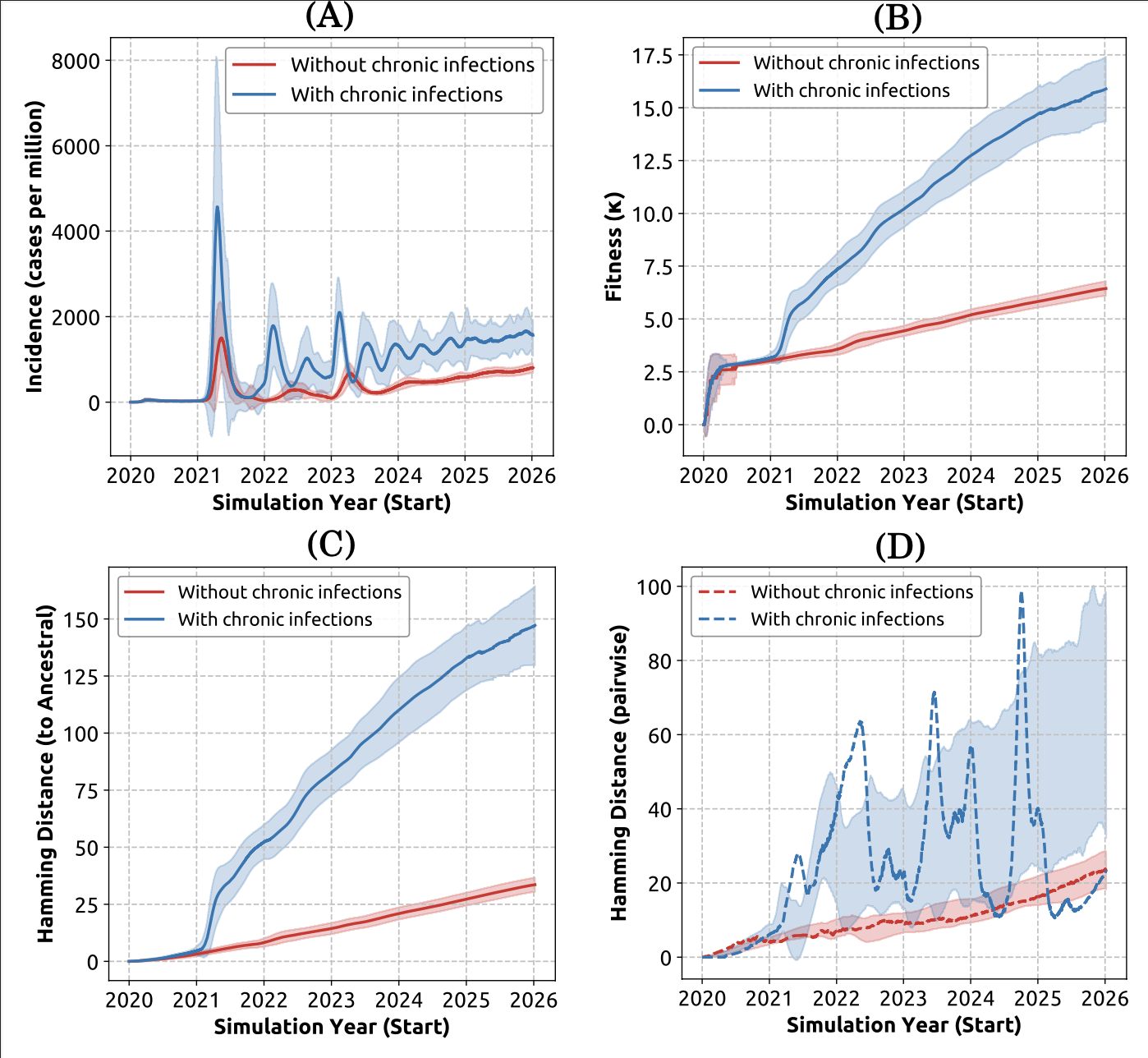

Supplement: S16 Fig — Simulated dynamics in scenarios with the fraction of agents susceptible to chronic infections being 0.1% of the entire population (blue curves), and without chronic infections (red curves). The profiles trace detected (A) incidence, (B) average pathogen transmissibility, interpreted as fitness K, (C) accumulated mutations (D^), and (D) genomic diversity (D―) in the population of 1.7 million, over six simulation years. The shaded area represents the range of one standard deviation from the mean values. In (A) to (C), the solid lines denote the mean value across 30–50 realisations. In (D), the dashed line denotes the pairwise Hamming distance from one realisation only, given the high variability between realisations. (TIF) [file pcbi.1013295.s020.tif]

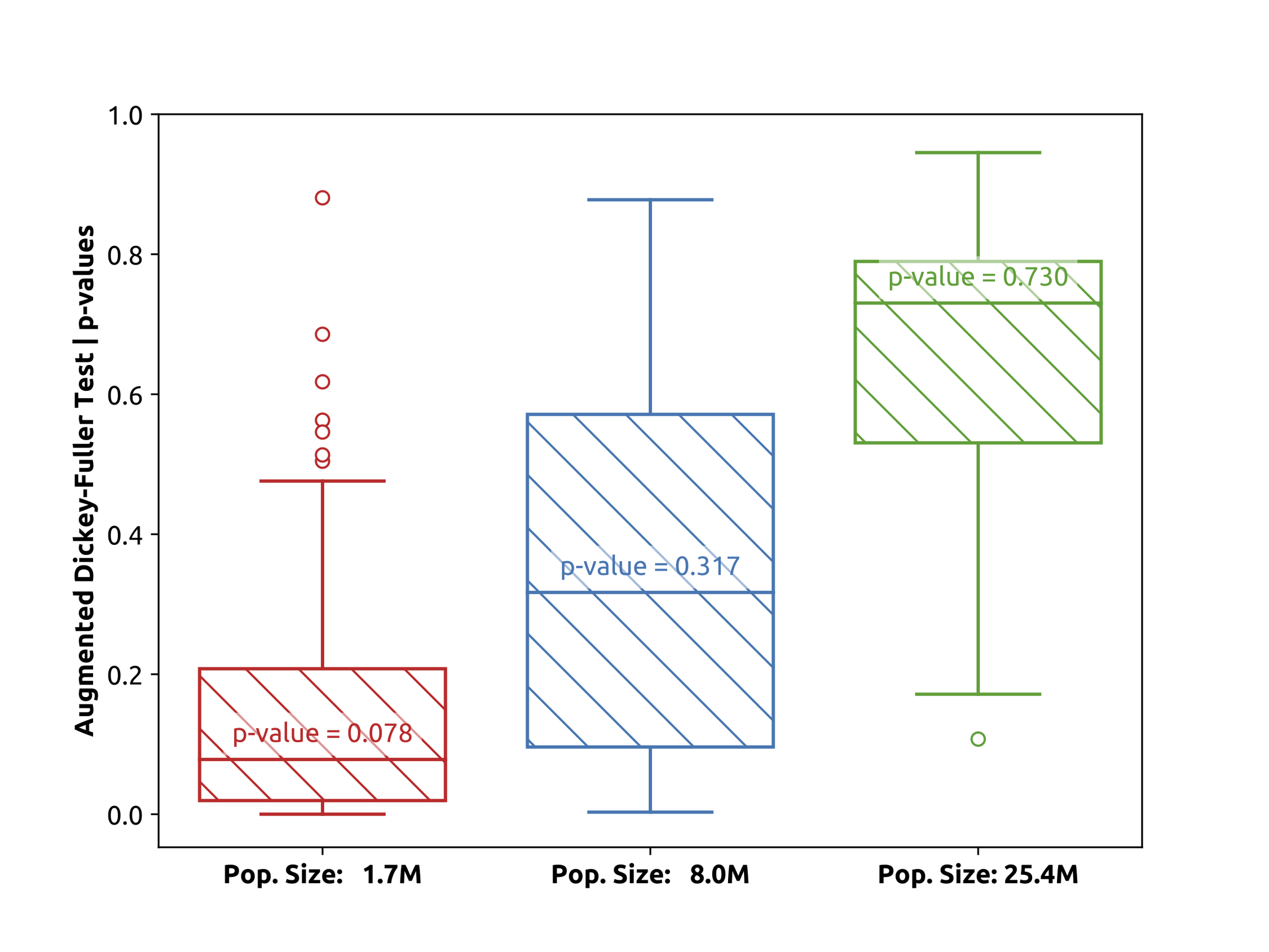

Supplement: S17 Fig — The stationarity of the time evolution of the average pairwise Hamming distance is computed between two randomly selected genomes. Approximately 10,000 pairs of genome sequences are randomly sampled at each simulation time point from 2020 to 2026 for three simulation scenarios with different population sizes of 1.7 million (A), 8 million (B), and 25.4 million (C) (Capability 3(ii)). (TIF) [file pcbi.1013295.s021.tif]

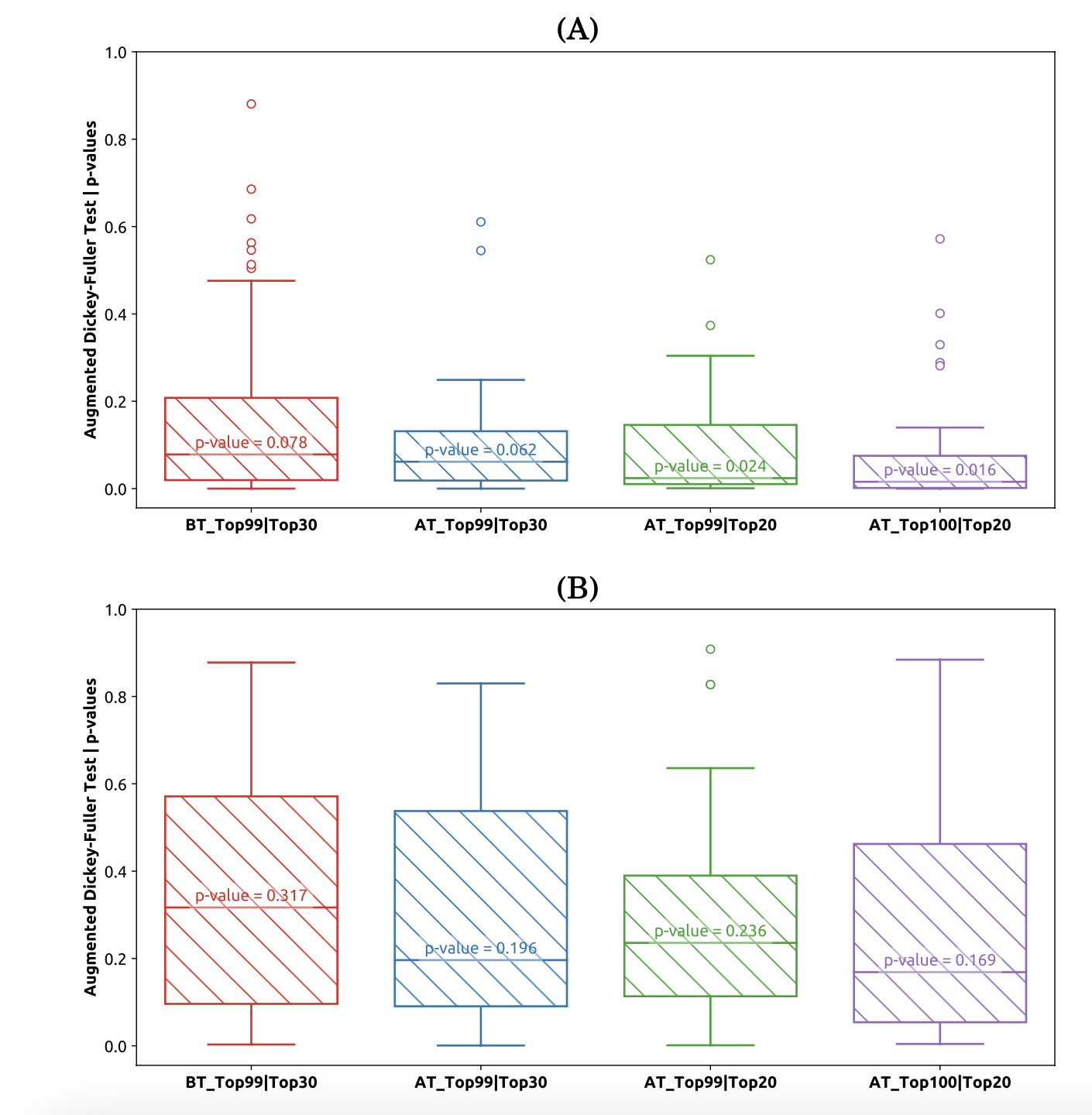

Supplement: S18 Fig — (A) populations of 1.7 million and (B) 8.0 million. Approximately 10,000 pairs of genome sequences are randomly sampled at each simulation time point from 2020 to 2026 for four simulation scenarios by varying amino acid weight table and/or within-host selective pressure (see bar legends). We tested two different fitness weight tables following different normal distributions: (i) BT: the baseline setting, where each amino acid contribution in spike and non-spike codon positions is sampled from the normal distributions N(0, 0.085) and N(0, 0.07), respectively, and (ii) AT: the alternative setting, where each amino acid contribution in spike and non-spike codon positions is sampled from the normal distributions N(0, 0.0489) and N(0, 0.0454), respectively. Within-host selective pressure is varied for both typical infected hosts and chronically infected hosts, using notation separated by a vertical bar (e.g., Top99|Top30 represents a setting where selective pressure at top 99 for typically infected hosts (X=99, M = 100) and at top 30 for chronically infected hosts (X=30, M = 100). (TIF) [file pcbi.1013295.s022.tif]

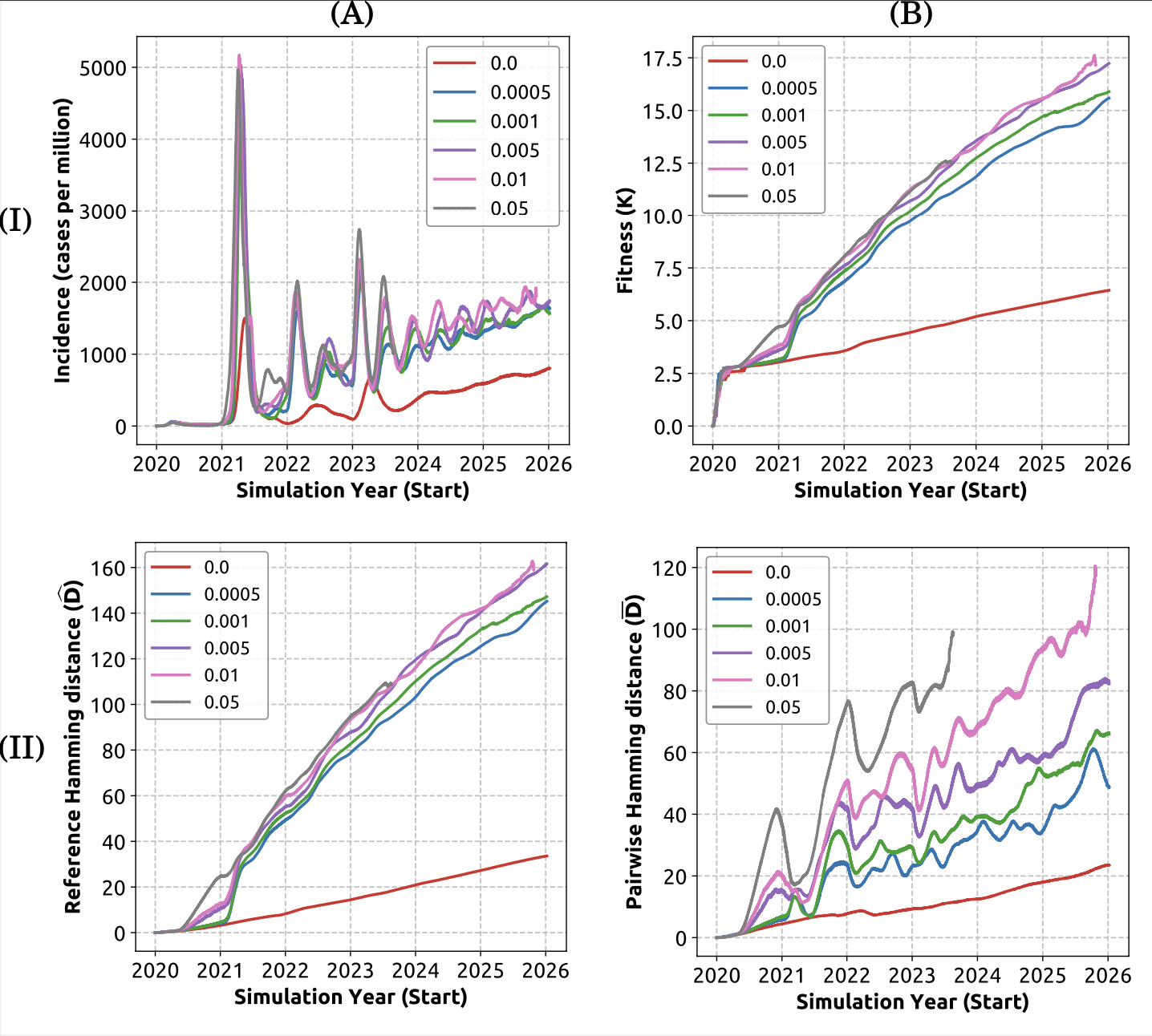

Supplement: S19 Fig — Simulated dynamics of the detected incidence (I-A), average pathogen fitness (I-B), average accumulated mutations D^ (II-A), and average genomic diversity D― (II-B) (one realisation only), in a population of 1.7 million. These measures are traced while the fraction of individuals susceptible to chronic infection, with strong positive within-host selective pressure, is varied from 0.0 to 0.05 of the total population. (TIF) [file pcbi.1013295.s023.tif]

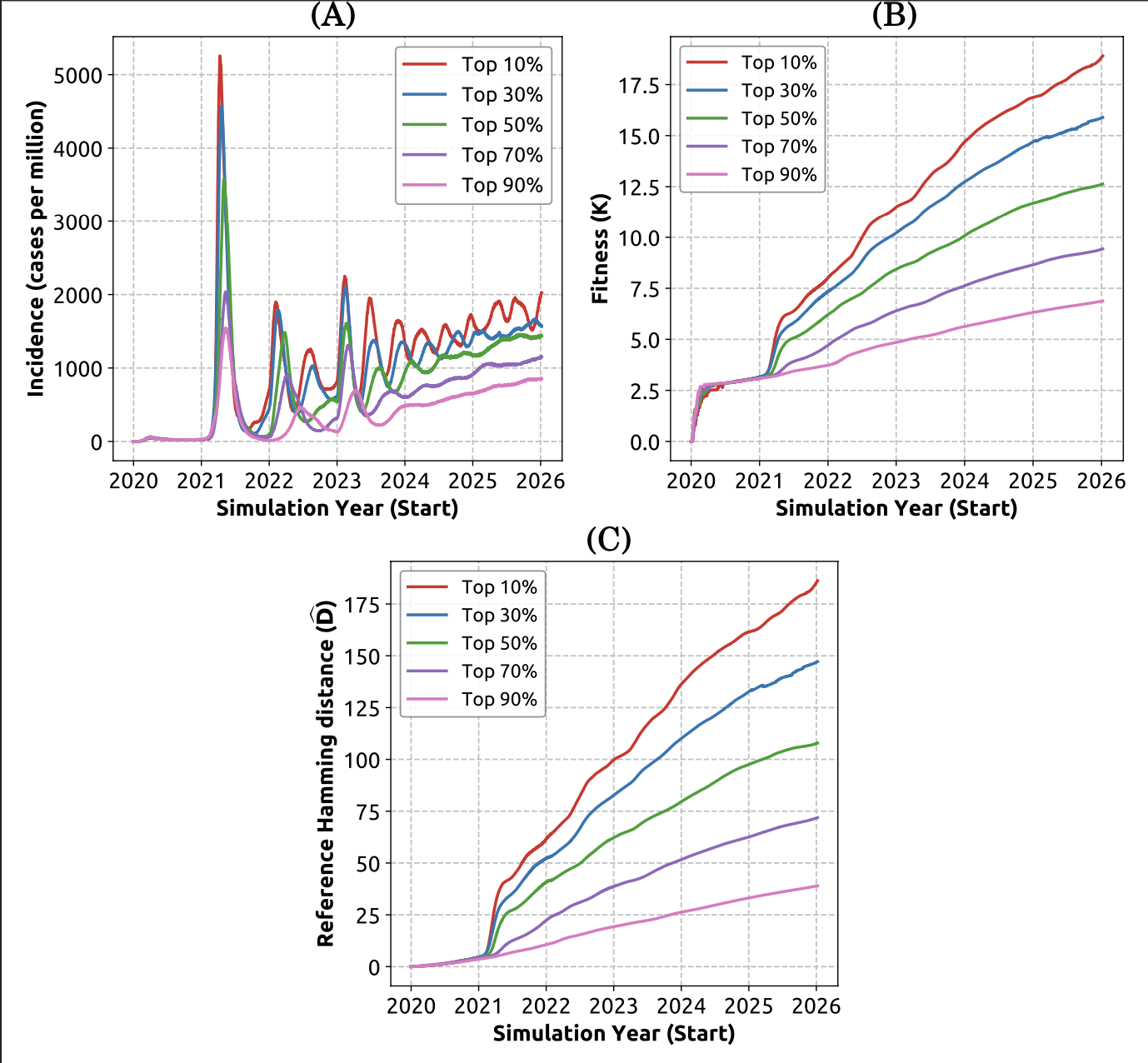

Supplement: S20 Fig — Simulated dynamics of (A) detected incidence, (B) average pathogen fitness, and (C) average accumulated mutations D^ in a population of 1.7 million. The within-host selective pressure for chronically infected hosts, starting from day 60 of infection, is varied by selecting, in each simulation cycle, the mutated genomes from the top 10% (high selectivity, X = 10, M = 100) to the top 90% (low selectivity, X = 90, M = 100) of the ranked list of mutated genome candidates. (TIF) [file pcbi.1013295.s024.tif]

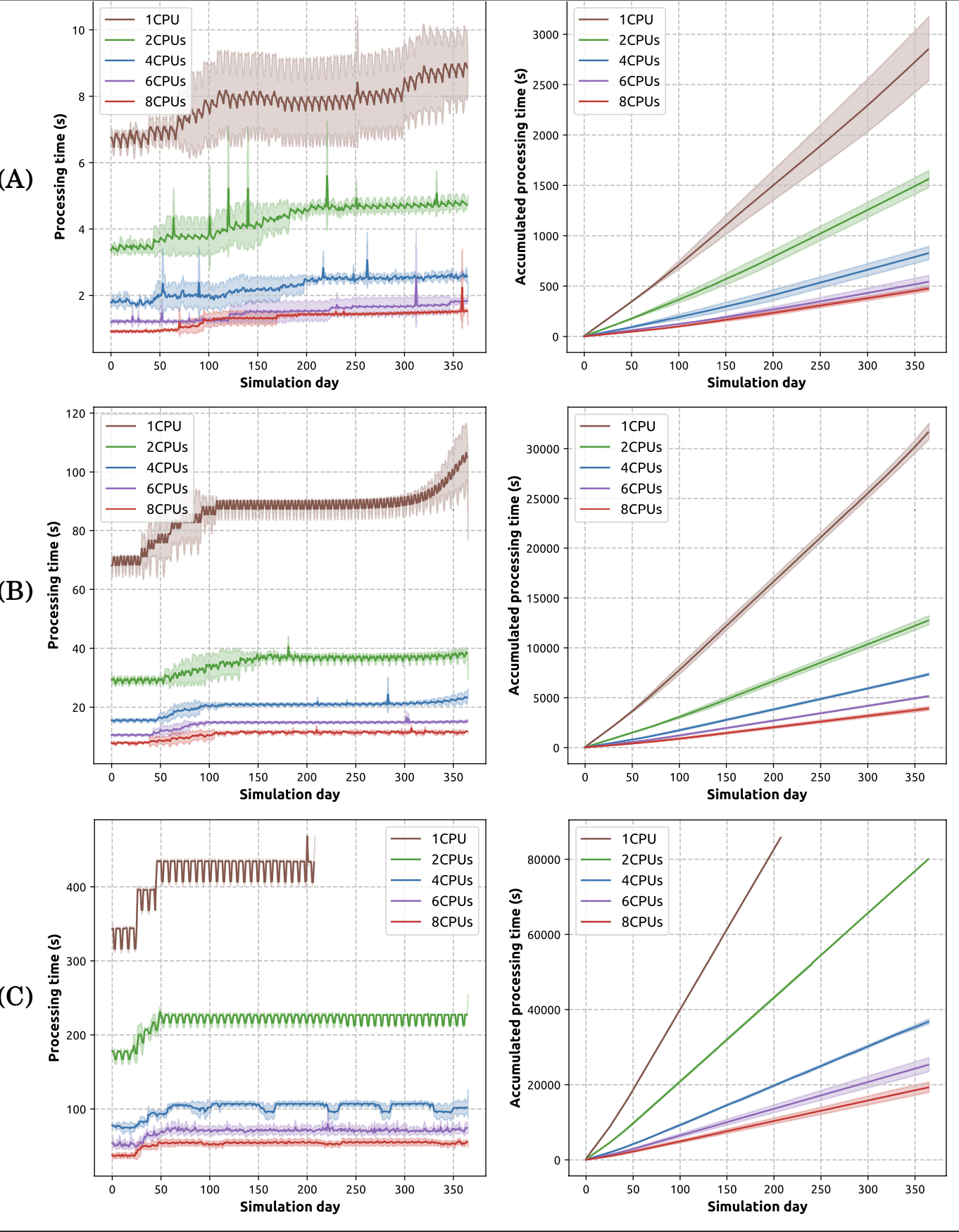

Supplement: S21 Fig — The simulation covers 365 simulation days for three different population sizes: (A) 230K agents, (B) 1.7M agents, and (C) 8M agents. Left: the processing time required to simulate a single simulation day. Right: the cumulative processing time required to simulate 365 days. Confidence intervals are shown as shaded areas. (TIF) [file pcbi.1013295.s025.tif]

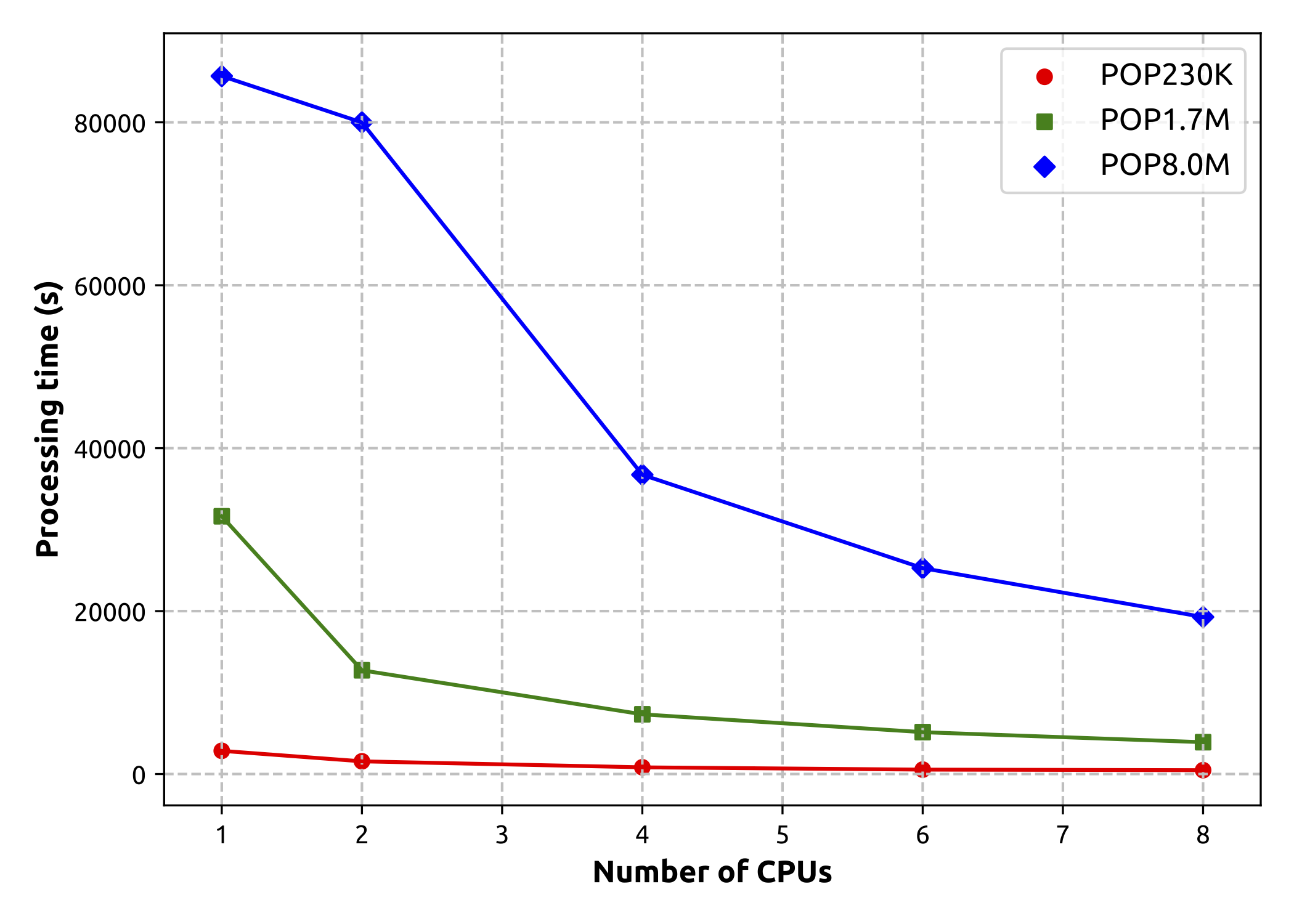

Supplement: S22 Fig — Population sizes: 230K agents (red), 1.7M agents (green), and 8M agents (blue). (TIF) [file pcbi.1013295.s026.tif]

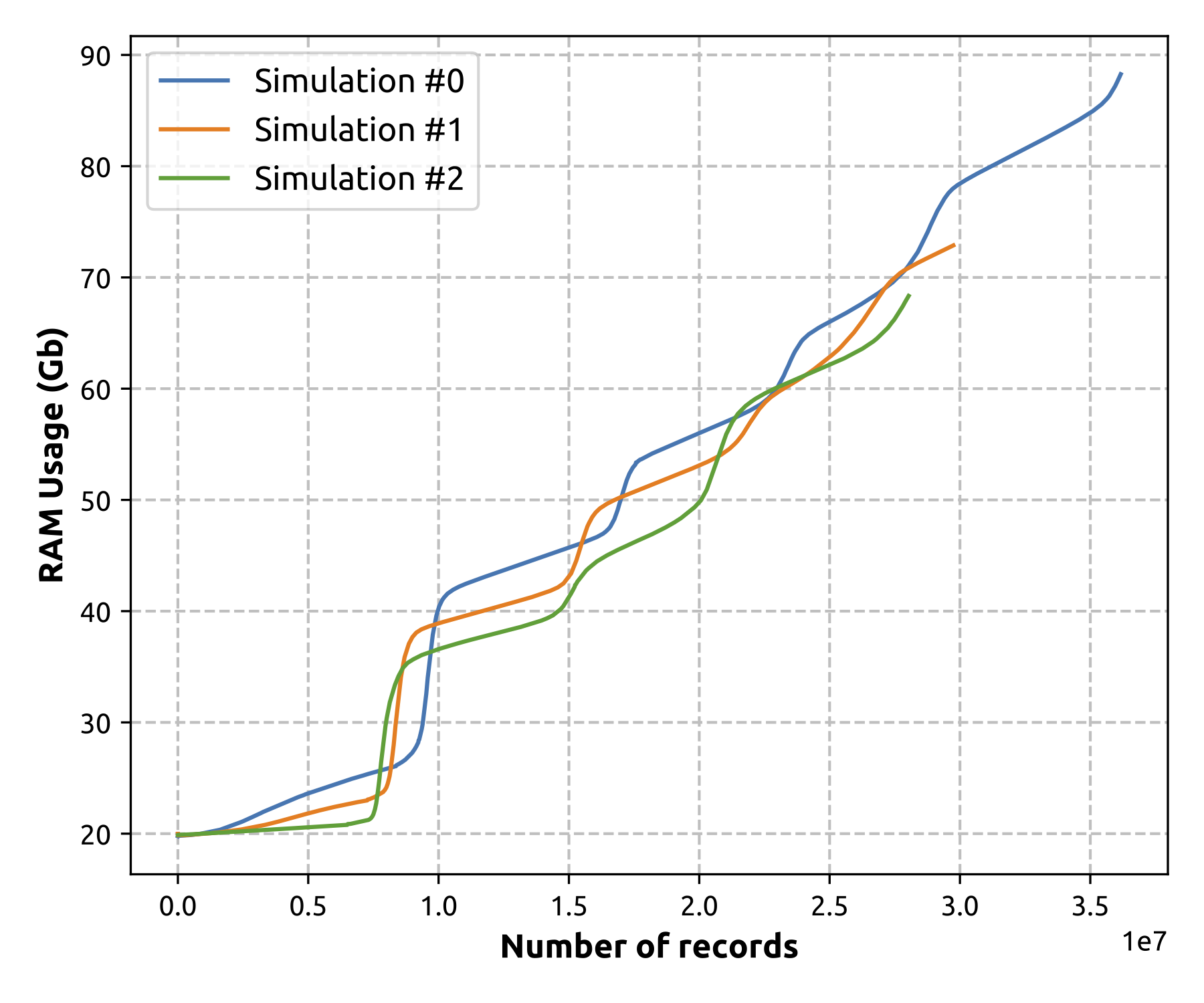

Supplement: S23 Fig — The immunological records contain vaccination and infection histories of 8 million agents. Each profile represents an individual realisation. All realisations are simulated under identical inputs and settings. (TIF) [file pcbi.1013295.s027.tif]
