# Supplementary material for: Multi-scale phylodynamic modelling of rapid punctuated pathogen evolution
Source: PLoS Comput Biol. 2025 Jul 14;21(7):e1013295. doi: 10.1371/journal.pcbi.1013295 (PMC12270310; doi:10.1371/journal.pcbi.1013295)
Supplement: S1 Text — (PDF) [file pcbi.1013295.s028.pdf]

# Multi-scale phylodynamic modelling of rapid punctuated pathogen evolution: Supplementary text

Quang Dang Nguyen [1] ¶, Sheryl L. Chang[1,2] ¶, Carl J. E. Suster [2,3], Rebecca J. Rockett[2,3], Vitali Sintchenko [2], Tania C. Sorrell [2], Mikhail Prokopenko[1,2] *

**1** Centre for Complex Systems, The University of Sydney, Sydney, Australia
**2** Sydney Infectious Diseases Institute, The University of Sydney, Sydney, Australia
**3** Centre for Infectious Diseases and Microbiology–Public Health, Westmead Hospital, Westmead, Australia

¶ These authors contributed equally to this work.
*mikhail.prokopenko@sydney.edu.au

**S1 Text. Overview of supplementary text.**
The phylodynamic agent-based model implemented in PhASE TraCE synthesises two parts: the within-host evolution using a phylogenetic model, and the inter-host transmission using a high-resolution agent-based model. This simulator comprises four processing layers: (A) Phylogenetic, (B) Demographic, (C) Epidemic, and (D) Immunological, each with various data flows updating core attributes of *Agent* objects. These agent attributes include: (i) health status, (ii) genome representation of the pathogen if infected, (iii) the social groups with which an agent may interact, and (iv) immunological history of past infections and vaccinations. The PhASE TraCE architecture is described in Methods (subsection Multi-layer architecture of PhASE TraCE).

The processing layers are detailed in supplementary text as follows:

- (A) Phylogenetic Layer: Section (A) Phylogenetic model describes the model of the within-host evolution.
- (B) Demographic Layer: Section (B) Artificial agent-based population details the generation and structure of the artificial heterogeneous population.
- (C) Epidemic Layer: Section (C) Multi-strain transmission and control describes the disease transmission model in terms of agent interactions, in presence of multiple co-circulating pathogens characterised by different genomes (subsection Multi-strain transmission model), as well as the control model of multiple non-pharmaceutical interventions and their impact on disease transmission (subsection Non-pharmaceutical interventions).
- (D) Immunological Layer: Section (D) Immuno-epidemiological model details modelling of vaccination roll-outs and vaccine-induced immunity (subsection Vaccination), followed by a description of how the immunity changes over time during pathogen transmission and evolution (subsection Compound immunity and waning effects). This section also describes how the within-host pathogen evolution affects inter-agent infection transmission, under the effect of compound and waning immunity generated by the immunological history.

Supplementary text also includes some additional results, sensitivity analysis and details on the computational complexity and implementation of PhASE TraCE.

# (A) Phylogenetic model

A key part of PhASE TraCE is a phylogenetic model developed to simulate pathogen evolution under diverse selective pressures. The phylogenetic model is incorporated within an agent-based model (ABM) of multi-strain epidemic transmission and control (detailed in Section Multi-strain transmission model), and consists of five key components:

- Genome structure represented by a nucleotide sequence with positions assigned to different functions.
- Point mutations occurring at a rate that is averaged across the genome.
- Pathogen fitness determined based on amino acids at specific regions of the genome.
- Infected host categories, differentiating between typical infected hosts and chronically infected hosts.
- Within-host (or intra-host) selective pressure generating advantageous pathogen mutations.

These components capture complex evolutionary dynamics relating selective pressures, mutations, and varying levels of immune response emerging in the heterogeneous host population facing various disease scenarios.

In this study, we calibrated the model to match viral characteristics and epidemic dynamics of SARS-CoV-2, using the COVID-19 pandemic as a case study. This model can be adapted to explore the evolutionary dynamics of other pathogens causing communicable diseases.

## Genome structure

Each infected agent (i.e., host) is assigned an artificially constructed genome. Importantly, the simulated genome is designed to model evolutionary dynamics but is *not* mapped to real features of the viral genome such as known coding sequences, epitopes, or viral functions. Furthermore, for reasons of computational efficiency, we simulate a sequence of only 3,090 nucleotide positions, approximately 10% of the true size of the viral genome [1]. For simplicity, we model only one viral genome per host at any given time step, i.e., we assume that a single viral population dominates in the host, and there is a narrow transmission bottleneck [2].

Each nucleotide position in the simulated genome is represented by a number between 0 and 3. Every three nucleotides is interpreted as a codon (3,090 nucleotides correspond to 1,030 codons, see S1 Fig), encoding a sequence of amino acids following the standard translation rules [3]. We annotate the 20 known amino acids by numerical labels from 0 to 19 instead of the conventional letters [3]. Although we model translation to an amino acid sequence, we do not look for coding regions or otherwise interpret or annotate the simulated sequence in the context of the real virus.

The simulated genome is partitioned into two distinct regions (S1 Fig): the “spike” region (consisting of 100 codons) and the “non-spike” region (consisting of 930 codons). This partitioning affects only two aspects of the simulation: (1) vaccine-related selective pressure is restricted to the spike region, and (2) mutations in the spike region have a greater chance of enhancing transmissibility (Section Contribution to fitness). 75 codons

(45 codons in the spike region and 30 codons in the non-spike region) are interpreted as epitopes relevant for modelling of immunity [4, 5]. 1,000 codons (including 45 that are also in epitopes) are interpreted as relevant to transmissibility. Section Incorporating compound immunity in infection probability provides more details.

## Mutations

In this study, we model mutations as random substitutions at single nucleotide positions. We do not consider insertions, deletions, recombination, or other structural changes to the genome. Fitness and antigenicity are computed from the amino acid sequence, meaning that synonymous nucleotide substitutions will not result in a functionally different simulated genome. We also assume that the transmissibility and antigenicity of viral variants can be computed based on independent contributions from each mutation with respect to the ancestral strain.

In each half-day simulation cycle, the total number of mutations ($L$) in each of the viral genomes within infected hosts is sampled from a Poisson distribution. The mean of the distribution is set based on an average mutation rate of 0.001 per nt per year across the genome, i.e., $3090 \times 0.001/(365 \times 2) \approx 0.00423$. The average mutation rate was chosen within the reported range for SARS-CoV-2 of 0.000219 to 0.0012 per nt per year [2, 6]. The relationship between mutations and their contribution to fitness is described in Section Contribution to fitness.

## Contribution to fitness

Individual mutations in the genome affect the overall viral fitness, resulting in growing transmissibility and higher reproductive number [7–9]. [7] assessed the relative fitness of SARS-CoV-2 lineages (measured as the fold increase in $R$ over the ancestral strain) by linearly combining fitness contributions of individual amino acid substitutions, some of which were found to be fitness-increasing and recognised as spike mutations and non-spike mutations within the nucleocapsid and nonstructural proteins. Additionally, [9] identified a general distribution of amino acid selection at each position in SARS-CoV-2 sequences and highlighted positions that are prone to viral escape and mutations.

Informed by these studies, we employ a weight table to quantify the individual contributions to fitness of amino acids at each codon position. The overall fitness of a strain, determined by its genome, is modelled as the sum of these individual contributions. This approach allows us to determine fitness of any simulated genome with arbitrary mutations. Specifically, the table assigns a weight $a_{i,j}$ for each of the $N = 1,030$ codon positions and $N_A = 20$ types of amino acids. For the COVID-19 case study, we assigned the weights as follows:

- sample $\mathcal{N}(0, 0.085)$ for $i \in [0, 99]$, the spike region,
- 0 for $i \in [100, 129]$, the non-spike epitopes, and
- sample $\mathcal{N}(0, 0.07)$ for $i \in [130, 1029]$, the non-spike region.

Here, we chose a higher-variance distribution for the weights in the spike region so that those codons have a higher probability of contributing to the viral fitness relative to the non-spike region. This is based on the observation of a higher substitution rate in the spike region compared to other positions [10, 11].

S2 Fig shows a section of the weight table (the first 26 codon positions). The weight table generated for a simulation regulates the pathogen's evolution. In the absence of other pressures, one would expect substitutions that increase fitness to be preferentially fixed as a simulation progresses, leading to a gradual increase in transmissibility. Using the weight table, we calculate the overall fitness $K$ of a genome $s$ by combining the individual contributions of the present amino acids:

$$K(s) = \sum_{i=0}^{N-1} a_{i,s(i)} \tag{7}$$

where $a_{i,s(i)}$ is the fitness contribution of amino acid $j = s(i)$ located at codon position $i$, with $j \in [0, N_A - 1]$. We note that $a_{i,s(i)}$ can be zero, positive, or negative, representing mutations of neutral, advantageous, or disadvantageous contribution to pathogen fitness. We note that Eq 7 and Eq 1 in the main manuscript are equivalent.

Prior to simulating the COVID-19 case study, we generated a genome for the ancestral SARS-CoV-2 strain sampled such that its viral fitness, calculated using Eq 7 and the weight table, fell between 2.65 and 2.85, matching the transmissibility range of the ancestral SARS-CoV-2 strain modelled in our previous work [12]. We used this ancestral genome as a starting point from which other variants originate, thus assuming a shared ancestry for all variants [13, 14]. To optimise memory usage, given the high number of simulated infections, each strain is represented by a list of mutations against the ancestral genome instead of a full-length genome.

## Infected host categories

Host factors, including individual variation in immune response, are known to strongly affect epidemic progression [15, 16]. In particular, chronic carriage of SARS-CoV-2 by immunocompromised hosts is thought to have had a key role in the emergence of new viral variants during the pandemic [2]. In a host with compromised or suppressed immune function, the virus is subjected to different selective pressures and within-host dynamics. A host immune system that is not able to effectively clear the virus is likely to result in a longer infection duration, a higher viral load within the host, and a greater diversity of viral sub-populations [2]. These conditions may facilitate more substitutions and larger increases in viral fitness (saltational evolution) compared to the evolution of the virus over the same interval of time when transmitted by a chain of typical hosts and subjected to transmission bottlenecks and competent immune systems. In the case of SARS-CoV-2, chronically infected hosts can have a prolonged recovery period of several months, compared to one to two weeks for typical infected hosts [10, 17].

To examine the possible role of chronic infections on the pathogen evolution, we differentiate between two infected host categories: chronically infected hosts and the typical infected hosts. The main distinctions include the following aspects: (i) chronically infected hosts are assigned a different natural history model with a significantly longer recovery period, randomly sampled from a uniform distribution ranging from 60 to 370 days reported in many studies (S3 Fig) [10, 18, 19], and (ii) chronically infected hosts may have a higher intra-host selective pressure (detailed in Section Sensitivity analysis and S20 Fig).

Prior study [10] reported that chronically infected hosts make up a small fraction of the infected hosts (between 0.1 and 0.5% of COVID-19 infections), and only 32% of these hosts may experience strong positive intra-host selection. Considering the high

attack rate of the COVID-19, we assume that 0.1% of the entire population is susceptible to chronic infection with COVID-19. We also assume a stronger within-host selective pressure in chronically infected agents, compared to typical infected hosts.

To evaluate the model robustness and quantify the impact of the chronically infected hosts on the overall phylodynamics, we performed sensitivity analysis varying the percentage of chronically infected hosts between 0% to 5% (see Section Sensitivity analysis, and S19 Fig).

## Intra-host (within-host) selective pressure

The emergence of new variants requires mutations with a fitness advantage capable of breaking through the transmission bottleneck [2]. Mutant strains require time to replicate and out-compete other viral sub-populations within the host in order to be transmitted onwards to other hosts. The selection dynamics within the host are influenced by host characteristics and the length of infection [2, 10, 18].

A virus that has less opportunity to develop within-host diversity will be less effective at exploring the fitness landscape. We emulate this by biasing the fitness of mutant sequences generated from a parent sequence. At each time step where we need to generate mutations in the viral sequence associated with an infected host, we first independently generate $M$ candidate sequences and rank them by their fitness. We then sample one sequence from the top $X \leq M$ candidates to be the new sequence. The difference $M - X + 1$ specifies the selective pressure strength. For example, if $M = X$, this is equivalent to generating only a single candidate sequence, indicating a weak selective pressure. On the other hand, if $M = 100$ and $X = 1$, then the selected sequence will be the fittest of 100 randomly generated candidate sequences, indicating a strong selective pressure and bias towards higher fitness. In other words, small values of $X$ imply more efficient exploration of the fitness landscape as might be expected during a chronic infection. This mechanism emulates within-host dynamics that are not explicitly modelled, since each infected agent is associated with only a single viral genome at each simulation cycle. We discussed this distinction in Section Discussion.

Each cycle, for every infected host agent carrying genome $s$ and acquiring $L$ independent mutations (governed by the mutation rate as described in Section Mutations), we simulate the within-host selective pressure through a three-step procedure:

1. Randomly generate $M - X + 1$ mutated genome sequences by independently selecting and changing $L$ positions in sequence $s$ for each, by substituting the corresponding nucleotide number by a uniformly sampled number between 0 and 3. These generated mutated genome sequences become candidates for the upcoming selection process.

2. Compute the fitness of each of the $M - X + 1$ generated mutated genome sequences using the fitness weight table (see Section Contribution to fitness). Rank these generated mutated genome sequences by fitness in descending order.

3. Select the genome sequence with the highest rank (fittest) among the generated mutated genome sequences. Replace $s$ by this selected sequence.

At the start of their infection, both typical infected hosts and chronically infected hosts are processed using the described selective pressure procedure with the same selective strength $X$ where $X \lessapprox M$ (representing low selective pressure). However,

beginning from 60 days post-infection, we differentiate chronically infected hosts by assigning them a higher selective strength (i.e., $X \ll M$). In comparison, by this point typical infected hosts have already recovered. This setup aligns with the observation that a 60-day post-infection interval is observed before the intra-host pressure begins to impact the SARS-CoV-2 transmission bottleneck significantly [2, 10]. We note that chronically infected hosts have a lengthened recovery period between 60 and 370 days (see Section Infected host categories), and the described selection process continues until the host recovers.

## (B) Artificial agent-based population

Prior to the simulation, we stochastically generated three distinct artificial populations of anonymous agents (corresponding to layer (B) Demographic, shown in Fig 12), used to examine evolutionary and epidemiological dynamics:

- (large) approx. 25.4 million, comparable to the Australian population;
- (medium) approx. 8 million, comparable to the population of New South Wales (a relatively population-dense state in Australia);
- (small) approx. 1.7 million, comparable to the population of South Australia (a relatively population-sparse state in Australia).

We selected these population sizes for two reasons: (i) to investigate the relationship between the population size and phylodynamics, and (ii) to ensure computational feasibility. These considerations are based on the observation that the SARS-CoV-2 variants of concern may have emerged in countries of different population sizes (e.g., South Africa, with a population of approximately 59 million; UK, with a population of around 56 million; and Botswana, with a population over 2.4 million). In addition, our preliminary test simulations revealed that the computational cost increases significantly with the population size (see Section Computational complexity and implementation). In this study, we focus on revealing the representative evolutionary characteristics of SARS-CoV-2, while maintaining computational efficiency by using the three populations listed above. However, given sufficient computational resources, much larger populations (e.g., over one billion population representing countries such as India) may also be generated in order to quantify the impact of population size on phylodynamic and disease dynamics.

The constructed artificial population captures essential demographic characteristics and commuting patterns represented in the latest Australian census and other datasets, as well as agent-to-agent interactions in various social mixing contexts, thus representing the population heterogeneity. Specifically, we generated the artificial population using demographic and travel data sourced from the Australian Bureau of Statistics (ABS) 2021 Census [20], international air traffic reports (detailing incoming passenger flows at Australian airports) [21], and educational registration records (including data on schools and students) [22]. Each agent in the artificial population was assigned multiple demographic attributes, such as age, gender, and residency location, alongside social mixing contexts across various settings: residential (e.g., household, household cluster, neighbourhood and statistical area which maps to a local government area), educational (e.g., classroom/school for agents aged 18 years or younger), and workplace (for agents aged over 18 years). Fig 5 provides a visual representation of the considered social mixing contexts. For each agent, the residential contexts are determined based on residential demographics, while the workplace and

educational contexts are assigned based on commuting patterns (commute to work and class/school, depending on the agent's age group).

The population generation used by PhASE TraCE can also cover a specific geographical area. In other words, in addition to an artificial population matching the demographics of the entire Australian population, it is possible to generate smaller populations corresponding to individual states and territories, while maintaining their demographic attributes and travel patterns. The population generation algorithm resolves several known discrepancies (introduced by privacy-protection algorithms employed by government agencies), and maintains integrity across different public datasets [23, 24]. A detailed description of the population generation methodology and the population data structure can be found in the Supplementary Materials of a prior study [24] and the user guide of our open-source software [25].

# (C) Multi-strain transmission and control

## Multi-strain transmission model

The multi-strain transmission model of PhASE TraCE stochastically simulates pathogen transmission between hosts (Epidemic Layer shown in Fig 12). This model introduced several new features to our previous ABM, which was implemented in AMTraC-19 [12, 24, 26, 27]. In this section, we describe how transmission is affected by the phylogenetic model described above.

At the start of the simulation, all agents in the population are susceptible. Initial infections carrying the generated ancestral genome are seeded in metropolitan statistical areas (around international airports) as imported cases. These seeding events occur periodically throughout the simulation. The viral genome of the imported infections is taken to be the variant with the highest transmissibility from the preceding month of simulation, with additional mutations randomly added (using a weak selective pressure, see S4 Table) to represent the evolution of the virus outside of the simulated population.

From the initial infections, the pathogen propagates through the population as susceptible and infected agents interact. These interactions occur in various social contexts in discrete half-day time cycles. During "daytime" cycles, agents interact in workplaces or educational settings (e.g., class, grade, school). "Nighttime" cycles instead involve interactions in residential settings (e.g., household, household cluster, neighbourhood, and community). Weekdays consist of a daytime cycle and a nighttime cycle, whereas weekend days consist of two nighttime cycles (i.e., no interactions in workplace/educational contexts).

A transmission event changes the health state of the newly-infected agent from Susceptible to Infectious (asymptomatic or symptomatic). Once the infection is cleared, the agent transitions to the Recovered state. Recovered agents are again susceptible to re-infection, but any subsequent transition back to Infectious is moderated by the immunity level (see following sections for more detail).

### Susceptible-infectious transition

Transmission from agent $j$ to agent $i$ depends on the probability of their interaction, their ages, and the context $g$ in which the agents interact. These daily context- and

age-dependent interaction probabilities $q_{j\to i}(g)$ have been defined and calibrated in previous studies [12, 27]. At cycle $n$, the transmission probability, $p_{j\to i}(n, g)$, is determined as follows:

$$p_{j\to i}(n, g) = K(s_j)\ f_j(n - n_j)\ q_{j\to i}(g) \tag{8}$$

where $K(s_j)$ represents the transmissibility of pathogen variant $s_j$, proportional to the corresponding basic of effective reproductive number, i.e., $K(s_j)$ is the fitness of genome $s_j$ carried by agent $j$, as defined by Eq 7; $n_j$ is the time cycle when agent $j$ started experiencing the onset of infection; and the function $f_j(n - n_j)$ determines agent $j$'s infectivity over time according to the natural history model. At the time cycle $n$, agent $j$'s infectivity is $0 < f_j(n - n_j) \leq 1$ with the infectivity peak equal to 1. For uninfected agents, $f(\cdot) = 0$. For simplicity, we assume that all variants share the same progression of disease, but differ in their transmissibility (determined by fitness $K$), and that only one variant can be transmitted during the infection process. We note that Eq 8 and Eq 3 in the main manuscript are equivalent.

We then extend the scenario to consider the probability of agent $i$ getting infected by interacting with all other agents sharing the same social mixing contexts across all levels at time cycle $n$, defined as follows:

$$p_i(n) = 1 - \prod_{g \in G_i(n)} \prod_{j \in A_g \backslash \{i\}} (1 - p_{j\to i}(n, g)) \tag{9}$$

where $G_i(n)$ is the set of social mixing contexts where interactions between agent $i$ and other agents may occur (depending on factors such as weekday or weekend, daytime or nighttime), and $A_g\backslash\{i\}$ is the list of all agents in the context $g \in G_i(n)$ except agent $i$. We shall expand Eq 9 in later sections to incorporate the effects of non-pharmaceutical interventions (Eq 11) and effects of immunity (Eq 20). We note that Eq 9 and Eq 2 in the main manuscript are equivalent.

Eq 9 defines the infection probability for agent $i$ interacting with other infected agents across all of its social contexts. However, this probability is an aggregate of the interactions and does not identify the specific source of infection, which must be known in order to copy its viral sequence to the newly infected host. We assign the source of infection by constructing a discrete distribution to randomly sample one agent from all potential infected agents $j$ across all social contexts $g \in G_i(n)$ to which agent $i$ belongs. The probability of selecting agent $j$ is given by

$$\frac{p_{j\to i}(.)}{\sum_j p_{j\to i}(.)}.$$

An infectious agent is either symptomatic or asymptomatic. The probability of being symptomatic, $z_i(n)$, depends on the infection probability $p_i(n)$ and an age-dependent scaling factor $\sigma_i$ characterising the proportion of symptomatic cases among all infections in the corresponding age group:

$$z_i(n) = \sigma_i\ p_i(n) \tag{10}$$

where $\sigma_i = \sigma^a$ for adults (age $> 18$) and $\sigma_i = \sigma^c$ for children (age $\leq 18$). The asymptomatic infectious agents have lower infectivity (i.e., lower $f_i(n - n_i)$, compared to the symptomatic agents, which also affects the strength of transmission to other susceptible agents.

Following our prior studies [12, 24, 27], we assumed that only a fraction of total infections, especially asymptomatic infections, are detected daily. In our model, we set a lower detection probability for asymptomatic cases ($\pi'$) than the symptomatic cases ($\pi$), such that $\pi \gg \pi'$. In addition, we assumed that chronically infected cases have the same detection probability as asymptomatic cases. In the COVID-19 case study, we used detection rates of $\pi = 0.13$ and $\pi' = 0.01$, calibrated to the Omicron variant of SARS-CoV-2 [28].

### Infectious-recovered and recovered-susceptible transitions

Following the natural history model, an infectious agent recovers after a certain recovery period dependent on the host category (see Section Sensitivity analysis, and S19 Fig). We assume that both typical and chronically infected hosts develop infectivity fairly quickly from the onset of infection (following a lognormal distribution). However, chronically infected hosts experience a significantly longer recovery than the typical infected hosts. The natural history model describing the disease progression for both host categories is illustrated in S4 Fig.

For a period of 60 days following recovery, agents are immune to re-infection, consistent with empirical definitions that attempted to distinguish between reinfection and serial testing of an initial infection for SARS-CoV-2 [29, 30]. After 60 days, the immunity induced by infection starts to wane, and the recovered agent becomes susceptible again. The re-infection probability for a recovered agent on subsequent exposure depends on their infection and vaccination history both in terms of the genomic similarity of viral strains and the time elapsed (see Section Vaccination for more details).

## Non-pharmaceutical interventions

The model includes several non-pharmaceutical interventions (NPIs), such as case isolation (CI), home quarantine (HQ), school closures (SC), and social distancing (SD). The implementation of each NPI is governed by: (i) a macro-distancing parameter determining the population fraction adhering to the intervention (S2 Table), and (ii) a set of micro-distancing parameters that quantify the altered (typically decreased) interaction strengths between NPI-compliant individuals in a given social context (see S1 Table).

Following Eq 9, the NPI-affected infection probability for agent $i$ without prior infections or vaccinations is given by

$$p_i(n) = 1 - \prod_{g \in G_i(n)} \left[ 1 - F_g(i) \left( 1 - \prod_{j \in A_g \setminus \{i\}} \left(1 - F_g(j)\ p_{j \to i}(n, g)\right) \right) \right] \tag{11}$$

where $F_g(j)$ denotes the NPI-affected strength of interaction between agent $j$ and other agents in mixing context $g$. For an agent $j$ adopting NPIs, $F_g(j) \neq 1$, denoting a modified infection probability from agent $j$. For an agent $j$ not adopting any NPIs, $F_g(j) = 1$.

The assignment of NPI-compliant agents is determined based on a Bernoulli process with the probability specified by the macro-distancing parameters. While an agent may comply with multiple NPIs, their interaction strengths, $F_g(j)$, can only be adjusted to one NPI. We therefore use the parameters for only the first NPI to which an agent complies in the following ordered list: CI, HQ, SD, and SC. The micro- and

macro-distancing parameters of CI vary depending on the host type, where chronically infected hosts are fully compliant (i.e., macro-distancing level is set to 1.0), with significantly reduced interaction strengths across all social contexts (see S1 Table and S2 Table). This parametrisation agrees with the public health recommendations for chronically infected hosts to take extra precautions to prevent severe illness [31].

We define SD as a broad behavioural-driven NPI that reduces interaction strengths among individuals, due to stay-at-home orders and other measures (including physical distancing, mask wearing, etc.) adopted during (partial) lockdowns. In other words, the SD compliance level can be interpreted as the fraction of the population that follow the restrictions imposed during (partial) lockdowns. We designed an intervention scenario with a dynamically adjustable SD compliance profile representative of NPIs implemented in many countries during the COVID-19 pandemic [27]. Specifically, we set the SD compliance level to gradually decline annually over the 6-year simulation period, from 50% at the start of the pandemic in 2020 to 20% in the endemic stage from 2023 onwards. In addition, the activation of SD was set to be triggered by a sufficiently high prevalence (e.g., exceeding 200 cases), with SD deactivated once the disease prevalence falls below a certain threshold (e.g., 100 cases). In our simulations, the deactivation of SD has not been triggered. Fig 5 describes a detailed setup and parameterization.

# (D) Immuno-epidemiological model

## Vaccination

Following our prior work [24, 27, 32, 33], the model includes a vaccination scheme under which agents are immunised according to an average daily vaccination rate. The vaccination rate is demographically stratified by age, with agents aged 18 to 35 years old vaccinated at a 10-fold higher rate than young or elderly agents, and by immunisation history, with 60% of vaccines going to agents with some history of vaccination [34]. Agents are eligible for vaccination only if they are free from infection for at least three months. We did not model vaccination schedules consisting of multiple doses within a season, instead assuming that a single vaccination event confers the cumulative immunity of the entire recommended dosing regimen in a year.

Vaccinated agents have diminishing levels of protection due to two factors: (i) vaccine escape as circulating variants diverge genetically from the vaccine strain at specific epitopes, and (ii) immunity from vaccination wanes over time. The vaccine used each year in the simulation is chosen to target the dominant variant circulating in the preceding simulated year (starting from 2021). We model only one vaccine type administered at any one time. We assumed that peak immunity occurs immediately after vaccination [35].

We simulated a mass vaccination roll-out with a variable daily vaccination rate updated annually, starting from zero (i.e., no available vaccine) in 2020, rising to 0.147% of the entire population per day in 2021 (when vaccines first became available), and then linearly declining to 0.047% of the population per day in 2026 (S5 Fig).

## Compound immunity and waning effects

Individuals who have been vaccinated and/or experienced prior infections develop immunity against the disease, subsequently reducing their immediate susceptibility to infection [36]. Immunity resulting from both vaccination and natural infection in the

same individual, known as hybrid immunity, offers more robust protection compared to immunity derived solely from vaccination or natural infection. Hybrid immunity against SARS-CoV-2, for example, is found to have stronger and longer-lasting immune responses than the immunity granted from vaccination or infection alone [37, 38]. As described in Methods (subsection Immunological layer (D)), we use the term "compound immunity" to encompass immunity due to previous exposures, vaccination, and hybrid immunity [28].

For each agent $i$ with a history $H_i$ containing all vaccination and past infection records $r \in H_i$, we characterise their immunity by two distinct components: (i) compound immunity against symptomatic infection ($M_i^c$, described in section Compound immunity against symptomatic infection), and (ii) compound immunity against forward transmission ($M_i^\iota$, described in section Compound immunity against forward transmission). We then integrate the compound immunity into the infection probability in Section Incorporating compound immunity in infection probability. These immunity components range between 0 and 1 with zero indicating no immunity and one indicating full immunity.

### Compound immunity against symptomatic infection

To determine the immunity against symptomatic infection of a susceptible agent $i$, exposed to a potential infection from an infectious agent $x$, we need to consider the genome sequence of the variant $s_x$ carried by $x$. At time cycle $n$, given a single vaccination or infection record $r$ for agent $i$, we determine its immunity against symptomatic infection as follows:

$$m_i^{\mathrm{c}}(n, r, s_x) = m^{\mathrm{c}}(r)\Big[1 - \min\Big(1, \epsilon^c(n - n_r)\Big)\Big]\Big[1 - \min\Big(1, \tau\Delta_r(s_x, s_T)\Big)\Big] \tag{12}$$

where

- $m^{\mathrm{c}}(r)$ denotes the peak immunity against symptomatic infection developed from either vaccination or past infection recorded in $r$ against a target variant with genome $s_T$ (see S3 Table),
- $\epsilon^c$ is the waning rate of immunity (proportion of immunity lost) against symptomatic infection per simulation cycle,
- $(n - n_r)$ is the number of cycles between the current cycle $n$ and the record cycle $n_r$,
- $\Delta_r(s_x, s_T)$ is the genetic distance measured by number of amino acids that differ between genomes $s_T$ and $s_x$ in the epitope regions, either in spike regions only [39, 40] (if $r$ is a vaccination event), or in both spike and non-spike regions (if $r$ is a previous infection) [41],
- factor $\tau$ is the immunity reduction per amino acid difference between these two genomes.

Eq 12 shows that $m_i^{\mathrm{c}}$ reduces linearly over time, governed by two constant rates: immunity waning rate $\epsilon^c$ over time, and immunity reduction factor $\tau$ over genetic distance. This assumption matches observations reported in various studies suggesting a linear reduction between (i) COVID-19 vaccine effectiveness and time [42–44], and (ii) genetic distance and immune evasion [35]. S6 Fig shows a linear regression fitting of vaccine effectiveness over time for different types of vaccines, constructed using

supplementary data from [44]. We note that the waning immunity term can be easily adjusted to follow a non-linear reduction, such as exponential decay [45, 46] or based on a gamma distribution [47].

We then compute the compound immunity for agent $i$ induced by multiple vaccination and/or infection events. The compound immunity against symptomatic infection, $M_i^c(n, H_i, s_x)$, accumulates non-linearly with an upper bound of 1 (i.e., perfect immunity):

$$M_i^c(n, H_i, s_x) = \min\left(\sqrt{\sum_{r \in H_i} \left[m_i^c(n, r, s_x)\right]^2}, 1\right) \tag{13}$$

We note that this equation is equivalent to Eq 4 in the main manuscript.

In our model, the level of protection provided by $M_i^c$ is further decomposed into two components: the immunity against susceptibility ($M_i^\theta$), and the immunity against symptomatic infection given the infection ($M_i^\zeta$). These two components may also be referred to as the susceptibility-reducing immunity and disease-preventing immunity, respectively. Following prior studies [26, 27, 32], we calculate $M_i^\theta$ and $M_i^\zeta$ as:

$$M_i^\zeta(n, H_i, s_x) = M_i^\theta(n, H_i, s_x) = 1 - \sqrt{1 - M_i^c(n, H_i, s_x)} \tag{14}$$

which satisfies a general relationship:

$$M_i^c(n, H_i, s_x) = M_i^\zeta(n, H_i, s_x) + M_i^\theta(n, H_i, s_x) - M_i^\zeta(n, H_i, s_x) M_i^\theta(n, H_i, s_x) \tag{15}$$

We note that Eq 15 reduces to Eq 14 when $M_i^\zeta(n, H_i, s_x) = M_i^\theta(n, H_i, s_x)$.

### Compound immunity against forward transmission

Following [32, 33, 48], we also consider the immunity component against forward transmission. At time cycle $n$, for an infected agent $j$, the immunity against forward infection from one vaccination/infection event, represented by record $r$, is calculated as follows:

$$m_j^\iota(n, r) = m^\iota(r)\Big[1 - \min\Big(1, \epsilon^\iota(n - n_r)\Big)\Big] \tag{16}$$

where $m^\iota(r)$ denotes the peak immunity against forward transmission developed from either vaccination or past infection recorded in $r$; the rate $\epsilon^\iota$ is the forward-transmission immunity waning rate per simulation cycle; and $n_r$ is the record cycle of $r$. We assume that $m_j^\iota(n, r)$ is dependent only on the immunological history of the infection source agent $j$, and is independent of the immunological history of the potentially infected agent.

We then consider the compound immunity against forward transmission, accounting for all records in $H_j$, which follows a non-linear combination of all individual components $m_j^\iota(n, r)$. Similar to the calculation of the compound immunity against symptomatic infection, we calculate the compound immunity against forward transmission where the upper bound equals 1 (i.e., perfect immunity):

$$M_j^\iota(n, H_j) = \min\left(\sqrt{\sum_{r \in H_j} \left[m_j^\iota(n, r)\right]^2}, 1\right) \tag{17}$$

### Incorporating compound immunity in infection probability

Here, we incorporate the compound immunity, against infection ($M^{\theta}$) and forward transmission ($M^{\iota}$), within the infection probability, extending the NPI-affected probability of infection (Eq 11 in Section Non-pharmaceutical interventions).

The impact of compound immunity on infection probability is modelled by distinguishing two factors that affect the infection of a susceptible agent $i$:

($\mathcal{P}^{\mathcal{I}}$) the impact of forward infection from all infected agents $j$ with whom this agent has contact, affected by $M^{\iota}_j$, and

($\mathcal{P}^{\mathcal{II}}$) the agent $i$'s immunity against infection from these sources, affected by $M^{\theta}_i$.

We modelled the impact of factors ($\mathcal{P}^{I}$) and ($\mathcal{P}^{II}$) by decomposing the transition from Susceptible state to Infectious state into two consecutive steps:

(1) considering factor ($\mathcal{P}^{I}$) alone, the model identifies whether agent $i$ is *potentially* infected, and if so, it determines the most likely source of this potential infection; and

(2) considering ($\mathcal{P}^{II}$), the model ascertains whether infection is *actually* transmitted from this source to agent $i$.

**Step (I).** We calculate the probability of agent $i$ becoming *potentially* infected at time cycle $n$, $p^{I}_i(n)$, while accounting for NPI-compliance and the immunity against forward infection of other agents, i.e., quantifying factor ($\mathcal{P}^{I}$), as:

$$p^{I}_i(n) = 1 - \prod_{g \in G_i(n)} \left[ 1 - F_g(i) \left( 1 - \prod_{j \in A_g \setminus \{i\}} \left( 1 - \left(1 - M^{\iota}_j(n, H_j)\right) \, F_g(j) \; p_{j \to i}(n, g) \right) \right) \right] \tag{18}$$

where $F_g(j)$ denotes the strength of interaction between agent $j$ and other agents in mixing context $g$, and $M^{\iota}_j(n, H_j)$ represents the immunity against forward transmission based on the history $H_j$ of past infections and vaccinations of agent $j$. We note that $p^{I}_i(n)$ accounts for the immunity against forward transmission developed by any agent $j$ sharing a social context with agent $i$, thus accounting for factor ($\mathcal{P}^{I}$).

If agent $i$ is determined to be *potentially* infected according to the Bernoulli trial with $p^{I}_i(n)$, the source potentially infecting agent $i$ is identified by sampling from a discrete distribution that includes all infectious agents $j$ sharing a social context $g$ with agent $i$. The probability of selecting an agent $j$ as a potential source of infection from this distribution is given by

$$\frac{\left(1 - M^{\iota}_j(n, H_j)\right) \; F_g(j) \; p_{j \to i}(n, g)}{\sum_j \left(1 - M^{\iota}_j(n, H_j)\right) \; F_g(j) \; p_{j \to i}(n, g)} \, .$$

**Step (II).** Given the identified source agent $e$ potentially transmitting infection to agent $i$, the model identifies the corresponding genome $s_e$, yielding the probability of infection transmission from source $e$ to agent $i$:

$$p^{II}_i(n, H_i, s_e) = 1 - M^{\theta}_i(n, H_i, s_e) \tag{19}$$

where $M^{\theta}_i(n, H_i, s_e)$ is the susceptibility-reducing immunity of agent $i$ with immunological history $H_i$, determined with respect to genome $s_e$ specifically.

Combining two steps (I) and (II) determines the infection probability for susceptible agent $i$ (i.e., the probability of its transition from Susceptible to Infectious state), in the context of relevant NPI compliance and immunological histories within the population:

$$p_i(n, H_i, s_e) = p_i^{II}(n, H_i, s_e)\, p_i^{I}(n) \tag{20}$$

The probability that susceptible agent $i$ becomes ill (i.e., symptomatic), originally defined in Eq 10, is then updated as:

$$z_i(n, H_i, s_e) = \Big(1 - M_i^{\zeta}(n, H_i, s_e)\Big)\, \sigma_i\ p_i(n, H_i, s_e) \tag{21}$$

where $M_i^{\zeta}$ is the disease-preventing immunity of agent $i$, developed as a result of past infections and vaccinations (represented in $H_i$), determined against the specific source of infection (i.e., the variant characterised by $s_e$).

S3 Table lists key immunity-related parameters used in the COVID-19 case study. Using a range of reports [49–51], we assumed that the peak level of vaccine-induced immunity against SARS-CoV-2 infection is comparable to that of natural immunity.

## Parametrisation and additional results

The phylogenetic parameters used in the SARS-CoV-2 case study are summarised in S4 Table. For an in-depth description of the dynamics simulated with these parameters, refer to Section Case study: Rapid punctuated evolution of SARS-CoV-2. Additionally, see Section (A) Phylogenetic model for parameter justification and calibration.

S8 Fig to S11 Fig illustrate simulated epidemic dynamics, as well as the dynamics of transmissibility and mutation accumulation across populations of varying sizes: small (1.7 million), medium (8.0 million), and large (25.4 million). These figures provide insights into population health states, infection and immunisation histories, and viral transmissibility trends, aligned with phylogenetic dynamics observed in different realisations across diverse population scenarios.

S13 Fig to S15 Fig present pairwise Hamming distance dynamics generated using identical phylogenetic parameters across three different population sets. S13 Fig shows alternative evolution dynamics relative to those shown in Fig 9A. The high variability of genomic diversity dynamics across different realisations results from the stochastic emergence of variants; hence, we refrain from averaging genomic diversity dynamics across realisations. Nonetheless, the pattern of genomic diversity dynamics aligns well with the trends discussed in Section Case study: Rapid punctuated evolution of SARS-CoV-2, which includes periods of drift, rapid rise, and abrupt collapse. To assess the stationarity of genomic diversity dynamics, we performed the Augmented Dickey–Fuller test. S17 Fig shows that the p-value for stationarity associated with genomic diversity dynamics varies significantly with population size, highlighting the increased difficulty for variants to spread and become dominant in larger populations.

## Counterfactual modelling

In this section, we describe two counterfactual modelling scenarios of SARS-CoV-2: the impact of chronic infections on the SARS-CoV-2 evolution (Section Chronic infections), and the impact of population size on stationarity of the genomic diversity $\overline{D}$ (Section Stationarity of genomic diversity).

### Chronic infections

S16 Fig differentiates between the COVID-19 pandemic simulation scenarios with and without chronic infections. Notably, the absence of chronic infections results in a reduced number of recurrent incidence waves (S16 Fig, panel A) and significantly reduced fluctuations in the genomic diversity $\overline{D}$, producing lower variability (i.e., a smaller standard deviation) across realisations (S16 Fig, panel D). Furthermore, the growth of both transmissibility (i.e., fitness $K$) and the accumulated mutations $\widehat{D}$ is significantly slower (S16 Fig, panels B and C), impeding the emergence of high-fitness variants.

We also explored whether the pathogen phylodynamics are correlated with (1) fractions of chronic infection, and (2) strength of the within-host selective pressure in chronically infected hosts. S19 Fig shows that a hundred-fold increase in the chronic infection fraction (e.g., from 0.05% to 5% of the population) yields only negligible impact on the incidence curves and phylodynamics. This indicates a ceiling effect of the chronic infection fraction on the incidence, fitness, and accumulated mutations. In contrast, S20 Fig presents a clear positive correlation between the within-host selective pressure and the phylodynamic characteristics. That is, a higher selective pressure (i.e., selecting top 10% of mutated genome candidates out of their ranked list, $X = 10$, $M = 100$) leads to higher incidence peaks and a more rapid increase in the pathogen fitness and accumulated mutations.

### Stationarity of genomic diversity

The genomic diversity traced in empirical data from 2020 to 2024 shows fluctuating yet relatively stationary dynamics, without notable trends of increasing or decreasing diversity, as illustrated in Fig 2C). The Augmented Dicky-Fuller (ADF) test (detailed in Section Materials and methods) applied for the empirical genomic diversity produced p-value of 0.024, indicating stationary at the significance level of 0.05.

However, stationarity of the simulated genomic diversity is harder to establish for larger population sizes, as illustrated in S17 Fig. For a small population of 1.7 million, the genomic diversity dynamics are closer to stationary (p-value of 0.078). This outcome is robust to variations of the fitness contributions weight table, as well as changes in the within-host selective pressure, as shown in S18 Fig. For larger population sizes, the ADF tests produced larger p-values of 0.317 (8 million) and 0.730 (25.4 million), indicating a progressive loss of stationarity.

## Sensitivity analysis

We performed sensitivity analysis by tracing the output variables of interest in response to changes in one input variable while keeping the other inputs specified at default values. In this study, we have four output variables: incidence, transmissibility fitness, accumulated mutations, and genomic diversity. S19 Fig and S20 Fig show simulated dynamics of the output variables of interest by varying two input parameters: the fraction of chronically infected hosts and the within-host selective pressure in chronically infected hosts, respectively.

## Computational complexity and implementation

The multi-scale phylodynamic simulator, PhASE TraCE, builds upon our open-source agent-based epidemiological simulator, AMTRaC-19 [25], written in C++. The architecture of PhASE TraCE includes four layers (Fig 12), with the immunological and phylogenetic layers being novel additions, which significantly extend the capabilities of AMTRaC-19. PhASE TraCE is designed to simulate the dynamic interaction between pathogen transmission and evolution over a prolonged timeframe over multiple years (unlike AMTRaC-19 which was typically used to simulate 6-9 months of a pandemic). The new modelling capabilities and simulation requirements increase the computational complexity, presenting four specific computational challenges:

- *Longer simulation timeframe* (typically, over 6 years or 2,000 simulation days). This is needed to trace the medium-term evolutionary dynamics of pathogens. State-of-the-practice simulators typically employ a short epidemic or pandemic simulation timeframe (e.g., 6-9 months), aiming to examine the short-term impact of public health interventions.

- *Compound and waning immunity*, combining both the immunisation and infection histories. This demands higher memory, continually increasing during the simulation as the agents are likely to accumulate multiple vaccination records and infections during the simulated time frame.

- *Complex phylogenetic structure* based on a non-homogeneous genome profile. Each infected agent is assigned a genome profile defined across thousands of nucleotide positions (e.g., 3,090 positions), partitioned between spike and non-spike regions and grouped in terms of antigenicity. Simulation of complex phylodynamics generates a memory-intensive computation task that demands a significant increase in both simulation time and computational resources.

- *Simulation across heterogeneous demographics*, with varying population sizes (including very dense populations). Simulating different demographics is needed to investigate conditions for the emergence of variants of concern (VoC), commonly observed in large heterogeneous populations.

### Performance and scalability

To improve computational efficiency, PhASE TraCE utilises multi-threading processing provided by the C++ OpenMP library. S21 Fig traces the average processing time of simulation runs performed on a high-performance computing cluster, for three different population sizes, ranging from approximately 230,000 agents (S21 Fig, panel A), to approximately 1,700,000 agents (S21 Fig, panel B), and approximately 8,000,000 agents (S21 Fig, panel C).

The average processing time per simulation day increases during the simulation: this occurs due to the increased memory (see the top row of S21 Fig). Nevertheless, there is a consistent reduction of the processing time when more CPUs are used per job. We note that, for a given number of CPUs, the average cumulative processing time linearly increases in proportion to the population size. For example, S22 Fig shows that when 8 CPUs are used, a simulation of a 365-day period across a population with 8 million agents completes in approximately 20,000 seconds (over 5.5 hours).

### Memory utilisation

An increasing size of the artificial population requires higher computation resources. This is because the stochastically generated agents are assigned several static demographic attributes, which are preserved during the simulation, and a number of dynamic phylogenetic and immunological attributes, which change during the simulation. These attributes contribute to both fixed memory and dynamic memory utilisation.

Agent attributes that utilise fixed memory include:

- **Demographic attributes**: household composition and age group.
- **Working group and school enrollment**: workplace for agents over the age of 18, or school for agents under the age of 18.

Agent attributes that affect dynamic memory utilisation during simulation include:

- **Vaccination history**: type of the administered vaccine, the total number of vaccination records and the time of vaccination.
- **Infection history**: the number of past infections and their recovery times.
- **Genome profiles**: infected agents are assigned a genome profile transferred from the source of infection, which continues to mutate during the agent's infectivity period.

As the simulation progresses, the number of stored immunological records grows significantly due to the increased number of infections and administered vaccinations. S23 Fig shows how the dynamic memory usage (in Gigabytes) increases with the larger number of stored immunological records across the population of 8 million agents.

## Acknowledgments

The simulations involved in this work were carried out on the high-performance computing cluster (Artemis) provided by the Sydney Informatics Hub at the University of Sydney. The authors would like to thank Christina M. Jamerlan, Tim Germann, Sara Del Valle, Michael Lachmann, Stuart Ridge, and Stuart Kauffman for many insightful discussions and comments. The authors are also grateful to Oliver Cliff, Cameron Zachreson and Nathan Harding for contributing to the development of AMTraC-19, a predecessor of PhASE TraCE.

## References

1. Hadfield J, Megill C, Bell SM, Huddleston J, Potter B, Callender C, et al.. Genomic epidemiology of SARS-CoV-2 with subsampling focused globally since pandemic start; 2024. Available from: `https://nextstrain.org/ncov/gisaid/global/all-time`.
2. Markov PV, Ghafari M, Beer M, Lythgoe K, Simmonds P, Stilianakis NI, et al. The evolution of SARS-CoV-2. Nature Reviews Microbiology. 2023;21(6):361–379. doi:10.1038/s41579-023-00878-2.
3. Smith AP. Nucleic acids to amino acids: DNA specifies protein. Nature Education;1(1):126.

4. Harvey WT, Carabelli AM, Jackson B, Gupta RK, Thomson EC, Harrison EM, et al. SARS-CoV-2 variants, spike mutations and immune escape. Nature Reviews Microbiology. 2021;19(7):409–424. doi:10.1038/s41579-021-00573-0.

5. Smith CC, Olsen KS, Gentry KM, Sambade M, Beck W, Garness J, et al. Landscape and selection of vaccine epitopes in SARS-CoV-2. Genome Medicine. 2021;13(1):101. doi:10.1186/s13073-021-00910-1.

6. Sender R, Bar-On YM, Gleizer S, Bernshtein B, Flamholz A, Phillips R, et al. The total number and mass of SARS-CoV-2 virions. Proceedings of the National Academy of Sciences. 2021;118(25):e2024815118. doi:10.1073/pnas.2024815118.

7. Obermeyer F, Jankowiak M, Barkas N, Schaffner SF, Pyle JD, Yurkovetskiy L, et al. Analysis of 6.4 million SARS-CoV-2 genomes identifies mutations associated with fitness. Science. 2022;376(6599):1327–1332. doi:10.1126/science.abm1208.

8. Kistler KE, Huddleston J, Bedford T. Rapid and parallel adaptive mutations in spike S1 drive clade success in SARS-CoV-2. Cell Host & Microbe. 2022;30(4):545–555.e4. doi:10.1016/j.chom.2022.03.018.

9. Thadani NN, Gurev S, Notin P, Youssef N, Rollins NJ, Ritter D, et al. Learning from prepandemic data to forecast viral escape. Nature. 2023;622(7984):818–825. doi:10.1038/s41586-023-06617-0.

10. Ghafari M, Hall M, Golubchik T, Ayoubkhani D, House T, MacIntyre-Cockett G, et al. Prevalence of persistent SARS-CoV-2 in a large community surveillance study. Nature. 2024;626(8001):1094–1101. doi:10.1038/s41586-024-07029-4.

11. Amicone M, Borges V, Alves MJ, Isidro J, Zé-Zé L, Duarte S, et al. Mutation rate of SARS-CoV-2 and emergence of mutators during experimental evolution. Evolution, Medicine, and Public Health. 2022;10(1):142–155. doi:10.1093/emph/eoac010.

12. Chang SL, et al. Modelling transmission and control of the COVID-19 pandemic in Australia. Nat Commun. 2020;11(1):5710. doi:10.1038/s41467-020-19393-6.

13. Attwood SW, Hill SC, Aanensen DM, Connor TR, Pybus OG. Phylogenetic and phylodynamic approaches to understanding and combating the early SARS-CoV-2 pandemic. Nature Reviews Genetics. 2022;23(9):547–562. doi:10.1038/s41576-022-00483-8.

14. Yang J, Skaro M, Chen J, Zhan D, Lyu L, Gay S, et al. The species coalescent indicates possible bat and pangolin origins of the COVID-19 pandemic. Scientific Reports. 2023;13(1):5571. doi:10.1038/s41598-023-32622-4.

15. Bull RA, Eden JS, Luciani F, McElroy K, Rawlinson WD, White PA. Contribution of Intra- and Interhost Dynamics to Norovirus Evolution. Journal of Virology. 2012;86(6):3219–3229. doi:10.1128/JVI.06712-11.

16. Voloch CM, Da Silva Francisco Jr R, De Almeida LGP, Brustolini OJ, Cardoso CC, Gerber AL, et al. Intra-host evolution during SARS-CoV-2 prolonged infection. Virus Evolution. 2021;7(2):veab078. doi:10.1093/ve/veab078.

17. Gonzalez-Reiche AS, Alshammary H, Schaefer S, Patel G, Polanco J, Carreño JM, et al. Sequential intrahost evolution and onward transmission of SARS-CoV-2 variants. Nature Communications. 2023;14(1):3235. doi:10.1038/s41467-023-38867-x.

18. Wilkinson SAJ, Richter A, Casey A, Osman H, Mirza JD, Stockton J, et al. Recurrent SARS-CoV-2 mutations in immunodeficient patients. Virus Evolution. 2022;8(2):veac050. doi:10.1093/ve/veac050.

19. Wilkinson S. Dataset for "Recurrent SARS-CoV-2 mutations in immunodeficient patients"; 2024. Available from: https://github.com/BioWilko/recurrent-sars-cov-2-mutations/tree/main/dataset.

20. Australian Bureau of Statistics. 2021 Census of Population and Housing; 2022. Available from: https://tablebuilder.abs.gov.au/.

21. Bureau of Infrastructure and Transport Research Economics. Airport traffic data (2022);. Available from: https://data.gov.au/dataset/ds-dga-cc5d888f-5850-47f3-815d-08289b22f5a8/details.

22. Australian Curriculum, Assessment and Authority (ACARA). School profiles and locations;. Available from: https://acara.edu.au/contact-us/acara-data-access.

23. Fair KM, Zachreson C, Prokopenko M. Creating a surrogate commuter network from Australian Bureau of Statistics census data. Scientific Data. 2019;6(1):150. doi:10.1038/s41597-019-0137-z.

24. Nguyen QD, Chang SL, Jamerlan CM, Prokopenko M. Measuring unequal distribution of pandemic severity across census years, variants of concern and interventions. Population Health Metrics. 2023;21(17):17.

25. Chang SL, Nguyen QD, Zachreson C, Cliff OM, Prokopenko M. AMTraC-19 Source Code: Agent-based Model of Transmission and Control of the COVID-19 pandemic in Australia;.

26. Chang SL, et al. Simulating Transmission Scenarios of the Delta Variant of SARS-CoV-2 in Australia. Front Public Health. 2022;10.

27. Chang SL, et al. Persistence of the Omicron variant of SARS-CoV-2 in Australia: The impact of fluctuating social distancing. PLOS Global Public Health. 2023;3(4):e0001427. doi:10.1371/journal.pgph.0001427.

28. Chang SL, Nguyen QD, Suster CJE, Jamerlan CM, Rockett RJ, Sintchenko V, et al.. Impact of opinion dynamics on recurrent pandemic waves: balancing risk aversion and peer pressure; 2024. Available from: https://arxiv.org/abs/2408.00011.

29. Center for Disease Control and Prevention. What is COVID-19 Reinfection?; 2023. https://www.cdc.gov/coronavirus/2019-ncov/your-health/reinfection.html.

30. New South Wales Health. NSW Respiratory Surveillance Report - week ending 30 July 2022; 2022. https://www.health.nsw.gov.au/Infectious/covid-19/Documents/weekly-covid-overview-20220730.pdf.

31. CDC. About Chronic Symptoms Following Infections; 2023. https://www.cdc.gov/chronic-symptoms-following-infections/about/.

32. Zachreson C, et al. How will mass-vaccination change COVID-19 lockdown requirements in Australia? The Lancet Regional Health - Western Pacific. 2021;14:100224. doi:10.1016/j.lanwpc.2021.100224.

33. Nguyen QD, Prokopenko M. A general framework for optimising cost-effectiveness of pandemic response under partial intervention measures. Scientific Reports. 2022;12(1):19482. doi:10.1038/s41598-022-23668-x.

34. Lin C, Bier B, Tu R, Paat JJ, Tu P. Vaccinated Yet Booster-Hesitant: Perspectives from Boosted, Non-Boosted, and Unvaccinated Individuals. Vaccines. 2023;11(3):550. doi:10.3390/vaccines11030550.

35. Cao L, Lou J, Chan SY, Zheng H, Liu C, Zhao S, et al. Rapid evaluation of COVID-19 vaccine effectiveness against symptomatic infection with SARS-CoV-2 variants by analysis of genetic distance. Nature Medicine. 2022;28(8):1715–1722. doi:10.1038/s41591-022-01877-1.

36. Szanyi J, et al. A log-odds system for waning and boosting of COVID-19 vaccine effectiveness. Vaccine. 2022;40(28):3821–3824.

37. Larkin H. Hybrid Immunity More Protective Than Prior SARS-CoV-2 Infection Alone. JAMA. 2023;329(7):531. doi:10.1001/jama.2023.0743.

38. World Health Organization. Interim statement on hybrid immunity and increasing pop-
ulation seroprevalence rates;. Available from: https://www.who.int/news/item/01-06-2022-interim-statement-on-hybrid-immunity-and-increasing-population

39. Du L, He Y, Zhou Y, Liu S, Zheng BJ, Jiang S. The spike protein of SARS-CoV — a target for vaccine and therapeutic development. Nature Reviews Microbiology. 2009;7(3):226–236. doi:10.1038/nrmicro2090.

40. Cankat S, Demael MU, Swadling L. In search of a pan-coronavirus vaccine: next-generation vaccine design and immune mechanisms. Cellular & Molecular Immunology. 2024;21(2):103–118. doi:10.1038/s41423-023-01116-8.

41. Kojima N, Klausner JD. Protective immunity after recovery from SARS-CoV-2 infection. The Lancet Infectious Diseases. 2022;22(1):12–14. doi:10.1016/S1473-3099(21)00676-9.

42. Kodera S, Rashed EA, Hirata A. Estimation of Real-World Vaccination Effectiveness of mRNA COVID-19 Vaccines against Delta and Omicron Variants in Japan. Vaccines. 2022;10(3):430. doi:10.3390/vaccines10030430.

43. Cromer D, Steain M, Reynaldi A, Schlub TE, Khan SR, Sasson SC, et al. Predicting vaccine effectiveness against severe COVID-19 over time and against variants: a meta-analysis. Nature Communications. 2023;14(1):1633. doi:10.1038/s41467-023-37176-7.

44. Menni C, May A, Polidori L, Louca P, Wolf J, Capdevila J, et al. COVID-19 vaccine waning and effectiveness and side-effects of boosters: a prospective community study from the ZOE COVID Study. The Lancet Infectious Diseases. 2022;22(7):1002–1010. doi:10.1016/S1473-3099(22)00146-3.

45. Khoury DS, Cromer D, Reynaldi A, Schlub TE, Wheatley AK, Juno JA, et al. Neutralizing antibody levels are highly predictive of immune protection from symptomatic SARS-CoV-2 infection. Nature Medicine. 2021;27(7):1205–1211. doi:10.1038/s41591-021-01377-8.

46. Menegale F, Manica M, Zardini A, Guzzetta G, Marziano V, d'Andrea V, et al. Evaluation of Waning of SARS-CoV-2 Vaccine–Induced Immunity: A Systematic Review and Meta-analysis. JAMA Network Open. 2023;6(5):e2310650. doi:10.1001/jamanetworkopen.2023.10650.

47. Feng A, Obolski U, Stone L, He D. Modelling COVID-19 vaccine breakthrough infections in highly vaccinated Israel—The effects of waning immunity and third vaccination dose. PLOS Global Public Health. 2022;2(11):e0001211. doi:10.1371/journal.pgph.0001211.

48. Harris RJ, Hall JA, Zaidi A, Andrews NJ, Dunbar JK, Dabrera G. Effect of Vaccination on Household Transmission of SARS-CoV-2 in England. New England Journal of Medicine. 2021;385(8):759–760. doi:10.1056/NEJMc2107717.

49. Shrestha NK, Burke PC, Nowacki AS, Terpeluk P, Gordon SM. Necessity of Coronavirus Disease 2019 (COVID-19) Vaccination in Persons Who Have Already Had COVID-19. Clinical Infectious Diseases: An Official Publication of the Infectious Diseases Society of America. 2022;75(1):e662–e671. doi:10.1093/cid/ciac022.

50. Shrestha NK, Shrestha P, Burke PC, Nowacki AS, Terpeluk P, Gordon SM. Coronavirus Disease 2019 Vaccine Boosting in Previously Infected or Vaccinated Individuals. Clinical Infectious Diseases. 2022;75(12):2169–2177. doi:10.1093/cid/ciac327.

51. Franchi M, Pellegrini G, Cereda D, Bortolan F, Leoni O, Pavesi G, et al. Natural and vaccine-induced immunity are equivalent for the protection against SARS-CoV-2 infection. Journal of Infection and Public Health. 2023;16(8):1137–1141. doi:10.1016/j.jiph.2023.05.018.
